# A rectal cancer feasibility study with an embedded phase III trial design assessing magnetic resonance tumour regression grade (mrTRG) as a novel biomarker to stratify management by good and poor response to chemoradiotherapy (TRIGGER): study protocol for a randomised controlled trial

**DOI:** 10.1186/s13063-017-2085-2

**Published:** 2017-08-29

**Authors:** Nick J. Battersby, Mit Dattani, Sheela Rao, David Cunningham, Diana Tait, Richard Adams, Brendan J. Moran, Shelize Khakoo, Paris Tekkis, Shahnawaz Rasheed, Alex Mirnezami, Philip Quirke, Nicholas P. West, Iris Nagtegaal, Irene Chong, Anguraj Sadanandam, Nicola Valeri, Karen Thomas, Michelle Frost, Gina Brown

**Affiliations:** 1 Pelican Cancer Foundation, The Ark, Basingstoke, RG24 9NN UK; 2 North Hampshire Hospital Foundation Trust, Basingstoke, RG24 9NA UK; 3 Department of Medicine Royal Marsden Hospital Sutton, Sutton, SM2 5PT UK; 4 Velindre Cancer Centre Velindre Hospital Cardiff, Cardiff, CF4 7XL UK; 5 Gastrointestinal Unit Royal Marsden Hospital Sutton, Sutton, SM2 5PT UK; 6 Department of Colorectal Surgery, Royal Marsden Hospital London, London, SW3 6JJ UK; 7 Department of Surgery and Department for Tissue Microarray analysis, University of Southampton, Southampton, SO16 6YD UK; 8 Pathology and Tumour Biology, Leeds Institute of Cancer and Pathology, Wellcome Trust Brenner Building, St. James’s University Hospital, Leeds, LS9 7TF UK; 9 Department of Pathology Radboud University, Nijmegen, 6500HB Netherlands; 10 Division of Molecular Pathology Institute of Cancer Research, London, SW3 6JB UK; 11 Statistics Unit, R&D Royal Marsden Hospital Sutton, Sutton, SM2 5PT UK; 12 Department of Radiology, Royal Marsden Hospital Sutton, Sutton, SM2 5PT UK

**Keywords:** Randomised control trial, Chemoradiotherapy, Rectal cancer, mrTRG, Complete response, Tumour regression, Tumour cell density

## Abstract

**Background:**

Pre-operative chemoradiotherapy (CRT) for MRI-defined, locally advanced rectal cancer is primarily intended to reduce local recurrence rates by downstaging tumours, enabling an improved likelihood of curative resection. However, in a subset of patients complete tumour regression occurs implying that no viable tumour is present within the surgical specimen. This raises the possibility that surgery may have been avoided. It is also recognised that response to CRT is a key determinant of prognosis. Recent radiological advances enable this response to be assessed pre-operatively using the MRI tumour regression grade (mrTRG). Potentially, this allows modification of the baseline MRI-derived treatment strategy. Hence, in a ‘good’ mrTRG responder, with little or no evidence of tumour, surgery may be deferred. Conversely, a ‘poor response’ identifies an adverse prognostic group which may benefit from additional pre-operative therapy.

**Methods/design:**

TRIGGER is a multicentre, open, interventional, randomised control feasibility study with an embedded phase III design. Patients with MRI-defined, locally advanced rectal adenocarcinoma deemed to require CRT will be eligible for recruitment. During CRT, patients will be randomised (1:2) between conventional management, according to baseline MRI, versus mrTRG-directed management. The primary endpoint of the feasibility phase is to assess the rate of patient recruitment and randomisation. Secondary endpoints include the rate of unit recruitment, acute drug toxicity, reproducibility of mrTRG reporting, surgical morbidity, pathological circumferential resection margin involvement, pathology regression grade, residual tumour cell density and surgical/specimen quality rates. The phase III trial will focus on long-term safety, regrowth rates, oncological survival analysis, quality of life and health economics analysis.

**Discussion:**

The TRIGGER trial aims to determine whether patients with locally advanced rectal cancer can be recruited and subsequently randomised into a control trial that offers MRI-directed patient management according to radiological response to CRT (mrTRG). The feasibility study will inform a phase III trial design investigating stratified treatment of good and poor responders according to 3-year disease-free survival, colostomy-free survival as well as an increase in cases managed without a major resection.

**Trial registration:**

ClinicalTrials.gov, ID: NCT02704520. Registered on 5 February 2016.

**Electronic supplementary material:**

The online version of this article (doi:10.1186/s13063-017-2085-2) contains supplementary material, which is available to authorized users.

## Background

Currently, 45–55% of rectal cancer patients receive pre-operative chemoradiotherapy (CRT) for locally advanced disease at presentation. Pre-operative CRT is given in order to downstage the tumour. It has three potential benefits in high-risk rectal cancers that respond to treatment: an increased likelihood of a clear circumferential resection margin (CRM); less radical surgery than was initially planned on the baseline staging magnetic resonance imaging (MRI) [1]; and in a small subset of patients in whom there is no clinical or radiological evidence of tumour, the option of deferral of surgery [2, 3]. However, the response to treatment is highly variable, with up to 30% of patients achieving a complete or near-complete response as judged by pathological tumour regression grade (pTRG). Prognostically, a good pTRG is associated with significantly lower local recurrence, distant metastatic rates and with higher overall survival compared with a poor pTRG [4, 5].

The need for a validated means of assessing response to treatment is widely accepted [6] but there has been no reliable method of assessing this response in the pre-operative setting to date. Therefore, the current consensus is that the baseline MRI staging should be the standard of care that is used to define the plane of surgery, regardless of any assessment of treatment response [7]. Recently, a 5-point MRI tumour regression grade (mrTRG), which most closely resembles the Mandard pTRG system [8], has been developed [9]. The basic principle of both grading systems relates to the ratio of tumour to fibrosis following CRT (Table 1). Patients with a poor CRT response have a 5-year overall survival of 27% versus 72% (*p* = 0.001) for a good CRT response [10]. This novel imaging biomarker has been reliable and reproducible between multiple independent radiologists, and validated against both pathology and survival outcomes [10–12]. However, there is currently insufficient evidence that this information can be safely used to alter treatment decisions.

**Table 1:** Magnetic resonance imaging tumour regression grade (mrTRG)

mrTRG 1 – Complete radiological response (linear scar only) mrTRG 2 – Good response (dense fibrosis, no obvious tumour signal) mrTRG 3 – Moderate response (>50% fibrosis and visible intermediate signal) mrTRG 4 – Slight response (mostly tumour) mrTRG 5 – No response/regrowth of tumour	

### The ‘good-response’ group (mrTRG I and II)

The complications of surgery can be considerable. The 90-day mortality rate from a major bowel resection is 3–6% [13], significant morbidity is frequent and long-term functional impairment is common, even in the presence of restorative surgery [14–16]. When a pathological complete response (pCR) to CRT occurs, with no residual cancer identified, this raises the possibility that the risks of surgery may have been avoided by watchful waiting or deferral of surgery [17]. Pioneered by Professor Angelita Habr-Gama, several cohorts have demonstrated this to be feasible and safe; avoiding radical surgery by close surveillance of selected patients in whom there is clinical disappearance of the tumour following CRT [2, 3, 18]. Termed as a clinical complete response (cCR), the combination of digital rectal examination, endoscopy and imaging are used to define and monitor patients under a deferral of surgery protocol as a surrogate for a pCR, thereby avoiding radical surgery [19–21]. However, current techniques for assessing a cCR are unreliable, and up to two thirds of patients are not identified pre-operatively [22–25]. By using the novel mrTRG biomarker prospectively in a randomised controlled study, it may be possible to determine the precise safety of this approach, whilst increasing the proportion of cCR patients identified using an objective and validated tool.

A good tumour response on post-CRT MRI (mrTRG I and II) may enable a nonoperative approach to rectal cancer. Consequently, patients may have a reduced overall morbidity and mortality [26], as well as an improvement in quality of life (QoL).

### The ‘poor-response’ group (mrTRG III–V)

The TRIGGER trial will offer poor responders consolidation therapy using a fluorouracil (5-FU)-based regimen combined with oxaliplatin (infusional 5-FU or capecitabine with oxaliplatin, FOLFOX or CAPOX). These regimens have been shown to be effective systemic treatments for colorectal malignancy and they are recommended by most international guidelines [27, 28], usually in the post-operative setting. However, a prospective study by Garcia-Aguilar et al. found that FOLFOX can be used as pre-operative consolidation therapy, along with conventional CRT, without evidence of additional toxicity [29]. In the SOCRATES trial, patients received a similar regimen of radiotherapy and oxaliplatin; however, capecitabine (the 5-FU oral prodrug) was used [30]. The CAPOX treatment regime was offered to patients with locally advanced rectal cancer, 82 of the 83 patients enrolled into the trial completed the pre-operative CAPOX regimen and 78 patients proceeded to surgery. Similar compliance rates were seen with pre-operative CAPOX-RT in a German study, where compliance rates were 96% [31]. In TRIGGER, ‘poor responders’ (mrTRG III–V) will be offered systemic oxaliplatin and either 5-FU or capecitabine pre-operatively. In this high-risk subgroup of patients, the earlier introduction of systemic therapy appears not only to improve compliance but also tumour response rates and the rates of distant metastatic disease [32].

## Methods/design

### Trial overview

**Fig. 1:**
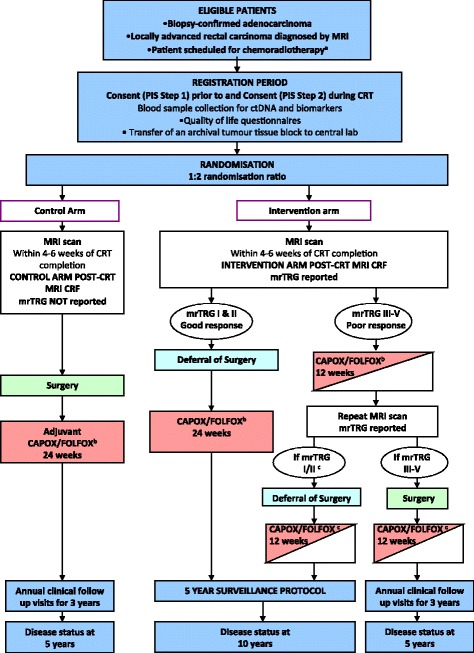
**a** Scheduled to receive 45 Gy–55 Gy long-course radiotherapy. **b** Treatment decision should be made prior to registration (planned choice is a randomisation stratification variable). Medical oncologist may choose to use CAPOX or FOLFOX, or single-agent capecitabine or 5-FU if concomitant use of oxaliplatin is contraindicated. **c** Patient defers surgery then the remaining 12 weeks of chemotherapy should be given as soon as possible following the repeat magnetic resonance imaging (MRI) scan and multidisciplinary team (MDT) meeting

The TRIGGER trial is a multicentre, open, randomised control trial (Additional file 1). Randomisation is 2:1 in favour of mrTRG-directed management. We anticipate that 30 centres will recruit to the study. The phase III primary endpoints will compare outcomes by intention-to-treat analysis between the control arm and the interventional arm. There are two prospective subtrials, the first involves the ‘good-response’ group and the second involves the ‘poor-response’ group. The trial flow chart is shown in Fig. 1 (Additional files 2 and 3). The trial has been developed in accordance with Standard Protocol Items: Recommendations for Interventional Trials (SPIRIT) guidelines (Additional file 4).

### Study population and eligibility criteria

Eligible patients are 18 years or older at the time of diagnosis, able to give informed consent, and have a biopsy-confirmed adenocarcinoma 0–15 cm from the anal verge measured on MRI or rigid sigmoidoscopy. The initial staging MRI must indicate locally advanced rectal carcinoma, defined here as: mrCRM unsafe, ≥mrT3c (>5 mm beyond muscularis propria), mrN1c (extramural tumour deposit not typical of lymph nodes) or MRI-assessed evidence of extramural venous invasion (mrEMVI)-positive disease (extramural venous invasion on MRI). The patient must be deemed to require chemoradiotherapy and scheduled to receive 45 Gy–55 Gy long-course radiotherapy.

Patients will be ineligible if there is evidence of distant metastatic disease, including resectable liver metastases; MRI contraindications; intolerance or contraindication to planned chemoradiotherapy (CRT); receive alternative cytotoxic or investigational drug treatment outside of protocol stipulation; pregnancy; breastfeeding; other malignant disease within the preceding 5 years with the exception of non-melanomatous skin cancer, carcinoma in situ and early stage disease with <5% recurrence risk. Some centres report the controversial practice of offering CRT to early stage rectal cancer patients with the specific intention of avoiding an APE by achieving a cCR [20]. Only patients with locally advanced disease are eligible for TRIGGER because the multi-European centre low rectal cancer study (MERCURY II) found that patients with early stage low rectal cancer (cancer that did not breach the CRM or low rectal plane on MRI) were able to undergo a restorative resection the majority of the time (108/166 – 65%) [33]. Without radiotherapy this group reported a pCRM rate of 2% and these patients avoided the morbidity and functional impairment that is associated with radiotherapy.

This is a multicentre study and currently seven trials units are open to recruitment; we anticipate this will be sufficient to deliver the feasibility study. A further 20 centres are undergoing the trials registration and quality assurance process with a view to participating in feasibility and/or the phase III trial. Participating centres must have an established multidisciplinary team (MDT) that includes a minimum of one oncologist, one histopathologist, one radiologist and one surgeon, and be able to accommodate and deliver all aspects of the protocol, including patient follow-up, particularly the follow-up requirements for safe deferral of surgery. The centre is required to meet eligibility criteria and trial conduct must comply with the protocol as agreed by the sponsor, the Medicines and Healthcare products Regulatory Agency (MHRA) and the Multicentre Ethical Committee (MREC). Trial centres will encompass both teaching and district general hospitals to enhance generalisability.

## Study objectives

### Feasibility study objectives

- Patient recruitment – the number of eligible patients willing to enter the study, and the rate of concordance with the allocated treatment plan
- Centre recruitment – acceptability of the trial concept and the protocol to each unit as well as the ability of radiologists to reliably assess response (agreement with central radiologist measured using a training set with kappa >0.7)
- Evaluate the reproducibility of mrTRG by assessing the strength of the agreement between the recruiting radiologists and the central radiologist
- Assess the clinical, radiological response rate in the control versus intervention arm. Pathological response rate will also be reported and compared when possible
- Evaluate safety by assessing acute drug toxicity and 30-day surgical morbidity
- Compare the control arm versus the intervention arm in terms of pCRM involvement and specimen quality as a proxy for quality of surgery (quality graded by plane of surgery as: complete clinical response (no specimen), mesorectal, intramesorectal, and muscularis propria). The assessment will include patients who undergo surgery for tumour regrowth following initial management with deferral of surgery

### Phase III study objectives

- The *primary objective* of the phase III trial will be to compare 3-year disease-free survival (DFS) in the control arm versus the mrTRG-directed management arm
- Overall survival (OS), colostomy-free survival (CFS), distant (DR) and local recurrence (LR) in the control arm versus the mrTRG-directed management arm, and tumour regrowth rates in patients treated with deferral of surgery
- QoL comparison
- Cost-utility analysis

### Sample size

The feasibility study is designed to determine whether proceeding to a phase III study is realistic. Using the phase III primary endpoint of 3-year DFS, an expected improvement in DFS for the intervention group by intention-to-treat analysis would be 82% from 74% (i.e. a hazard ratio of 0.66) with 80% power and a 5% two-sided level of statistical significance. This would require 633 patients: 422 in the intervention arm and 211 in the control arm based on a 2:1 allocation ratio to be recruited over 3 to 5 years with at least 3 years’ follow-up. Hence, in order for the phase III trial to be viable, recruitment rates of at least 5–6 per month (to recruit 633 patients in 5 years) and, ideally, 11 per month (to recruit 633 patients in 3 years) should be achievable. The feasibility study was, therefore, designed to test the null hypothesis that the maximum possible recruitment rate is no more than 6 per month, and power calculations were performed under the assumption that the a rate of 11 per month can be achieved. The type-I error significance threshold (alpha) was set at 0.1, with a type-II error (*β*) at 5% with the resulting power (1 − *β*) at 95% (calculated using simulation). In order to allow for a set-up period of 6 months we will recruit for a total of 10 months, with recruitment rates for the last 4 months used to test the primary hypothesis. Thus, during the final 4 months recruitment is expected to be 11 patients per month and can be no less than 6 patients per month (6 per month results in 5.5 years total recruitment time).

### Randomisation

Patients identified at the MDT as potentially eligible for TRIGGER are invited and may consent to register prior to CRT. Basic demographic and clinical information will be collected. During CRT, eligible patients are then considered for randomisation. Registered patients who are not randomised are retained along with the reason for nonrandomisation, so that the causes for ‘drop-out’ and estimates of generalisability can be established.

Randomisation is performed centrally, at the TRIGGER trial office, by a computer-based randomisation algorithm. Patients are randomised 2:1 in favour of mrTRG-directed management (interventional arm) over best current management (control arm) based on the index staging MRI. To avoid chance imbalance in the two arms, randomisation will factor-in stratification variables: recruiting site, mrEMVI status, tumour height, and planned choice of systemic chemotherapy.

Following randomisation, the radiologist from the recruiting centre will report the mrTRG for the intervention arm but not for the control arm. It is not possible to blind the patient, radiologist, surgeon or pathologist, but these assigned members of the MDT will be trained to manage patients according to the TRIGGER protocol (v5.0) and to prospectively proforma report findings. To further enhance the quality, reliability and reproducibility of the data the radiologists and pathologists will centrally review image quality, surgical specimen quality and pathological tumour regression.

### Intervention

The intervention in this study results from mrTRG-directed management. To report the mrTRG, the baseline and post-treatment MRI scans will be reported according to standardised proformas and the following imaging standards are required:

- Positioning and patient preparation – buscopan 20 mg (intra-muscularly) to be given, or other suitable antispasmodic agents according to local protocol. Superior saturation bands/REST slabs for adequate abdominal motion suppression and anterior SAT band to be used in conjunction with AP-phase encoding direction to reduce image degradation due to abdominal wall motion. Firm surface coil placement – lower edge of coil 10 cm below symphysis pubis
- T2-weighted images – TR >3500 ms, TE >80 ms
- Adequate signal to noise – at least four acquisitions and 6 min per sequence
- Adequately high resolution scans – field of view and matrix parameters should not exceed a pixel size of 0.6 mm × 0.6 mm; either 200 mm × 200 mm with 384 × 384 matrix or 160 mm × 160 mm with a 256 × matrix. Slice-thickness 3 mm giving a 0.6 mm × 0.6 mm × 3 mm = 1.1-mm^3^ voxel
- Adequate coverage – high-resolution scans extend to at least 5 cm above the top of tumour. Any discontinuous deposits seen on sagittal view are also covered on the high resolution axial views

The post-CRT MRI should be performed within 4–6 weeks, and no later than 10 weeks of CRT completion.

### Control Arm

The flow chart for the control group is shown in Fig. 1. The mrTRG is not reported for the control arm and surgery is performed within 6–12 weeks from CRT completion according to the baseline MRI staging. The follow-up and Clinical Report Form (CRF) assessment schedule is summarised in Table 2.

**Table 2:** Assessment schedule summary: control arm

Control arm		Registration period	Intervention phase						Annual follow-up			Disease status^g^
Visit type	Prior to patient entry	Registration	Randomisation (baseline)	Post CRT	MDT	surgery	Surgical follow-up	Adjuvant chemotherapy for 24 weeks	12	24	36	60
Timelines		≤4 weeks prior to CRT	During CRT	4–6 weeks post CRT		6–12 weeks post CRT	6 weeks post surgery	Toxicity assessed at end of each chemotherapy cycle	Months from end of CRT			
Informed consent^a^		X	X									
Check eligibility criteria		X	X									
Diagnosis, history and clinical assessment		X										
Randomisation			X									
Quality of life		X							X		X	
Chemoradiotherapy			X									
Blood sample^b^		X		X			X		X	X	X	
Baseline MRI	X											
Restaging MRI^c^				X								
Surgery						X						
Surgical morbidity^f^							X		X			
Pathology^d^							X					
Chemotherapy								X end of each cycle				
Toxicity assessment								X end of each cycle	X			
Annual follow-up						X			X	X	X	X
Adverse events^e^				X		X	X	X end of each cycle				
Concurrent medications		X	X	X		X	X	X end of each cycle				

*CRT* pre-operative chemoradiotherapy, *MDT* multidisciplinary team, *MRI* magnetic resonance imaging

The X also denotes that Clinical Report Forms (CRFs) need completing

^a^Eligible subjects will be asked to provide written informed consent at registration and before randomisation

^b^If patient has consented to additional blood sample collection for research

^c^The post-CRT MRI should to be performed within 4–6 weeks (maximum of 10 weeks) from completion of CRT

^d^Resected specimen will be prepared and evaluated using a standardised protocol

^e^All adverse events will be recorded from the date the post-CRT MRI scan is performed until 30 days after the last dose of chemotherapy is administered during the intervention phase of the trial

^f^Both early (4–6 weeks) and late surgical complications (at 12 months) will be recorded

^g^Disease status at 5 years (does not require clinic visit): alive without metastatic or recurrent disease, alive with metastatic and/or recurrent disease (date diagnosed), dead (date of death)

### Experimental intervention – good response (mrTRG I and II)

The flow chart for the good-response group of the intervention arm is shown in Fig. 1. This group has been randomised to mrTRG-directed management and, where the post-treatment MRI suggests a good response (mrTRG I or II), the option of deferral of surgery is discussed with the patient and intensive active monitoring is undertaken. This will involve a combination of clinical assessments, imaging surveillance, and endoscopy at regular intervals, as per the schedule shown in Table 3. These patients will subsequently receive 24 weeks of chemotherapy (eight cycles of CAPOX or 12 cycles of FOLFOX) and this should start within 12 weeks of CRT completion. The follow-up and CRF assessment schedule is summarised in Table 4.

**Table 3:** Deferral of surgery follow-up protocol

Time line from *end of CRT*	Visit window	Clinic OPA^b^	PROM^c^	Scans	Endoscopy
6 months^a,d^	±1 month	X		MRI	Flex sig
9 months^d^	± 1 month	X		MRI	Flex sig
1 year^d^	± 1 month	X	EORTC QLQ-C30, LARS, EQ-5D	MRI CT	Colonoscopy
1 year 3 months	± 1 month	X			
1 year 6 months^d^	± 1 month	X		MRI	Flex sig
1 year 9 months	± 1 month	X			
2 years^e^	± 1 month	X		MRI CT	Flex sig
2 years 6 months^e^	± 1 month	X			
3 years^d^	± 2 months	X	EORTC QLQ-C30, LARS, EQ-5D	MRI CT	Flex sig
3 years 6 months	± 2 months	X			
4 years^d^	± 2 months	X		MRI	Flex sig
4 years 6 months	± 2 months	X			
5 years^d^	± 3 months	X	EORTC QLQ-C30, LARS, EQ-5D	MRI	Colonoscopy

^*a*^ *This visit should take place once the patient has completed chemotherapy*. It is recommended that a computed tomography (CT) scan is also performed following the completion of chemotherapy as is usual practice

^b^Each clinic outpatient appointment (OPA) should include a digital rectal exam and CEA (tumour marker) blood test

^c^Quality of life Case Record Form (CRF): EORTC QLQ-C30, European Organisation for Research and Treatment of Cancer Quality of Life Questionnaire-Core 30, v3; LARS, Low Anterior Resection Syndrome Score; EQ-5D, EuroQol Group five dimensions Health Questionnaire

^d^If patient has consented to the additional blood sample collection for research (circulating tumour DNA and markers of cell proliferation and apoptosis) then samples collected during the clinic outpatient appointment, ideally at the same time as the routine blood tests are performed

*CRT* pre-operative chemoradiotherapy, *Flex sig* flexible sigmoidoscopy, *PROM* patient-reported outcome measure

**Table 4:** Intervention arm – ‘good response’ to chemoradiotherapy

Intervention arm – good responders		Registration period	Intervention phase					Follow-up	Disease status^g^
Visit type	Prior to patient entry	Registration	Randomisation (baseline)	Post CRT	MDT	No surgery	Chemotherapy for 24 weeks^f^	Follow surveillance schedule for patients who defer surgery for a period of *5 years* from end of chemotherapy (Table 3). If surgery is undertaken (patient declines deferral of surgery or regrowth is detected during follow-up) then the follow-up schedule for the control arm should be used. The Surgery and Surgical Morbidity CRFs should be completed as per control schedule. In the case of regrowth a Surveillance CRF should also be completed.	10 years
Timelines		≤4 weeks prior to CRT	During CRT	4–6 weeks post CRT	Review of restaging MRI including mrTRG reporting. mrTRG-directed management results in option of deferral of surgery (mrTRG I and II). The option of deferral of surgery is discussed with patient		≤12 weeks post CRT. Toxicity assessed at end of each cycle during chemotherapy		From end of CRT
Informed consent^a^		X	X						
Check eligibility criteria		X	X						
Diagnosis and clinical assessment		X							
Randomisation			X						
Quality of life		X							
Chemoradiotherapy			X						
Blood sample^b^		X		X					
Baseline MRI	X								
Restaging MRI^c^				X			X		
Pathology^d^									
Chemotherapy							X end of each cycle		
Toxicity assessment							X end of each cycle		
Annual follow-up									X
Adverse events^e^				X			X end of each cycle		
Concurrent medications		X	X	X			X end of each cycle		

*CRT* pre-operative chemoradiotherapy, *MDT* multidisciplinary team, *MRI* magnetic resonance imaging, *mrTRG* MRI tumour regression grade

The X also denotes that Clinical Report Forms (CRFs) need completing

^a^Eligible subjects will be asked to provide written informed consent at registration and before randomisation

^b^If patient has consented to additional blood sample collection for research

^c^The post-CRT MRI should to be performed within 4–6 weeks (maximum of 10 weeks) from completion of CRT. A further MRI should be performed mid-way through chemotherapy treatment at approximately 12 weeks

^d^Resected specimen will be prepared and evaluated using a standardised protocol

^e^All adverse events will be recorded from the date the post-CRT MRI scan is performed until 30 days after the last dose of chemotherapy is administered during the intervention phase of the trial

^f^Initial staging indicated these tumours were locally advanced; therefore, all patients are offered a systemic chemotherapy regimen equivalent to post-operative adjuvant chemotherapy. If regrowth occurs during chemotherapy patient should proceed to surgery and discuss the pathology at MDT to decide if remaining cycles should be given post-operatively

^g^Disease status at 10 years (does not require clinic visit): alive without metastatic or recurrent disease, alive with metastatic and/or recurrent disease (date diagnosed), dead (date of death)

#### Detection of local regrowth

**Fig. 2:**
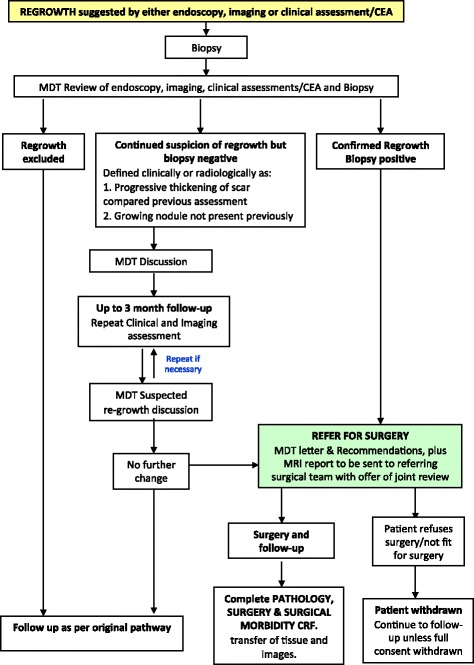
Flow chart for the management of tumour regrowth after initial deferral of surgery

A regrowth is a defined as recorded evidence of disease at the site of the primary tumour in a patient with previous investigations suggesting an apparent cCR. Patients with evidence of clinical or radiological local regrowth or pelvic relapse must be treated at least as urgently as a primary rectal cancer by the surgical team (Fig. 2). A biopsy identifying regrowth of adenocarcinoma should ideally be sought before planning surgical intervention and, in equivocal cases, a MDT discussion should take place to decide on the best course of action. If a patient refuses, or is not fit for, surgery then a ‘Discontinuation of Trial Treatment Form’ should be completed and the patient followed up unless they withdraw consent to further data collection. Follow-up will also monitor for *unsalvageable regrowth* – a tumour with radiological evidence of a good response that progresses following initial deferral of surgery management. The extent of progression means that the patient develops *inoperable disease or pCRM involvement*.

### Experimental intervention – poor response (mrTRG III–V)

The flow chart for the poor response group of the intervention arm is shown in Fig. 1. This group has been randomised to mrTRG-directed management and the post-treatment MRI suggests a poor response (mrTRG III–V). The option of intensified additional chemotherapy is discussed with the patient, and if the patient consents, 12 weeks of chemotherapy will be given (four cycles of CAPOX or six cycles of FOLFOX); this should start within 12 weeks of CRT completion. Following the completion of 12 weeks of chemotherapy, a repeat MRI should be performed 4–6 weeks later and mrTRG reported again. If this MRI suggests a good response (mrTRG I or II), deferral of surgery can be discussed; otherwise, the patient should proceed to surgery according to the baseline and post-treatment imaging. The follow-up and CRF assessment schedule is summarised in Table 5. The SPIRIT diagram for both randomisation arms and intervention arm sub-groups is shown in Table 6.

**Table 5:** Intervention arm – ‘poor response’ to chemoradiotherapy

Intervention arm – poor responders		Registration period	Intervention phase								Annual follow-up			Disease status^g^
Visit type	Prior to patient entry	Registration	Randomisation (baseline)	Post CRT	MDT	Chemo-therapy for 12 weeks	MDT	surgery	Surgical follow-up	Adjuvant chemotherapy for 12 weeks	12	24	36	60
Timelines		≤4 weeks prior to CRT	During CRT	4–6 weeks post CRT		≤12 weeks post CRT. Toxicity assessed at end of each cycle		6–12 weeks after pre-op chemo	6 weeks post surgery	Toxicity assessed at end of each cycle during chemotherapy	Months from end of CRT			
Informed consent^a^		X	X											
Check eligibility criteria		X	X											
Diagnosis and clinical assessment		X												
Randomisation			X											
Quality of life		X									X		X	
Chemoradiotherapy			X											
Blood sample^b^		X		X		X			X		X	X	X	
Baseline MRI	X													
Restaging MRI^c^				X		X ^i^								
Surgery								X						
Surgical morbidity^f^									X		X			
Pathology^d^									X					
Chemotherapy^h^						X end of each cycle				X end of each cycle				
Toxicity assessment						X end of each cycle				X end of each cycle	X			
Annual follow-up^g^											X	X	X	X
Adverse events^e^				X		X end of each cycle				X end of each cycle				
Concurrent medications		X	X	X		X end of each cycle		X	X	X end of each cycle	X			

*CRT* preoperative chemoradiotherapy, *MDT* multidisciplinary team, *MRI* magnetic resonance imaging

The X also denotes that Clinical Report Forms (CRFs) need completing – tick or initial the boxes as the CRFs are completed

^a^Eligible subjects will be asked to provide written informed consent at registration and before randomisation

^b^If patient has consented to additional blood sample collection for research

^c^The post-CRT MRI should to be performed within 4–6 weeks (maximum of 10 weeks) from completion of CRT

^d^Resected specimen will be prepared and evaluated using a standardised protocol

^e^All adverse events will be recorded from the date the post-CRT MRI scan is performed until 30 days after the last dose of chemotherapy is administered during the intervention phase of the trial

^f^Both early (4–6 weeks) and late surgical complications (at 12 months) will be recorded

^g^Disease status at 5 years (does not require clinic visit): alive without metastatic or recurrent disease, alive with metastatic and/or recurrent disease (date diagnosed), dead (date of death)

^h^Chemotherapy toxicity is assessed every 6 weeks during chemotherapy treatment. 12 weeks (6 cycles of FOLFOX or 4 cycles of CAPOX) are given pre-operatively and 12 weeks (6 cycles of FOLFOX or 4 cycles of CAPOX) are given post-operatively

^i^Further MRI scan should be performed within 4–6 weeks from completion of pre-operative chemotherapy and mrTRG reported

**Table 6:**
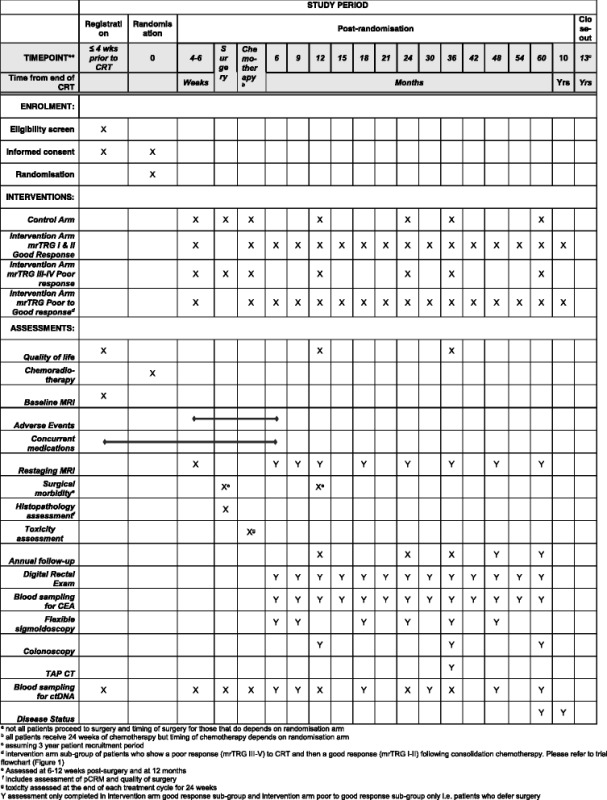
SPIRIT diagram for both randomisation arms and intervention arm sub-groups

### Translational studies

The molecular determinants of response to CRT in rectal cancer are currently poorly understood. The TRIGGER trial represents a unique opportunity to undertake comprehensive ‘genotype-phenotype’ comparisons as this will enable validation studies for candidate biomarkers from preliminary studies [34]. Patients will be asked to consent to the transfer of their tumour and normal mucosal tissue from the pre-treatment biopsy and post-treatment resection specimen to the central laboratory (Institute of Cancer Research/Royal Marsden BRC, UK), and the collection of an additional research blood samples. Slides will be assessed for pathological tumour regression and tumour cell density centrally (Leeds Institute of Cancer and Pathology, UK) and tissue will also be assessed in collaborating centres (Radboud University Nijmegen, Netherlands and University of Southampton, UK).

### Patient-reported outcome measures (PROMs)

The QoL aspect will be evaluated through validated self-administered PROMs questionnaires at 12 and 36 months post completion of CRT. All patients will be requested to fill out a European Organisation for Research and Treatment of Cancer Quality of Life Questionnaire-core 30, v3 (EORTC QLQ-C30) [35] and the EuroQol Group five dimensions Health Questionnaire (EQ-5D) [36] to derive a global health-related QoL assessment [37]. In addition to this, bowel function will be assessed through the Low Anterior Resection Syndrome (LARS) questionnaire and will be reported according to the severity of bowel dysfunction, as has been reported previously [38].

## Withdrawals

By consenting to the trial, patients will understand that they are consenting to follow-up, data collection, additional MRI scans, additional endoscopy and the collection of biological samples for future research. Selective agreement to these investigations will be treated as a partial withdrawal. Patients have the right to withdraw partially or fully from the study at any time and for any reason without prejudice to their future medical care. Patients’ decision for full withdrawal must be recorded in hospital records and a *withdrawal* CRF should be completed, including primary reason for withdrawal. No further CRFs, except for serious adverse event data, should be completed. Partial withdrawal of consent means that the patient does not wish any further trial treatment but is still willing to provide on-going observational data by continuing with study follow-up. A patient may discontinue trial treatment whenever the treatment is no longer in the patient’s best interests, the reasons for doing so should be clearly recorded.

## Safety evaluation and reporting of adverse events

The investigator is responsible for ensuring that all adverse events, adverse reactions, unexpected adverse reactions (UARs), suspected unexpected serious adverse reactions (SUSARs), and serious adverse events (SAEs) observed by the investigator or reported by patients from the date of the post-CRT MRI until 30 days after the last dose of chemotherapy is administered are properly captured in the patients’ medical records. Adverse events occurring in all arms of the trial should be reported with the same diligence so that bias is not introduced to the apparent incidence of adverse events observed in either arm. The Trial Management Group (TMG) will review all events and reactions. All SAEs will be reported to the TMG approximately 6-monthly and to the Data Monitoring and Ethics Committee (DMEC) annually.

## Discussion

The current consensus is to plan treatment for rectal cancer using baseline MRI staging [7]. However, this position is changing and some authors believe that there is a moral imperative to inform patients if they have had a cCR following CRT, with a view to offering these patients deferral of surgery [39, 40]. With the accurate detection and surveillance of a cCR still problematic, there is a need to develop validated and reproducible means of assessing response to CRT in rectal cancer in a trial setting. The TRIGGER trial is designed to prospectively evaluate mrTRG as a novel biomarker for assessing CRT response. It is the first randomised control trial in rectal cancer to stratify management according to the grade of response to pre-operative CRT, and thus tailor treatment to achieve optimal oncological and functional outcomes. Initially, the feasibility study will assess the ability to recruit and randomise patients to deliver a mrTRG-directed management. If this is successful, we intend to perform a phase III trial that will assess whether mrTRG can improve QoL and DFS through a personalised dual management approach; ‘poor responders’ will be offered additional treatment to enable further downstaging and early treatment of systemic relapse risk, and ‘good responders’ will be offered deferral of surgery, potentially avoiding the morbidity and mortality of surgery.

## Trial status

The TRIGGER trial is open in the feasibility phase, and at the time of submission 20 patients from three centres have been recruited.

## Additional files

Additional file 1: Trial Protocol Version 5.0. (PDF 2102 kb)

Additional file 2: REGISTRATION Patient Information Sheet Version 1.1. (PDF 180 kb)

Additional file 3: RANDOMISATION Patient Information Sheet Version 4.0. (PDF 410 kb)

Additional file 4: Completed SPIRIT Guideline Checklist for TRIGGER. (DOC 123 kb)

## Abbreviations

AR: Adverse reactions
CAPOX: Combination chemotherapy treatment with capecitabine with oxaliplatin
cCR: Clinical complete response
CFS: Colostomy-free survival
CRF: Clinical Report Form
CRM: Circumferential resection margin
CRT: Pre-operative chemoradiotherapy
DFS: Disease-free survival
DMEC: Data Monitoring and Ethics Committee
DR: Distant recurrence
EORTC QLQ-C30: European Organisation for Research and Treatment of Cancer Quality of Life Questionnaire-Core 30, v3
EQ-5D: EuroQol Group five dimensions Health Questionnaire
FOLFOX: Combination chemotherapy treatment with folinic acid, fluorouracil and oxaliplatin modified de Gramont
LARS: Low Anterior Resection Syndrome
MDT: Multidisciplinary team
MHRA: Medicines and Healthcare products Regulatory Agency
MREC: Multicentre Ethical Committee
mrEMVI: MRI-assessed evidence of extramural venous invasion
mrTRG: MRI-assessed tumour regression grade
OS: Overall survival
pCR: Pathological complete response
PROM: Patient-reported outcome measure
QoL: Quality of life
SAE: Serious adverse event
SUSAR: Suspected unexpected serious adverse reaction
TRIGGER: Magnetic resonance tumour regression grade as a novel biomarker to stratify management of good and poor responders to chemoradiotherapy: a rectal cancer multicentre randomised control trial
UAR: Unexpected adverse reaction
