# Supplementary material for: A rectal cancer feasibility study with an embedded phase III trial design assessing magnetic resonance tumour regression grade (mrTRG) as a novel biomarker to stratify management by good and poor response to chemoradiotherapy (TRIGGER): study protocol for a randomised controlled trial
Source: Trials. 2017 Aug 29;18:394. doi: 10.1186/s13063-017-2085-2 (PMC5576102; doi:10.1186/s13063-017-2085-2)
Supplement: Supplementary file 1 — Trial Protocol Version 5.0. (PDF 2102 kb) [file 13063_2017_2085_MOESM1_ESM.pdf]

UK NIHR Portfolio Trial, pump-primed funding from The Pelican Cancer Foundation

Magnetic Resonance Tumour Regression Grade (mrTRG) as a Novel Biomarker to Stratify Management of Good And Poor Responders to Chemoradiotherapy: A Rectal Cancer Multicentre Randomised Control Trial

The TRIGGER Feasibility Trial

Protocol Version: 5.0

Protocol Date: 7 March 2017

IRAS Number: 156408

EudraCT Number: 2015-003009-40

REC Number: 15/LO/1836

Sponsors Number: CCR 4326

SIGNATURE PAGE

The undersigned confirm that the following protocol has been agreed and accepted and that the Chief Investigator agrees to conduct the trial in compliance with the approved protocol and will adhere to the principles outlined in the Medicines for Human Use (Clinical Trials) Regulations 2004 (SI 2004/1031), amended regulations (SI 2006/1928) and any subsequent amendments of the clinical trial regulations, GCP guidelines, the Sponsor's SOPs, and other regulatory requirements as amended.

I agree to ensure that the confidential information contained in this document will not be used for any other purpose other than the evaluation or conduct of the clinical investigation without the prior written consent of the Sponsor

I also confirm that I will make the findings of the study publically available through publication or other dissemination tools without any unnecessary delay and that an honest accurate and transparent account of the study will be given; and that any discrepancies from the study as planned in this protocol will be explained.

Chief Investigator

Signature: Date: 7 March 2017

Name: (please print): GINA BROWN

Statistician

Signature: Date: 7 March 2017

Name (please print): KAREN THOMAS

Principal Investigator's Agreement

I have read the attached protocol entitled "Magnetic Resonance Tumour Regression Grade (mrTRG) as a Novel Biomarker to Stratify Management of Good and Poor Responders to Chemoradiotherapy: A Rectal Cancer Multicentre Randomised Control Trial" Version 5.0 dated 7 March 2017 and agree to abide by all provisions set forth therein. I agree to comply with the principles of Good Clinical Practice (GCP), the EU and GCP Directives (2001/20/EC; 2005/28/EC) The Medicines for Human Use (Clinical Trials) Regulations 2004 (SI 2004/1031) and any subsequent amendments. I agree to ensure that the confidential information contained in this document will not be used for any purpose other than the evaluation or conduct of the clinical investigation without the prior written consent of the Royal Marsden NHS Foundation Trust.

Principal Investigator

Signature: Date:

Name: (please print):

KEY TRIAL CONTACTS

For general queries and supply of trial materials please contact the Trial Manager at the TRIGGER Trial office listed below. Please direct clinical queries during office hours to the Chief Investigator listed below or an appropriate member of the Trial Management Group listed on next page.

**Chief Investigator**	Name: Professor Gina Brown<br>Email: gina.brown@rmh.nhs.uk<br>Phone: 0208 661 3964
**Royal Marsden Principle Investigator & Pharmacovigilance Lead**	Name: Dr Sheela Rao<br>Email: sheela.rao@rmh.nhs.uk<br>Phone: 0208 642 6011
**Trial Physician**	Name: Dr Shelize Khakoo<br>Email: Shelize.khakoo@rmh.nhs.uk<br>Phone: 0208 642 6011 Ext. 1488
**Trial Manager**<br>**TRIGGER Trial Office**	Name: Dr Michelle Frost<br>Email: michelle.frost@rmh.nhs.uk<br>Phone: 0208 915 6067<br>Fax : 0208 915 6721<br>Address : Department of Radiology, Royal Marsden Hospital, Downs Road, Sutton, Surrey, SM2 5PT
**Trial Research Nurse**	Name: Mr Ceri Evans<br>Email: ceri.evans@rmh.nhs.uk<br>Phone: 0208 642 6011 Ext. 4208
**Sponsor**	**The Royal Marsden NHS Foundation Trust**<br>For any enquiries, please contact the Trial Manager at the TRIGGER Trial Office listed above or email your enquiry to: gcpcomplianceteam@rmh.nhs.uk
**Funder**	Pelican Cancer Foundation – Pump prime grant
**Statistician**	Name: Karen Thomas<br>Email: karen.thomas@rmh.nhs.uk<br>Phone: 0208 661 3641
**Randomisation**	See section 7.4. Fax Consent Form 2 and RANDOMISATION Form to:<br>FAX: 0208 915 6721<br>Enquires: 0208 915 6067
**Serious Adverse Event Reporting Fax Number**	FAX: 0208 915 6762

TRIAL MANAGEMENT GROUP

Investigators

Chief Investigator
Prof Gina Brown
Department of Radiology
Royal Marsden Hospital
Sutton, SM2 5PT
T: 020 8661 3964
F: 020 915 6721
gina.brown@rmh.nhs.uk

Royal Marsden PI & CTIMP lead
Dr Sheela Rao
Department of Medicine
Royal Marsden Hospital
Sutton, SM2 5PT
T : 0208 642 6011
F : 0208 643 9414
sheela.rao@rmh.nhs.uk

Trial Physician
Dr Shelize Khakoo
Gastrointestinal Unit
Royal Marsden Hospital
Sutton, SM2 5PT
T: 0208 642 6011 Ext.1488
E: Shelize.khakoo@rmh.nhs.uk

Clinical Oncologists
Prof David Cunningham
Dept of Medicine
Royal Marsden Hospital
Downs Road
Sutton, SM2 5PT
T: 0208 661 3156
E:david.cunningham@rmh.nhs.uk

Dr Richard Adams
Velindre Cancer Centre
Velindre Hospital
Cardiff, CF4 7XL
Tel: 02920 615888 Ext 6239
richard.Adams@wales.nhs.uk

Dr Diana Tait
Royal Marsden Hospital
Downs Road
Sutton, SM2 5PT
Tel: 020 8661 3370
diana.tait@rmh.nhs.uk

Surgeons
Prof Paris Tekkis
Consultant Colorectal Surgeon
Royal Marsden Hospital
London, SW3 6JJ
T: 0207 808 2195
p.tekkis@imperial.ac.uk

Mr Shahnawaz Rasheed
Consultant Surgeon
Royal Marsden Hospital
Fulham Road, SW3 6JJ
T: 0207 808 2195
Shahnawaz.rasheed@rmh.nhs.uk

Mr Brendan Moran
North Hampshire Hospital
Basingstoke, RG24 9NA
T: 01256 314 709
E: brendan.moran@hhft.nhs.uk

Mr Nick Battersby
Surgical Registrar
Wessex School of Surgery
Winchester
Hampshire, SO21 2RU
T: 07809 463090
E: nickbattersby@nhs.net

Mr Nadar Francis
Consultant Surgeon
Yeovil District Hospital
Higher Kingston
Yeovil
Somerset, BA21 4AT

Surgeon & Lead for Tissue Microarray analysis
Mr Alex Mirnezami
Somers Cancer Research Building,
University of Southampton
Southampton, SO16 6YD
T: 023 8079 5170
E: *ahm@soton.ac.uk*

Central Pathology Review
Prof Phil Quirke
Wellcome Trust Bremner Building
St James's University Hospital
Leeds, LS9 7TF
T: 0113 343 8407
E: P.Quirke@leeds.ac.uk

Dr Nick West
Leeds Institute of Cancer & Pathology
St James's University Hospital
Leeds, LS9 7TF
T: 0113 343 8626/0113 2068343
E: n.p.west@leeds.ac.uk

Translational sub-study
Dr Irene Chong
Division of Molecular Pathology
Institute of Cancer Research
London, SW3 6JB
T: 02071 535 138 irene.chong@icr.ac.uk

Dr Anguraj Sadanandam
Department of Molecular Pathology
Institute of Cancer Research
15 Cotswold Road
Sutton, Surrey, SM2 5NG
T: 0208 915 6631
anguraj.sadanandam@icr.ac.uk

Prof Iris Nagtegaal
Department of Pathology
Radboud University
Nijmegen, Netherlands 6500HB
T: +312 4361 0229
F: +312 4366 8750
Iris.Nagtegaal@radboudumc.nl

Dr Nicola Valerie
Department of Molecular Pathology
Institute of Cancer Research
15 Cotswold Road
Sutton, Surrey, SM2 5NG
T: 0208 722 4283
Nicola.valerie@icr.ac.uk

Trial Staff and patient representative

Operational Management
Dr Michelle Frost
Trial Manager
Royal Marsden Hospital
T: 0208 915 6067
E: michelle.frost@rmh.nhs.uk

Patient Representative
Mr Matthew Walker

Trial Research Nurse
Mr Ceri Evans
Research Nurse
Royal Marsden Hospital
T: 0208 642 6011 Ext. 4280
E: ceri.evans@rmh.nhs.uk

Statistician
Ms Karen Thomas,
Statistics Unit, R&D
Royal Marsden Hospital
Sutton, SM2 5PT
T: 0208 661 3641
E: Karen.Thomas@rmh.nhs.uk

TRIAL SUMMARY:

**Trial Title**	Magnetic Resonance Tumour Regression Grade (mrTRG) as a Novel Biomarker to Stratify Management of Good And Poor Responders to Chemoradiotherapy: A Rectal Cancer Multicentre Randomised Control Trial - The TRIGGER Feasibility Trial
**Short Title**	TRIGGER Feasibility Trial
**Public Title**	A trial looking at a magnetic resonance imaging biomarker to determine treatment after chemotherapy and radiotherapy in patients with cancer of the rectum
**Trial Type & Phase**	Multicentre, Open, Interventional, Randomised Control Trial<br>Phase: Feasibility Study with embedded Phase III design.
**Planned sample size**	90
**Recruitment**	UK based trial with Centres in Europe, North America and Brazil
**Brief Rationale**	Most MRI staged, locally advanced tumours receive chemoradiotherapy (CRT). Patients receive an MRI at diagnosis and a second MRI after CRT. Previously there was no reliable method for assessing the treatment response therefore the current standard of care is to plan surgery using the baseline MRI. Recently an MRI based tumour regression grade (mrTRG) has been shown to stratify between tumours that respond well and those that respond poorly to CRT.<br>This protocol is for a feasibility trial that is designed to test whether it is possible to recruit patients and stratify treatment using mrTRG directed management. The 'good responders' (mrTRG1&2) often have no evidence of tumour and it may be possible to avoid surgery in this group. The 'poor responders' (mrTRG3-5) are at high risk of poor oncological outcomes and additional therapy before surgery may improve their prognosis.<br>This study will test whether recruitment rates are sufficiently high to allow adequate recruitment in a phase III trial.
**Intervention:**	mrTRG: magnetic resonance tumour regression grade.
**Intervention Description:**	The radiologist must be trained in order to reliably report the mrTRG grade but the MRIs are performed routinely, no change from the MERCURY MRI protocol is required.<br>*Control Arm*: Management according to national guidelines - conventional MDT, clinical assessment post-treatment planning using the baseline MRI.<br>*Intervention Arm*: mrTRG directed management<br>• 'good response' (mrTRG 1&2) – deferral of surgery recommended.<br>• 'poor response' (mrTRG 3-5) – additional systemic chemotherapy prior to surgery
**Summarised Inclusion Criteria**	Biopsy confirmed adenocarcioma 0-15cm from anal verge (radiologically or endoscopically)<br>MRI defined locally advanced rectal carcinoma<br>Be deemed to require chemoradiotherapy<br>Scheduled to receive 45 Gy-55Gy long course radiotherapy (CRT)
**Summarised Exclusion Criteria**	Metastatic disease<br>MRI, CRT and/or chemotherapy contraindications<br>A post-treatment MRI performed more than 10 weeks after the completion of CRT<br>Patient scheduled to receive less than 45Gy or more than 55Gy long course radiotherapy<br>Previous malignancy within preceding 5 years (with exceptions)

**Planned feasibility trial period**	Feasibility trial patient recruitment period March 2016 – June 2018<br>Clinical follow-up duration of 5 years
**Primary Objective**	Assess the feasibility of a phase III trial by assessing the patient recruitment rate
**Secondary Objectives**	1. Assess response rate by comparing the reported mrTRG in the control and intervention arm.<br><br>2. Evaluate the reproducibility of mrTRG by assessing the strength of agreement of the reported mrTRG between the site radiologists and central radiologist.<br><br>3. To evaluate safety by assessing acute drug toxicity and 30 day surgical morbidity.<br><br>4. Compare the pCRM involvement rate in the control versus intervention arm<br><br>5. Compare the quality of surgery in the control and intervention arms, this will include patients who deferred surgery but later develop tumour regrowth<br><br>6. Assess treatment plan concordance and completion rates
**Exploratory objectives**	To assess the correlation between mrTRG and pathological TRG and the correlation between mrTRG and proliferative, apoptotic and genomic markers

TRIAL FLOWCHART

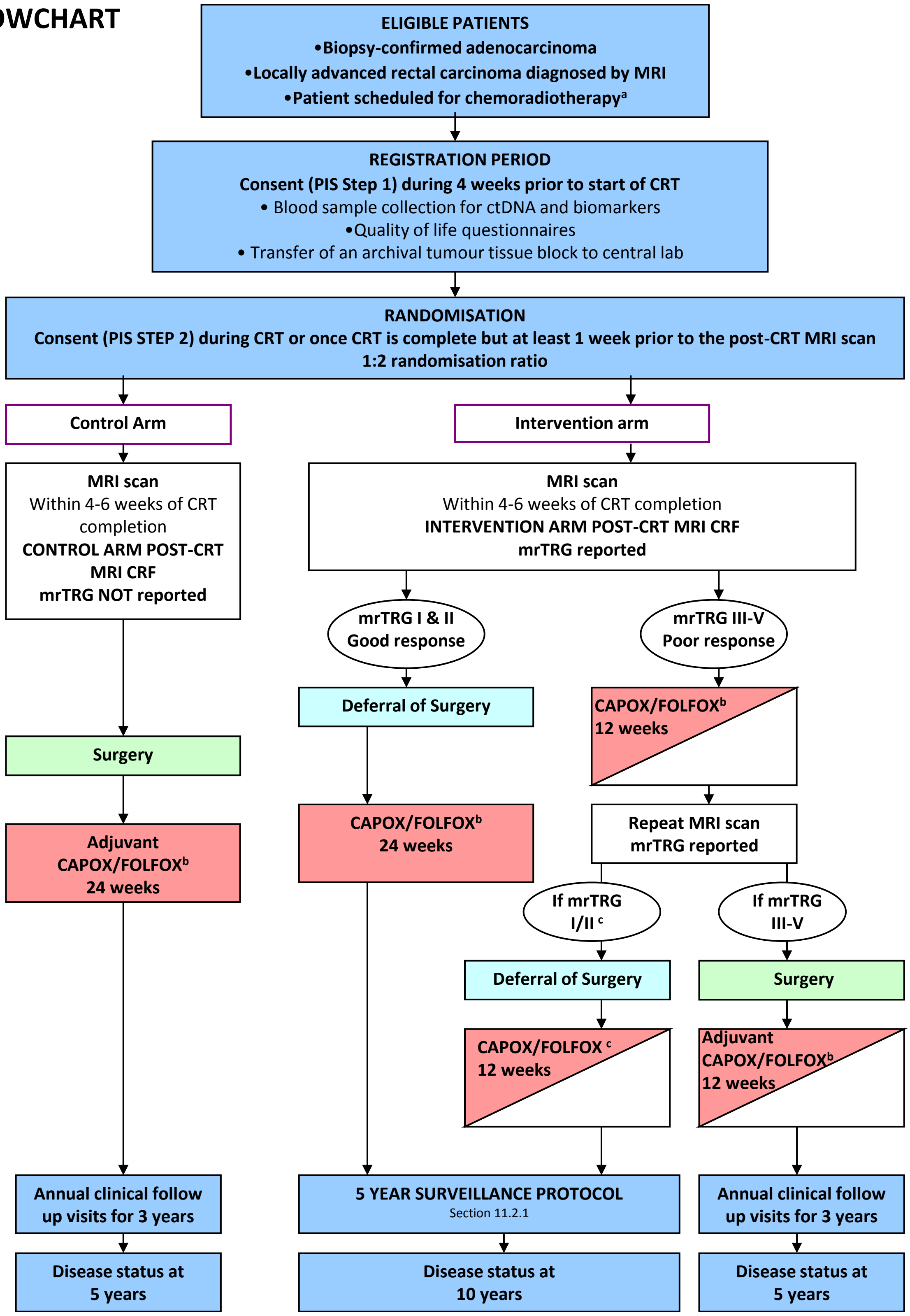

[a] Scheduled to receive 45Gy-55Gy long course radiotherapy (inclusion criterion 4). [b] Treatment decision should be made prior to registration (planned choice is a randomisation stratification variable). Medical oncologist may choose to use CAPOX or FOLFOX, or single agent capecitabine or 5-FU if concomitant use of oxaliplatin is contraindicated. [c] If patient defers surgery then the remaining 12 weeks of chemotherapy should be given as soon as possible following the repeat MRI scan and MDT meeting.

CONTENTS

SIGNATURE PAGE ii
KEY TRIAL CONTACTS..... iii
TRIAL MANAGEMENT GROUP iv
Trial summary: v
1. Introduction 1
2. Literature Review 1
2.1 The good response group (mrTRG 1-2).....1
2.2 the Poor response group (mrTRG 3-5).....2
2.3 The need for the TRIGGER trial 2
3. Trial Design 3
3.1 TRIAL OVERVIEW 3
3.2 Randomization Method 3
3.3 RESTAGING MRI REPORTING AND INTERVENTION ARM SUB-GROUPS 3
4. FEASIBILITY trial Objectives and End Points 4
4.1 Primary objective of the feasibility Trial.....4
4.2 Secondary objectives of the feasibility TRIAL 5
4.3 Exploratory objectives 6
4.4 TRIAL Endpoints.....6
4.4.1 Primary Endpoint 6
4.4.2 Secondary endpoints 6
4.5. Objectives of Main Phase iii Trial 6
5. SITE REQUIREMENTS AND ELIGIBILITY..... 7
5.1 study setting.....7
5.2 Site requirements and Eligibility.....7
5.2.1 Radiological Staging 8
5.3 Required site training.....8
5.4 SITE ACTIVATION 8
5.5 The Delegation Log 9
6. Patient Eligibility criteria..... 9
6.1 Inclusion Criteria.....9
6.2 Exclusion Criteria 9
7. Registration and Randomisation 10
7.1 Informed Consent 10
7.1.1 Patient Information Sheet 1 – Registration 10
7.1.2 Patient Information Sheet 2 – Randomisation 11
7.1.3 Screening, Registration and Randomisation logs..... 11
7.2 PATIENT ID NUMBER.....12
7.3 Registration procedure 12
7.3.1 Histopathology information and Clinical Investigations Required at Registration..... 13
7.4 RANDOMISATION PROCEDURE.....13
7.4.1 Acting on the Randomisation..... 14
7.4.2 Registered patients who do not consent to randomisation 14
7.5 Withdrawal CRITERIA.....15
7.5.1 Non compliance with the protocol 15
7.5.2 Discontinuation of trial treatment..... 15
7.5.3 Losses to follow-up 16
8. Summarised Patient Pathways..... 17
8.1 THE CONTROL ARM – BEST CURRENT PRACTICE 18
8.2 INTERVENTION ARM – A 'GOOD RESPONSE' TO CHEMORADIOTHERAPY (mrTRG 1 & 2) 19

8.2.1 PROCEDURE IF A PATIENT DECLINES DEFERRAL OF SURGERY 20
8.2.2 PROCEDURE IF A LOCAL REGROWTH IS DETECTED 20
8.2.3 Suspected regrowth pathway for patients who defer surgery 21
8.3 EXPERIMENTAL ARM – A 'POOR RESPONSE' TO CHEMORADIOTHERAPY 22
9. TRIAL Treatment 23
9.1 Treatment during Chemoradiotherapy 23
9.2 TRIAL INVESTIGATIONAL MEDICINAL PRODUCTS 24
9.3 DISPENSING AND DRUG SUPPLIES 24
9.4 Summary of product characteristics 24
10. Assessment Schedules 25
10.1 Assessment schedule summary: control arm 26
10.2 intervention Arm - 'good response' to chemoradiotherapy 28
10.3 Intervention Arm -'poor response' to chemoradiotherapy 30
10.4 biomarker development 32
10.4.1 Blood samples for biomarker analysis 32
10.4.2 H&E STAINED GLASS SLIDES 33
10.4.3 paraffin-embedded diagnostic tumour tissue 33
10.4.4 SAMPLE PROCESSING AT ROYAL MARSDEN 33
10.4.5 Sample labelling, storage and destruction 33
11. Clinical Follow-up 34
11.1 Post-Surgery Follow-up 35
11.2 Deferral of Surgery Follow-up 36
11.2.1 Surveillance schedule for patients who defer surgery 38
11.3 Definitions Required For Follow-up 39
11.4 Compliance with the Protocol 40
11.5 Definition of End of Trial 40
12 DATA MANAGEMENT 40
12.1 COMPLETING CASE REPORT FORMS 40
12.2 CORRECTIONS TO CASE REPORT FORMS 41
12.3 MISSING DATA 41
12.4 DATA QUERIES 41
12.5 SUBMISSION TIMELINES 41
12.6 DATA COLLECTION AND RETENTION 41
12.7 TRIAL DATABASE 42
13. TRIAL MONITORING AND OVERSIGHT 42
13.1 SELF-ASSESSMENT MONITORING 42
13.2 CENTRAL MONITORING 43
13.3 NON-COMPLIANCE/'FOR CAUSE' ON-SITE MONITORING 43
13.4 OVERSIGHT COMMITTEES 44
13.4.1 Trial Management Group (TMG) 44
13.4.2 Independent Data Monitoring and Ethics Committee (DMEC) 44
13.4.3 ROLE OF THE ROYAL MARSDEN NHS FOUNDATION TRUST 45
14. Statistical Analysis and Sample Size 45
14.1 Sample Size 45
14.1.1 Phase III Study Sample Size 45
14.1.2 Feasibility Study Sample Size 45
14. 2 Analysis methods 46
14.2.1 Primary Endpoint 46
14.2.2 Secondary Endpoints 47
15. Organisation 48
15.1 Funding 48

15.2 Principal Investigator at each Centre 49
15.3 Central Coordination 49
15.4 Clinical Queries 50
15.5 Publications 50
16. Research Governance 50
Sponsor 50
Clinical Trials Unit 50
Confidentiality of Personal Data 50
Long-Term Storage of Data 51
Indemnity 51
17. Abbreviations and Definitions: 52
18. References 53
19. Appendices 55
Appendix 1: Safety Monitoring Procedures 56
1. Definitions 56
1.1. Adverse Events 56
1.2 Adverse Reaction (AR) 56
1.3 Unexpected Adverse Reaction (UAR) 56
1.4 Serious Adverse Event (SAE) 56
1.4.1 Disease-related Events 57
1.4.2 Important Medical Events 57
1.5 Serious Adverse Reaction (SAR) 57
1.6 Suspected Unexpected Serious Adverse Reaction (SUSAR) 57
2 Reporting Procedures for ALL Adverse Events 57
2.1 Defining the adverse event 58
2.1.1 Determining adverse event severity 58
2.1.2 Determining adverse event seriousness 59
2.1.3 Determining adverse event causality 59
2.1.4 Adverse Events of Special Interest 59
2.2 SERIOUS ADVERSE EVENT REPORTING PROCEDURES 59
2.2.1 Exemptions from SAE Report Submission 60
2.3 Determining adverse event expectedness 60
2.4 Notifications 62
2.5 SAE Reporting Flowchart 63
2.6 Serious breaches and urgent safety measures 64
2.7 Contraception and Pregnancy Reporting 64
Appendix 2: Chemotherapy 66
1 OVERVIEW AND TIMING OF SYSTEMIC CHEMOTHERAPY 66
1.1 CHEMOTHERAPY TREATMENT POST-SURGERY 67
1.2 CHEMOTHERAPY TREATMENT IN INTERVENTION ARM – GOOD RESPONDERS 67
1.3 CHEMOTHERAPY TREATMENT IN INTERVENTION ARM – POOR RESPONDERS 67
1.4 Changes to regimen choice during treatment 67
2 RECOMMENDED CAPOX Regimen (3 weekly cycle) 67
3 RECOMMENDED CAPECITABINE MONOTHERAPY REGIMEN (3 weekly cycle) 68
4 RECOMMENDED FOLFOX REGIMEN (2 weekly cycle) 68
5 RECOMMENDED 5-FU MONOTHERAPY REGIMEN (2 weekly cycle) 69
6 Toxicity and Dose Modifications 70
6.1 Toxicity and dose modification NOTES 70
6.2 dose modifications 70
6.3 CAPOX/CAPECITABINE MONOTHERAPY 70
6.3.1 Haematological Toxicity: 70

6.3.2 Non-haematological toxicities: ... 71
6.3.2.1 Capecitabine non-haematological toxicity: ... 71
7 CONCOMITANT MEDICATIONS ... 73
8 DOSE BANDING ... 74
Appendix 3: Radiotherapy ... 76
1 Pre-Operative Chemo/Radiotherapy ... 76
Patient set up ... 76
1.1 Target Volumes ... 76
1.1.1 Definition of the Planning Target Volume for phase II ... 76
1.1.2 Definition of the Planning Target Volume: Phase I ... 76
1.1.3 CTV Inguinal nodes (for very low tumours involving anus) ... 77
1.2 Dose ... 77
Appendix 4: Surgery and Surgical Morbidity ... 78
Surgery ... 78
Surgical Morbidity ... 78
Appendix 5: Histopathology ... 80
Histopathological evaluation of the resection specimen ... 80
Appendix 6: MRI Protocol ... 96
MRI & Local Rectal Cancer Staging ... 97
Assessing Response to Treatment ... 99
APPENDIX 7: translational biomarker substudy ... 103
1. Rationale ... 103
2. Study aim and objectives ... 103
3. Study Design and methodology ... 104
4. Study endpoints ... 105
5. Data analysis ... 105
6. Statistical considerations ... 106
Appendix 8: Protocol Amendment History ... 107

1. INTRODUCTION

Currently 45-55% of rectal cancer patients receive preoperative chemoradiotherapy for high-risk disease. Pre-operative chemotherapy and radiotherapy are intended to downstage the tumour, this has three potential benefits: an increased likelihood of a clear circumferential resection margin (CRM); less radical surgery (in the MERCURYII low rectal cancer study 35% of the good responders underwent a change in the plane to less radical surgery); and, to allow deferral of surgery where feasible. These benefits would be expected to result in an improved prognosis. However, the response to treatment is highly variable; up to 30% of patients will achieve a complete or near complete response, and the remainder do not respond sufficiently to survive the disease to the same extent. A poor pathological tumour regression grade (pTRG) is associated with significantly higher local recurrence, distant metastatic rates (the principal cause of poor survival) with lower overall survival compared with good pTRG.[1,2]

Although the need for a validated means of pre-operative assessing response to treatment is widely accepted,[3] there has been no reliable method of assessing this response pre-operatively and therefore the current standard of care advocates using the baseline MRI as the reference for the plane of surgery regardless of any assessment of treatment response.[4] Recently, an MRI based tumour regression grade has been developed (mrTRG).[5] Patients with a poor CRT response (mrTRG4&5) have a 5-year overall survival of 27% versus 72% (*p*=0.001) for a good CRT response (mrTRG1-3).[6] This novel imaging biomarker has been reliable and reproducible between multiple independent radiologists.[6,7] These findings are encouraging, as it appears that the subgroup of mrTRG 1-2 have a significant chance of complete long-term tumour regression. Although validation of this as a biomarker has been based on prospective evaluation of MRI scans against both pathology and survival outcomes, there is insufficient evidence that this information can be used to alter current treatment decisions.

2. LITERATURE REVIEW

2.1 THE GOOD RESPONSE GROUP (MRTRG 1-2)

The mortality rate from a major bowel resection is up to 5%, furthermore major morbidity is common and long-term functional impairment is likely.[8-10] For many patients it is difficult to accept such risks when there is no apparent residual cancer (clinical complete response, cCR).[11] A series of publications by Angelita Habr-Gama have demonstrated that it is feasible and safe to avoid radical surgery by closely observing patients that have a clinical complete response after chemoradiotherapy[12-14], Deferral of surgery is now being offered in a number of centres using clinical complete assessment (endoscopy and digital rectal examination).[15-17] However current techniques for assessing deferral of surgery are unreliable and up to two thirds of patients with a complete response (pCR) are not identified pre-operatively.[18-21] The current consensus is to plan treatment using baseline staging,[4] however this position is changing, and some authors

believe there is a moral imperative to inform patients if their tumour has evidence of a clinical or radiological complete response, with a view to offering these patients deferral of surgery.[22,23] By using mrTRG prospectively in a randomized controlled study, it may be possible to determine the precise safety of this approach whilst increasing the proportion of complete responders that are identified using an objective and validated tool. By avoiding radical surgery, this group of rectal cancer patients may have a reduced overall morbidity and mortality as well as an improvement in quality of life.

2.2 THE POOR RESPONSE GROUP (MRTRG 3-5)

Intensifying pre-operative therapy may cause additional toxicity. The TRIGGER trial will offer poor responders consolidation therapy using a fluorouracil (5-FU) based regimen combined with Oxaliplatin (Infusional 5-FU or Capecitabine with Oxaliplatin, FOLFOX or CAPOX). These regimens have been shown to be effective systemic treatments for colorectal malignancy and they are recommended by most international guidelines.[24,25] Usually these regimens are used post-operatively however they have been used safely in the pre-operatively setting. An RCT by Garcia-Aguilar et al found that FOLFOX can be used as pre-operative consolidation therapy along with conventional CRT without evidence of additional toxicity.[26] In the Socrates trial, patients received a similar regimen of radiotherapy and oxaliplatin, however capecitabine (the 5-FU oral prodrug) was used.[27] The CAPOX treatment regime was offered to patients with locally advanced rectal cancer, 82 or the 83 patents that enrolled into the trial completed pre-operative CAPOX regime and 78 patients proceeded to surgery. Similar compliance rates were seen with pre-operative CAPOX-RT in a German study, where compliance rates were 96%.[28] In TRIGGER, 'poor responders' will be offered systemic Oxaliplatin and either 5-FU or capecitabine pre-operatively; this appears to not only improve tumour response rates but also reduce the rates of distant metastatic disease due to the earlier introduction of systemic therapy to a high risk subgroup of patients.[29]

2.3 THE NEED FOR THE TRIGGER TRIAL

This trial is designed to use the mrTRG as a biomarker for response. No prospective trials have stratified rectal cancer management according to the mrTRG or any other biomarker for response to treatment. Initially the feasibility study will assess ability to recruitment patients and to deliver mrTRG directed management. If this is successful we intend to perform a phase III trial that will assess whether mrTRG can improve quality of life and overall survival through a personalised MRI directed dual management approach; 'poor responders' will be offered additional treatment to enable further downstaging and early treatment of systemic relapse risk and 'good responders' will be offered deferral of surgery, potentially avoiding the morbidity and mortality of surgery.

3. TRIAL DESIGN

3.1 TRIAL OVERVIEW

This is a feasibility study intended to establish safety and feasibility of recruitment numbers for the larger phase III study. The feasibility protocol will continue as a phase III study subject to the two conditions of feasibility being met i.e. (1) reaching target recruitment rates of 6/mth during the last 4 months of recruitment and (2) Funding is received for the main Phase III trial. At this point a substantial amendment will be submitted to include the primary endpoint for the Phase III trial of disease-free survival from the point of registration. Recruitment to feasibility will continue until the Phase III component is approved to enable the TMG to report on the secondary endpoints of the feasibility component of the trial. Data from patients in the feasibility study will be included in the final analysis of the phase III study. This is intended to optimize recruitment. It is subject to the trial safety standards and stopping rule. The Royal Marsden will remain as trial sponsor for the duration of the feasibility and Phase III studies.

This is a multicentre, prospective, open, randomised control trial. It is not possible to blind the patient, radiologist, surgeon or pathologist but prospective proforma based reporting by the radiologists and pathologists will increase the objectivity of data recorded.

3.2 RANDOMIZATION METHOD

Randomization will be in a 2:1 ratio in favour of the mrTRG directed management arm. Randomization will be stratified to avoid differences between arms in important prognostic variables. Stratification variables include:

- recruiting site
- extramural venous invasion on MRI (mrEMVI grades ≤2 versus >2)
- tumour height (on MRI, height of tumour from anal verge ≤ 6 cm versus > 6 cm)
- the planned choice of systemic chemotherapy (CAPOX/FOLFOX, versus single agent 5-FU/capecitabine)

3.3 RESTAGING MRI REPORTING AND INTERVENTION ARM SUB-GROUPS

Patients should undergo the restaging MRI 4-6 weeks (maximum of 10 weeks) following completion of their initial pre-operative CRT therapy with planned surgery in the control arm at 6-12 weeks and commencement of further consolidation therapy in the interventional arm within 12 weeks.

The radiologist will be trained to report the MRIs according to synoptic proforma reporting with specified templates:

CONTROL ARM POST-TREATMENT MRI CRF - there is currently a lack of surgical equipoise to change operative approach based on post treatment MRI assessment. Therefore the operative decision in the control arm will be determined by the baseline MRI. Post-operative adjuvant therapy will be offered to patients based on baseline MRI features of high risk.

INTERVENTION ARM POST-TREATMENT MRI CRF – synoptic reporting will be performed according to the MRI form. The MRI form for the intervention arm has an additional datafield for mrTRG reporting. The mrTRG result should be discussed at the MDT:

For mrTRG 1&2 (good response) - deferral of surgery should be offered to the patient, with chemotherapy offered if high risk features were present on the baseline scans.

For mrTRG 3-5 (poor response) – 12 weeks of consolidation therapy will be offered, followed by a restaging MRI prior to a decision regarding surgery.

4. FEASIBILITY TRIAL OBJECTIVES AND END POINTS

The feasibility trial is intended to inform design of a phase III trial. We need to determine the feasibility and safety of deferring surgery, and for poor responders, the acceptability of offering additional pre-operative chemotherapy.

4.1 PRIMARY OBJECTIVE OF THE FEASIBILITY TRIAL

Assess the feasibility of a phase III trial by assessing the patient recruitment rate.

Patient Recruitment

We anticipate that the majority of patients eligible for TRIGGER will be identified at the multidisciplinary cancer meeting - where rectal cancer is initially identified and the decision to offer chemoradiotherapy is made. The trial design is complex and it may be difficult for patients to understand the concept of deferral of surgery. The prospect of further systemic chemotherapy after CRT may be daunting. Finally, being in the control arm and missing out on additional treatment options may cause anxiety for patients. These concerns may limit the number of patients willing to recruit to study and so objective figures are needed to determine if a larger phase III trial is plausible.

Centre Recruitment

This will assess the acceptability of the trial concept and the protocol. Many surgeons have expressed an interest in the trial because they are keen to be able to offer deferral of surgery in a standardised and controlled way. For the oncologists, offering additional preoperative treatment is also of interest. However, patients treated with deferral of surgery undergo more intensive follow-up than the current standard of

care, particularly in the first two years (Section 11.2.1). This may slightly increase the demands on surgical clinics, endoscopy and radiology services. We will record the number of units that open to recruitment.

4.2 SECONDARY OBJECTIVES OF THE FEASIBILITY TRIAL

1. **Assess response rate by comparing the reported mrTRG in the control and intervention arm**

Trial participants are randomised with stratification for the key factors that effect response (tumour height, mrEMVI and chemotherapy). Therefore the mrTRG should be similar in the control and intervention arm. In order to avoid the MDT being informed of the mrTRG in the control arm we will report the mrTRG grade in the control arm by centralised assessment. Comparison between the mrTRG in the control versus intervention arm will allow us to evaluate the amount of additional response achieved by using 'upfront' systemic chemotherapy.

2. **Evaluate the reproducibility of mrTRG by assessing the strength of the agreement between the recruiting radiologists and central radiologist**

A prerequisite to opening a site is that the allocated radiologist is able to perform MRIs according to the published protocol and achieve a high Kappa agreement (k≥0.7) with a training dataset that includes mrTRG grades and radiological evidence of regrowth. The central MRI review will also be used to identify any missed 'radiological regrowths' using the MRI training dataset. The agreement between the recruiting radiologists and central radiologist scored mrTRG grades for scans acquired during the study for patients randomised to the intervention arm will also be assessed retrospectively at the end of the feasibility trial.

3. **To evaluate safety by assessing acute drug toxicity and 30 day surgical morbidity**

The acute drug toxicity and the surgical morbidity will be reported descriptively and the adverse events will be discussed by the DMEC.

4. **Compare the pCRM involvement rate in the control versus intervention arm**

The overall pCRM involvement rate between the control and intervention arms will be assessed according to an intention to treat analysis. This will be important for assessing the safety of the mrTRG directed approach. We hypothesise that a greater proportion of downstaging in the intervention arm will result in relative reduction in the overall rate of pCRM involvement compared with the control arm. The rate of inoperable regrowth occurring in patients that are managed by deferral of surgery will be specifically considered.

5. **Compare the quality of surgery in the control and intervention arms, this will include patients who deferred surgery but later develop tumour regrowth and subsequently undergo surgery.**
6. **Treatment plan concordance and completion rates**

To inform the sample size calculation for the main study information regarding the concordance and compliance with the treatment plan discussed at the each patient's MDT meeting prior to CRT will be assessed and captured on the CRF.

4.3 EXPLORATORY OBJECTIVES

Exploratory objectives include assessing the correlation between mrTRG and pathological TRG and the correlation between mrTRG and proliferative, apoptotic and genomic markers. This may help to identify the biological and genetic characteristics of chemosensitivity / chemoresistance tumours and determine whether a better pathology grading system could be developed which may need to incorporate apoptosis and proliferative indices.

4.4 TRIAL ENDPOINTS

4.4.1 PRIMARY ENDPOINT

The total number of patients randomised per month during the last 4 months of recruitment.

4.4.2 SECONDARY ENDPOINTS

- The pCRM rate will be compared between the control and investigation arms.
- The strength of agreement of the reported mrTRG will be evaluated between the site radiologist versus the central radiologist at site initiation and at the end of the feasibility trial.
- Acute toxicity will be recorded to CTCAE v4.0 protocol. The grade 3- 5 adverse events will be compared between the control and intervention arms on an intention to treat basis.
- Early (30 day) and late (up to 12 months) surgical morbidity will be reported according to the Clavien-Dindo classification
- Quality of surgery will be determined by using the mesorectal grading system and a similar system will be used for grading the quality of APE specimens (Pathology appendix).[30,31] Outcomes will be compared between the control and intervention arms on an intention to treat basis.

4.5. OBJECTIVES OF MAIN PHASE III TRIAL

Should feasibility be confirmed the ***primary objective*** **of the Phase III trial** will be to evaluate 3-year disease free survival in patients with locally advanced rectal cancer by using mrTRG directed management to selectively offer surgery and additional pre-operative chemotherapy. There will several important secondary endpoints including an evaluation of the effect that mrTRG directed management has on quality of life.

5. SITE REQUIREMENTS AND ELIGIBILITY

5.1 STUDY SETTING

This is a multicentre trial with an anticipated 10-15 sites being involved in the feasibility stage of the trial. It is also anticipated that a limited number of these sites will be based outside the UK.

5.2 SITE REQUIREMENTS AND ELIGIBILITY

For a site to be identified as eligible for participation the TRIGGER Trial they will be required to:

- Complete a Site Feasibility Form, Contacts Log and Delegation Log for assessment by trial staff at the TRIGGER Trial Office.
- Be able to meet the research and development requirements of their institution.
- Be able to demonstrate a potential for recruiting suitable subjects – sites will be asked to provide details about the target population at their site, including the number of potentially eligible patients the investigator treats.
- Ensure that each patient recruited is discussed at a multidisciplinary team meeting – that includes a minimum of 1 oncologist, 1 pathologist, 1 radiologist and 1 surgeon.
- Have a nominated appropriately qualified individual responsible for pharmacovigilance. In most cases this will be the local Principal Investigator, however pharmacovigilance can be delegated to a medical oncologist if the site PI is a surgeon. This should be documented on the site Delegation Log.
- Agree that the nominated GI Radiologist will complete an MRI Reporting workshop/webinar detailed in Section 5.2.1.
- Comply with the MRI and Pathology proformas.
- Accommodate and be able to deliver all aspects of the protocol, including patient follow-up, particularly the follow-up requirements for safe deferral of surgery.
- Provide contact details for all trial personnel at the participating site. Names and addresses of all participating principle investigators is a requirement of MHRA approval so it is vital this information is received by the trial staff at the Royal Marsden. The trial office must be notified of any changes to trial personnel and/or their responsibilities during the trial. An up-to-date copy of the delegation log can be faxed to the trial office for this purpose and the up-to-date log stored in the trial investigator site file.

- Once the Site Feasibility Form, Contacts Log and Delegation Log are received and assessed at the TRIGGER Trial office, the participation site must apply for their local R&D approval. At the same time the following documents will be required prior to site initiation:
    - A signed Clinical Trial Agreement between the Royal Marsden NHS Foundation Trust and the site.
    - CV of Principle Investigator and co-investigators and evidence of GCP training (or confirmation these are held on site).

5.2.1 RADIOLOGICAL STAGING

The ability to reliably assess the degree of tumour regression on MRI (mrTRG) is fundamental to the success of the trial. To ensure consistency, a nominated study GI radiologist will be asked to participate in a CME-accredited trial-specific MRI reporting workshop/webinar. A site will not be able to open until the allocated radiologist(s) has achieved mrTRG competency (mrTRG kappa ≥ 0.7). The consultant radiologist can book a place on the MRI reporting workshop/webinar by contacting the TRIGGER Trial Office:

Michelle Frost, Trial Manager, Academic Department of Radiology,

Royal Marsden Hospital, Downs Road, Sutton, Surrey, SM2 5PT

Email: michelle.frost@rmh.nhs.uk Tel: 0208 915 6067 Fax: 0208 915 6721

5.3 REQUIRED SITE TRAINING

Prior to a site opening to recruitment, each site will be required to have at a minimum a site PI, one research nurse or trial coordinator and the nominated GI radiologist to participate in TRIGGER trial training. This training may be incorporated at a site initiation visit or participation in a teleconference. It is the responsibility of those who attend the training to disseminate the training to other site personnel.

5.4 SITE ACTIVATION

Following receipt of the documents listed in section 5.2 above and all required training is performed, written confirmation of site activation will be sent to the PI and all relevant site personnel. All trial-related materials will be provided to the site once they are activated and the site will be assigned a unique trial site code for use on trial-related documentation e.g. CRFs.

Following activation to TRIGGER:

- The site should conduct the trial in compliance with the protocol as agreed by the Sponsor, the MHRA and the REC.

- The PI or delegate should document and explain any deviation from the agreed protocol by completing the **PROTOCOL DEVIATION Form**, and communicate this with the trial team at the Royal Marsden.

5.5 THE DELEGATION LOG

All staff taking part in the trial are required to specify their trial responsibilities in the delegation log. These staff will then be authorised by signature to work on the trial by the PI. The log is important because it allows the PI to retain oversight of the staff working on the trial. The delegation logs may also be used to correctly acknowledge individuals in presentations and publications.

All investigators should sign the delegation log and the site PI must co-sign the delegation log to delegate duties and acknowledge overall responsibility for the trial site. Only an appropriately qualified and trained investigator who has a thorough understanding of the trial may take consent.

6. PATIENT ELIGIBILITY CRITERIA

The TRIGGER trial will recruit patients undergoing pre-operative treatment for locally advanced rectal cancer. Participants will be considered eligible if they fulfil all the inclusion criteria and none of the exclusion criteria as defined below. The Eligibility Checklist included on the **TRIAL REGISTRATION Form** should be completed to confirm eligibility and signed by the investigator.

6.1 INCLUSION CRITERIA

Patients must fulfil **<u>ALL</u>** of the following inclusion criteria to be considered eligible for the TRIGGER Trial:

1) Have a biopsy-confirmed adenocarcinoma 0-15cm from the anal verge (on MRI or rigid sigmoidoscopy).
2) Have locally Advanced Rectal Carcinoma diagnosed by MRI (mrCRM unsafe or ≥mrT3c [>5mm beyond muscularis propria] or mrEMVI positive disease)
3) Be deemed to require chemoradiotherapy.
4) Scheduled to receive 45Gy - 55Gy long course radiotherapy.
5) Have provided written informed consent to participate in the study.
6) Be aged 18 years or over.

The inclusion criteria above should be re-checked at the Randomisation visit.

6.2 EXCLUSION CRITERIA

Patients will **<u>not</u>** be eligible for the TRIGGER Trial, if they fulfil any of the following:

1) Have metastatic disease (including resectable liver metastases).
2) Are contraindicated for MRI e.g. non-MR compatible hip prosthesis, cardiac pacemaker.
3) Are scheduled to receive less than 45Gy or more than 55Gy long course radiotherapy.
4) Are contraindicated for chemoradiotherapy (CRT)
5) Hypersensitivity or contraindication to the drug(s) associated with the planned choice of systemic chemotherapy (CAPOX, FOLFOX or single agent 5-FU or capecitabine) as stated in the SmPC for each of the drugs.
6) Are receiving or planned to receive treatment outside of that stipulated by the protocol, such as an alternative cytotoxic or investigational drug.
7) Are pregnant, breastfeeding or unable / unwilling to comply with pregnancy prevention guidelines*.
8) Any other malignant disease within the preceding 5 years with the exception of non-melanomatous skin cancer, carcinoma in situ and early stage disease with <5% recurrence risk.

* Females of childbearing potential and males must be willing to use a highly effective method of contraception. Contraceptive methods that can achieve a failure rate of less than 1% per year when used consistently and correctly are considered as highly effective birth control methods. Contraceptive measures should be compliant with those described in the Summary of Product Characteristic for each of the IMPs used in this trial.

The exclusion criteria above should be re-checked at the Randomisation visit.

7. REGISTRATION AND RANDOMISATION

7.1 INFORMED CONSENT

The study will be conducted in accordance with the clinical trials regulations, the principles of GCP and the Declaration of Helsinki. Informed consent will be taken by the Principal Investigator or an appropriately qualified, trained and delegated clinician. The process for giving information to patients has a two-step staged approach to reduce the possibility of information overload and providing patients with plenty of time for consideration of participation in the main TRIGGER trial.

7.1.1 PATIENT INFORMATION SHEET 1 – REGISTRATION

This PIS contains general information about the main TRIGGER trial with consent for transfer of an archival block of tumour tissue to a central laboratory for further analysis, Quality of Life questionnaire completion,

the optional the collection of a blood sample for the assessment of proliferative, apoptotic and genomic markers, and consent to collect clinical outcome data regardless of if the patient is subsequently randomised into TRIGGER. This PIS is kept as minimal and as simple as possible whilst still providing sufficient information for the patient to give informed consent for registration. Suitable patients will be approached for entry into the registration period of TRIGGER at a routine outpatient clinic during the 4 weeks prior to commencing CRT. Once a patient is identified as potentially eligible, the rationale for this part of the study will be explained, the patient will be provided with Patient Information Sheet 1 and they will be given the opportunity to ask questions. The patient may then be consented for registration. When consent has been obtained and eligibility confirmed (using the Eligibility Checklist on the **TRIAL REGISTRATION Form**), baseline data should also be collected and recorded on the **TRIAL REGISTRATION Form**.

7.1.2 PATIENT INFORMATION SHEET 2 – RANDOMISATION

This PIS contains full details of the TRIGGER Trial and will be given to suitable patients during the registration period of TRIGGER either at an outpatient clinic or chemoradiotherapy appointment. Written informed consent for the main trial period will be obtained at a routine visit during CRT or once CRT is complete but at least 1 week prior to the post-CRT MRI scan.

Patients wishing to receive information about the main TRIGGER trial before providing consent for registration may receive Patient Information Sheet 2 at their first appointment if they wish.

The original signed consent forms at both registration and randomisation should be stored in the site investigator file. Four copies of the consent form should be made: one copy for the trial patient, one stored in the hospital notes, one copy sent to the local pathology lab for archived tissue release and one sent to the TRIGGER Trial Office for central monitoring processes.

If new information becomes available which may be relevant to the patient's consent, forms will be revised and informed consent sought again. Following consent and randomisation a letter must be sent to the patient's General Practitioner (Template provided at site initiation).

7.1.3 SCREENING, REGISTRATION AND RANDOMISATION LOGS

The PI or delegated staff at each site must record all eligible patients for trial registration who are discussed at the MDT, identified as potentially eligible on the baseline MRI report and/or identified by review of case notes. When patients are discussed but not approached or the patient chooses not to participate in the study please ensure these patients are still entered in the site screening log and the reason for non-inclusion documented. A registration log and randomisation log including reasons for non-inclusion should also be maintained for TRIGGER for patients registered and randomised. The reason and rate of non-recruitment at each step are important end points for the feasibility study.

7.2 PATIENT ID NUMBER

Each patient registered will be assigned a unique patient trial number at the time of registration with the TRIGGER Trial Office once eligibility has been confirmed. The trial number is a three-digit number, starting with 001 and increasing in ascending order, and is provided by the TRIGGER Trial Office at the Royal Marsden. This trial number will be a unique identifier and the primary way the patient will be identified during both the registration stage and following randomisation and should be used in all correspondence throughout the registration and subsequent trial periods. This trial number will be used to identify tumour blocks/slides and blood samples sent to the central laboratory. The patient's initials will also be used to cross-reference these samples.

7.3 REGISTRATION PROCEDURE

- Prior to registration confirm the patient's potential eligibility by reviewing history, diagnosis and treatment plan discussed at MDT referring to eligibility criteria in Section 6.
- Provide patient with PIS1 and seek patient's consent during the 4 weeks prior to CRT commencing.
- Once consent obtained, complete the Eligibility Checklist in the **TRIAL REGISTRATION Form** which must be signed by the registering investigator.
- Complete the remaining sections of the **TRIAL REGISTRATION Form** and **CONCURRENT MEDICATION Form**.
- Complete the **BASELINE MRI (PRE-CRT) Form**, which must be signed by the Radiologist.
- Ask patients to complete the **QUALITY OF LIFE Form** (EORTC QLQ-C30, LARS, EQ-5D).
- If patient consented to optional blood sample collection, obtain the required blood samples as described in section 10.4 and the Laboratory Manual.
- Submit the following documents by fax to the TRIGGER trial office at the Royal Marsden:
    1. Confirmation of patient consent for registration (Consent Form 1)
    2. TRIAL REGISTRATION Form
    3. CONCURRENT MEDICATION Form
    4. BASELINE MRI (PRE-CRT) Form
    5. QUALITY OF LIFE Form
    6. Histopathology report confirming diagnosis*
    7. MRI Report*
    8. A copy of the MDT Notes*

*** These documents should be anonymised prior to sending to the Trial Office by removing patient identifiers (including name, date of birth, NHS or hospital number) and adding trial name, site ID number and patient initials only.**

Send by fax to: Michelle Frost, Trial Manager, F: 0208 915 6721

Department of Radiology, Royal Marsden Hospital, Downs Road, Sutton, SM2 5PT

- Once the trial staff from the TRIGGER Trial Office have confirmed eligibility the site will be informed of the patient's allocated unique trial number (see Section 7.2 above). **This trial number should then be added to all trial-related patient documentation associated with registration including documents 1-8 listed above.**
- Contact local pathology department to arrange for the release of the tumour block for transfer to the central laboratory and provide them with a copy of the patient registration consent form.

7.3.1 HISTOPATHOLOGY INFORMATION AND CLINICAL INVESTIGATIONS REQUIRED AT REGISTRATION

- Histopathology - biopsy confirmed adenocarcinoma
  - date of biopsy, name of pathologist, tumour grade:
    - Well-differentiated adenocarcinoma
    - Moderately differentiated adenocarcinoma
    - Poorly differentiated adenocarcinoma
    - Undifferentiated
- Baseline MRI staging
- Results of the most recent routine blood tests performed prior to CRT as per local practice to determine there are no contraindications to planned choice of systemic chemotherapy.
- List of Concomitant medications including medication, dose, frequency and indication
- For female patient the result of most recent pregnancy test result (urine/blood) performed prior to commencing CRT as per local practice.

7.4 RANDOMISATION PROCEDURE

- Provide patient with PIS2 (Randomisation) at a routine outpatient appointment during CRT, ensuring patients are given a minimum of 24 hours between receiving this PIS and obtaining informed consent. It is anticipated that patients will receive PIS2 during the first three weeks of CRT allowing ample opportunity to ask questions and discuss the different treatment plans associated with randomization arm and the two interventional arm sub-groups.

- Once consent obtained complete the **RANDOMISATION Form** which must be signed by the PI (or delegate).
- Send the following documents to the TRIGGER Trial Office at the Royal Marsden, as soon as possible after consent has been obtained:
    - Confirmation of patient consent to randomisation (Consent Form 2)
    - **RANDOMISATION Form**

    Send by fax to: Michelle Frost, Trial Manager, F: 0208 915 6721

    Department of Radiology, Royal Marsden Hospital, Downs Road, Sutton, SM2 5PT
- Randomisation will occur once the trial staff have confirmed patient eligibility. Randomisation will be performed by the Clinical Trials & Statistics Unit at the Institute of Cancer Research using the stratified randomisation method described in section 3.2
- **The result of randomisation will be faxed to the site. This should be filed in the patient's source notes.**
- The following CRFs should also be completed and sent to the TRIGGER Trial Office:
    - **CONCURRENT MEDICATIONS Form**
    - **CHEMORADIOTHERAPY Form** (this should be completed once the patient has finished CRT)

7.4.1 ACTING ON THE RANDOMISATION

The post-treatment MRI forms are different for the control and intervention arms of the trial – named CONTROL ARM POST-TREATMENT MRI Form and INTERVENTION ARM POST-TREATMENT MRI Form for the control and intervention arm respectively. Therefore it is crucial that the research team at each site inform the radiologist which arm the patient is in. We recommend that this is done by adding the allocated arm of randomisation and which POST-TREATMENT MRI Form to use to the radiology request card. As this is an open label study, the randomisation outcome should also be recorded in the patient's medical records.

The **CONTROL ARM POST-TREATMENT MRI Form** for the control arm does not include the mrTRG classification. Without this information, treatment should be planned according to current guidelines i.e. only the information from the baseline MRI should be used. If the patient is randomised to the intervention arm, mrTRG directed management should take place.

7.4.2 REGISTERED PATIENTS WHO DO NOT CONSENT TO RANDOMISATION

Patients may register into TRIGGER but then decide not to consent to randomisation. Documenting the reasons for non-consent at this stage is important for assessing trial feasibility and therefore the reason for non-consent to randomization should be recorded on the Registration log.

7.5 WITHDRAWAL CRITERIA

By consenting to the trial, patients should understand that they are consenting to follow-up, data collection, additional MRI scans, additional endoscopy (in some patients) and the (optional) collection of biological samples for future research and (mandatory) central review of archived tissue samples.

Patients have the right to withdraw fully or partially from the study at any time and for any reason without prejudice to his or her future medical care. In cases of full withdrawal the **STUDY WITHDRAWAL Form** should be completed, including the primary reason for withdrawal, and details should be recorded in the patient's hospital records.

Withdrawal of consent to data collection

Withdrawal of **full** consent for the trial means that the patient does not wish to receive any further trial treatment and does not wish to or is unable to continue with further study participation. The Investigator should discuss with the patient the most appropriate way to withdraw to maintain the patient's care. In this case the patient's data already collected up to the point of withdrawal will still be included in the analysis of data unless the patient has explicitly requested that none of the data collected should be used for analysis. Once the **STUDY WITHDRAWAL Form** is completed no further CRFs should be completed and no further data sent to the Royal Marsden except for serious adverse event data, which will continue to be collected for 30 days after the last dose of systemic chemotherapy (IMP) is received, even if the patient has withdrawn from the study.

Withdrawal of partial consent

Withdrawal of **partial** consent means that the patient does not wish any further trial treatment any longer but is still willing to provide further data by continuing on study i.e. participate in subsequent study visits or procedures.

Replacement of patients following withdrawal

Patients who are withdrawn from the trial after randomization due to non-compliance with the protocol (section 7.5.1 below) or patients who withdraw full consent will be replaced in this feasibility trial.

7.5.1 NON COMPLIANCE WITH THE PROTOCOL

Patients will be withdrawn from the study if the post-CRT MRI is performed more than 10 weeks following the completion of CRT or a failure to comply with the deferral of surgery follow-up protocol. A **STUDY WITHDRAWAL Form** should be completed.

7.5.2 DISCONTINUATION OF TRIAL TREATMENT

A patient may discontinue trial treatment whenever continued treatment is no longer in the patient's best interests, but the reasons for doing so must be recorded on the **DISCONTINUATION OF TRIAL TREATMENT**

Form. The patient will continue to be followed-up unless they withdraw full consent to further data collection. If the patient gives a reason for their decision, this should be recorded. Reasons for discontinuing trial treatment may include:

1) The patient chooses to proceed to surgery despite clinical or radiological evidence of a complete response.
2) Disease progression whilst on therapy
3) Unacceptable toxicity
4) Treatment delayed by more than 3 weeks due to toxicity
5) Intercurrent illness which prevents further treatment
6) Any alterations in the patient's condition which justifies the discontinuation of treatment in the Site investigator's opinion
7) If a female patient becomes pregnant or fails to use adequate birth control (for patients of childbearing potential).
8) Any other reason that the patient specifies

In the above cases patients can remain within the trial for the purposes of follow-up and data analysis, unless full consent has withdrawn, according to the treatment option to which they have been allocated.

7.5.3 LOSSES TO FOLLOW-UP

If a patient moves from the area, every effort should be made for the patient to be followed up at another participating trial site and for this new centre to take over the responsibility for the patient, or for follow-up via GP. Details of participating trial sites can be obtained from the TRIGGER Trial Office at the Royal Marsden who must be informed of the transfer of care and follow up arrangements. If a patient is lost to follow-up at a site every effort should be made to contact the patient's GP to obtain information on the patient's status and a **STUDY WITHDRAWAL Form** should be completed.

8. SUMMARISED PATIENT PATHWAYS

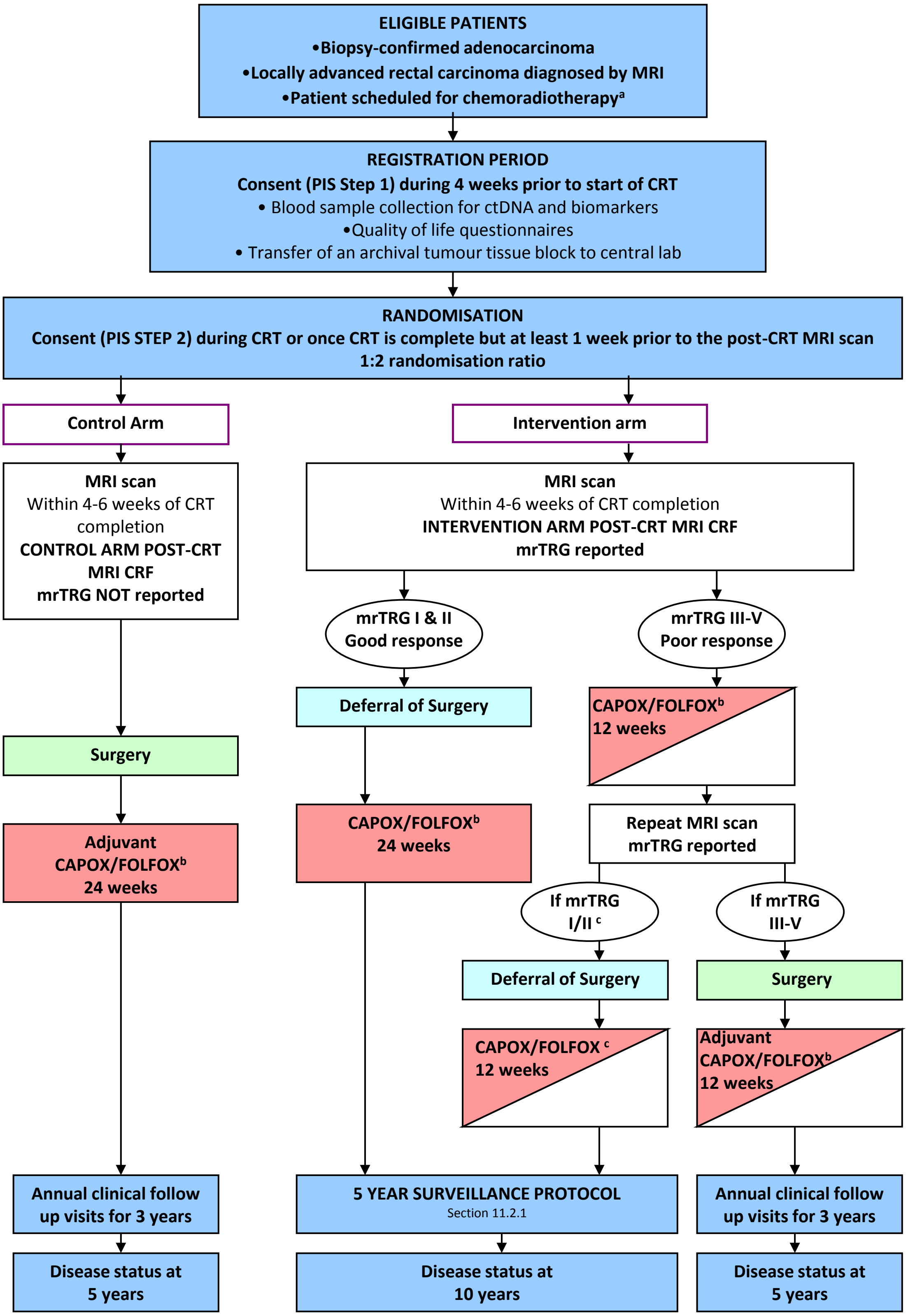

[a] Scheduled to receive 45 Gy-55Gy long course radiotherapy (inclusion criterion 4). [b] Treatment decision should be made prior to registration (planned choice is a randomisation stratification variable). Medical oncologist may choose to use CAPOX or FOLFOX, or single agent capecitabine or 5-FU if concomitant use of oxaliplatin is contraindicated. [c] If patient defers surgery then the remaining 12 weeks of chemotherapy should be given as soon as possible following the repeat MRI scan and MDT meeting .

The trial design gives rise to three possible pathways: Control Arm; Intervention Arm Good Response to CRT and Intervention Arm Poor Response to CRT. Investigators should ensure the patient pathways described in this protocol are followed. However, if an individual patient deviates from the planned pathway through their choice, e.g. patient in the 'good response' subgroup of the Intervention arm opts for surgery, then this should be recorded on the **PROTOCOL DEVIATION Form**.

All patients should follow the registration and randomisation procedure described in Section 7 of the protocol. Following Randomisation the PI at each site will be informed of which arm the patient has been randomised to and the appropriate patient pathway described below should be followed:

8.1 THE CONTROL ARM – BEST CURRENT PRACTICE

1. **Post-CRT restaging** – The post-CRT MRI must be **performed within 4-6 weeks (no later than 10 weeks)** from completion of CRT.
2. The radiologist will be notified that the patient has been randomised into the control arm. Radiologist completes the **CONTROL ARM POST-TREATMENT MRI Form**.
3. **Surgery** is planned according to the baseline MRI and should be performed 6-12 weeks after CRT completion. A prepared **SURGERY Form** should be enclosed in the patient notes so that the surgeon can complete the form immediately after surgery.
4. A routine surgical follow up appointment will occur 4-6 weeks after surgery, therefore the **SURGICAL MORBIDITY** and **PATHOLOGY REPORTING Forms** should be completed at 6 weeks. These forms may be completed retrospectively up to 12 weeks after surgery but only complications that occur within the first 6 weeks should be recorded on the **SURGICAL MORBIDITY Form**.
5. **Adjuvant Chemotherapy** - Please complete the **CHEMOTHERAPY and TOXICITY Forms** at the end of each cycle i.e. at the end of each 3-weekly cycle of CAPOX (or single agent capecitabine) or at the end of each 2-weekly cycle of FOLFOX (or single agent 5-FU).
6. **Oncological outcomes** – 12 months from the completion of CRT please complete the **ANNUAL FOLLOW-UP Form**, and further **SURGICAL MORBIDITY** and **TOXICITY Forms** to capture late surgical complications and residual toxicity respectively. To avoid the inevitable distress caused by the relative of a dead patient receiving questionnaires, please ensure this is completed in parallel with the follow-up for Patient Reported Outcome Measures (PROMs).
7. **PROMs** – 12 months from the completion of CRT, the recruiting trial site will need to invite the patient to complete a postal questionnaire (**QUALITY OF LIFE forms**) that includes 40 questions about symptoms and quality of life. The patient will have completed the questionnaires at entry into the study and will be expecting to receive the forms. A freepost envelope addressed to the recruiting trial site will also be provided. If the patient does not initially respond please contact them by phone – to

confirm the questionnaires arrived and to re-send if necessary. If there is no response send a second questionnaire 2 weeks later. If there is no response by 12 weeks (15 months from the end of CRT), we will accept that the patient preferred not to take part. If the patient has a disability that prohibits them from completing the forms by hand we recommend reading the questions out over the phone.

8. **Oncological outcomes** – 24 months and 36 months from the completion of CRT please complete the **ANNUAL FOLLOW-UP Form**.
9. **PROMs** - 36 months (not at 24 months) from the completion of CRT the patients will be asked to completed the same **QUALITY OF LIFE Form**.

8.2 INTERVENTION ARM – A 'GOOD RESPONSE' TO CHEMORADIOTHERAPY (MRTRG 1 & 2)

1. **Post-CRT restaging** – The post-CRT MRI must be **performed within 4-6 (no later than 10) weeks** of completing CRT. Ensure the Radiologist receives the appropriate reporting form – **INTERVENTION ARM POST-TREATMENT MRI** Form which includes the mrTRG datafields.
2. Radiologist completes the INTERVENTION ARM POST-TREATMENT MRI Form – **mrTRG 1 or 2 reported mrTRG directed management results in the patient being offered deferral of surgery.**
3. The option of deferral of surgery is discussed with the patient, and intensive active monitoring is undertaken. **Please refer to the 5 year surveillance schedule in Section 11.2 Deferral of Surgery Follow-up**. The **INTERVENTION ARM POST-TREATMENT MRI Form** should be completed each time an MRI scan is performed during the surveillance period.
4. **Chemotherap**y (Appendix 2) – The patient will receive 24 weeks of chemotherapy (8 cycles of CAPOX or 12 cycles of FOLFOX) and this should start within 12 weeks of CRT completion. Please complete the **CHEMOTHERAPY AND TOXICITY Forms** at the end of each cycle i.e. at the end of each 3-weekly cycle of CAPOX (or single agent capecitabine) or at the end of each 2-weekly cycle of FOLFOX (or single agent 5-FU).
5. **Mid-chemotherapy MRI scan** – an MRI scan should be performed approximately mid-way through chemotherapy treatment once the patient has received 12 weeks of treatment. The **INTERVENTION ARM POST-TREATMENT MRI Form** should be completed**.**
6. **Oncological outcomes**– 12 months from the completion of CRT please complete the **ANNUAL FOLLOW-UP Form**, and a further **TOXICITY Form** to assess residual toxicity. To avoid the inevitable distress caused by the relative of a dead patient receiving questionnaires, please ensure this is completed in parallel with the follow-up for Patient Reported Outcome Measures (PROMs).
7. **PROMs** -12 months from the end of CRT, the recruiting trial site will need to invite the patient to complete a postal questionnaire (**QUALITY OF LIFE Form**) that includes 40 questions about symptoms and quality of life. The patient will have completed the questionnaire at entry into the study and will

be expecting to receive the forms. A freepost envelope addressed to the recruiting trial site will also be provided. If the patient does not initially respond please contact them by phone – to confirm the questionnaires arrived and to re-send if necessary. If there is no response send a second questionnaire at 2 weeks later. If there is no response by 12 weeks (15 months from the completion of CRT), we will accept that the patient preferred not to take part. If the patient has a disability that prohibits them from completing the forms by hand we recommend reading the questions out over the phone.

8. **Oncological outcomes** – 24, 36, 48 and 60 months following the completion of CRT please complete the **ANNUAL FOLLOW-UP Form**.
9. **PROMs** - 36 months (not at 24 months) and 60 months from the completion of CRT the patients will be asked to complete the same postal questionnaire (**QUALITY OF LIFE Form**).

8.2.1 PROCEDURE IF A PATIENT DECLINES DEFERRAL OF SURGERY

Complete a **PROTOCOL DEVIATION Form**. Follow-up and completion of CRFs should be carried out according to the control arm (Section 10.1, Assessment Schedule Control Arm and Section 11.1, Post-Surgery Follow Up). The patient is still eligible for analysis on an intention to treat basis.

8.2.2 PROCEDURE IF A LOCAL REGROWTH IS DETECTED

<u>Patients with evidence of clinical or radiological local regrowth or pelvic relapse must be treated at least as urgently as a primary rectal cancer by the surgical team</u>. Refer to suspected regrowth pathway in Section 8.2.3 below. Complete a **SURVEILLANCE CRF**. If patient proceeds to surgery Follow-up and CRF completion should be carried out according to the Control arm (Section 10.1, Assessment Schedule Control Arm and Section 11.1, Post-Surgery Follow Up). The **SURGERY, SURGICAL MORBIDITY** and **PATHOLOGY REPORTING Forms** should be completed when the patient undergoes surgery. If the colorectal MDT decision is to offer adjuvant therapy then the **CHEMOTHERAPY** and **TOXICITY Forms** should also be completed during treatment. The timing of the oncological outcomes, assessment of late surgical complications and PROMs should be completed as per protocol.

If patient refuses surgery or is not fit for surgery then a **DISCONTINUATION OF TRIAL TREATMENT Form** should be completed and the patient followed-up unless they withdraw consent to further data collection.

The patient is still eligible for analysis on an intention to treat basis.

8.2.3 SUSPECTED REGROWTH PATHWAY FOR PATIENTS WHO DEFER SURGERY

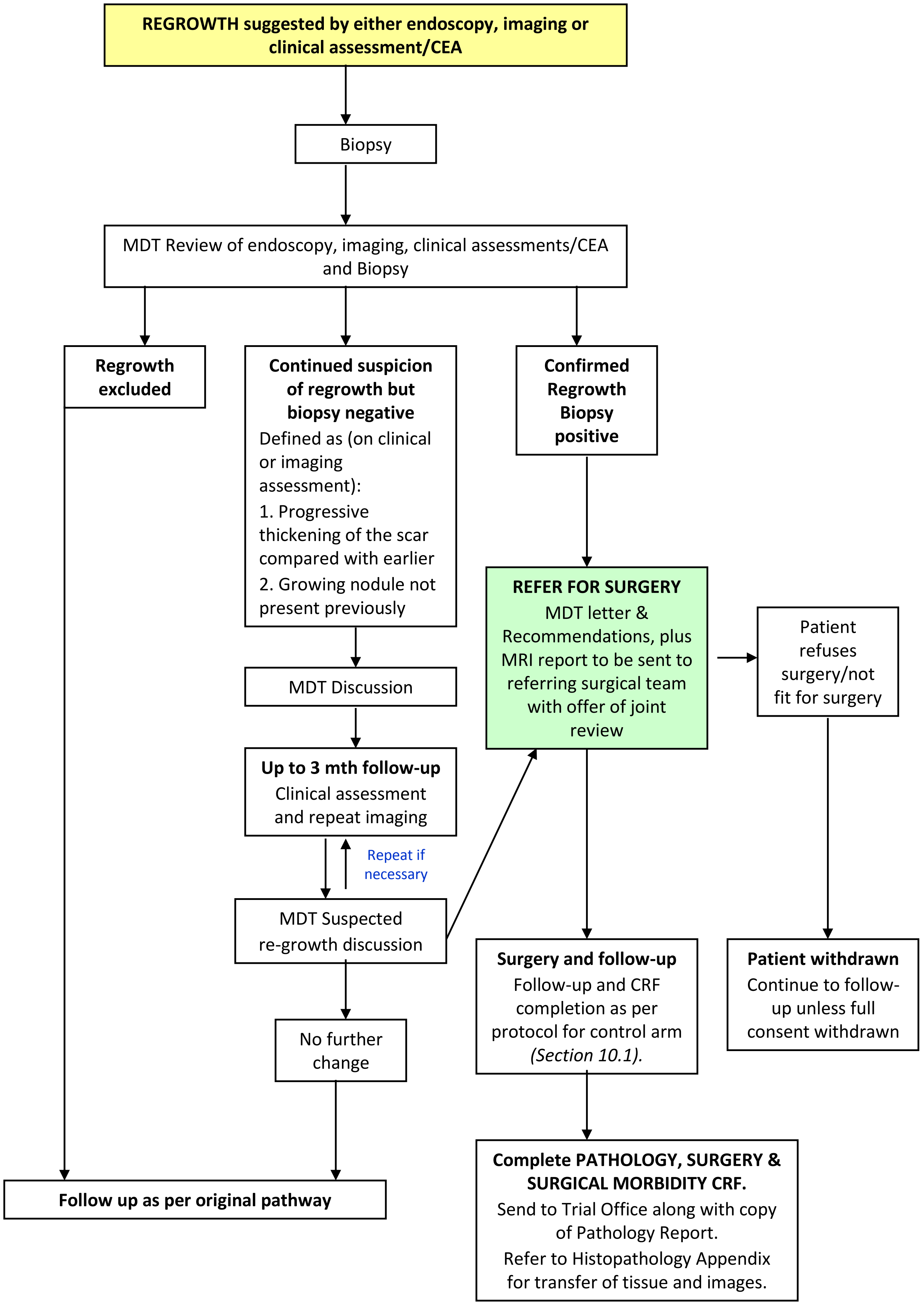

8.3 EXPERIMENTAL ARM – A 'POOR RESPONSE' TO CHEMORADIOTHERAPY

1. **Post-CRT restaging** – The post-CRT MRI must be performed within 4-6 (no later than 10) weeks of completing CRT.
2. Radiologist completes the **INTERVENTION ARM POST-TREATMENT MRI Form** – **<u>mrTRG 3 –5 reported.</u>**
3. **<u>mrTRG directed management results in the patient being offered additional chemotherapy before a decision is made regarding surgery.</u>**
4. The option of **additional pre-operative chemotherapy** is discussed with the patient. **Chemotherapy** (Appendix 2) – The patient will receive 12 weeks (4 cycles of CAPOX or 6 cycles of FOLFOX); this should start within 12 weeks of CRT completion. Please complete the **CHEMOTHERAPY** and **TOXICITY Forms** at the end of each cycle i.e. at the end of each 3-weekly cycle of CAPOX (or single agent capecitabine) or at the end of each 2-weekly cycle of FOLFOX (or single agent 5-FU).
5. Following the completion of 12 weeks of chemotherapy a repeat MRI should be performed 4-6 weeks later and mrTRG reported. Ensure the Radiologist receives the appropriate reporting form – **INTERVENTION ARM POST-TREATMENT MRI Form** which includes the mrTRG data fields. The Radiologist completes the **INTERVENTION ARM POST-TREATMENT MRI Form**.

<u>mrTRG directed management results in the patient being offered deferral of surgery (if mrTRG improved to I or II) or proceeding to surgery (mrTRG remains III-V). The options of surgery or deferral of surgery as appropriate should be discussed with the patient.</u>

- **If mrTRG now I or II and it is agreed that patient should defer surgery then patients should receive the remaining 12 weeks of chemotherapy as soon as possible following the repeat MRI and MDT meeting and be followed up in the same way as those in the 'good-response' sub-group described above and undergo the 5 year surveillance schedule detailed in Section 11.2.1.**

- **If mrTRG remains III-V then patient proceeds to surgery and undergoes the following:**

6. **Surgery** is planned according to the baseline and post-treatment MRI and should be performed **6-12 weeks after pre-operative chemotherapy** is completed. A prepared **SURGERY Form** should be enclosed in the patient notes so that the surgeon can complete the form immediately after surgery.
7. A routine surgical follow up appointment will occur 4-6 weeks after surgery, therefore the **SURGICAL MORBIDITY** and **PATHOLOGY REPORTING Forms** will be completed at 6 weeks. The **SURGICAL MORBIDITY Form** may be completed retrospectively up to 12 weeks after surgery but only complications that occur within the first 6 weeks should be recorded.

8. **Adjuvant Chemotherapy** - The patient will receive 12 weeks (4 cycles of CAPOX or 6 cycles of FOLFOX). Please complete the **CHEMOTHERAPY** and **TOXICITY Forms** at the end of each cycle i.e. at the end of each 3-weekly cycle of CAPOX (or single agent capecitabine) or at the end of each 2-weekly cycle of FOLFOX (or single agent 5-FU).
9. Oncological outcomes – 12 months from the end of CRT please complete the ANNUAL FOLLOW-UP Form, and a further **SURGICAL MORBIDITY** and **TOXICITY Forms** to capture late surgical complications and residual toxicity respectively. To avoid the inevitable distress caused by the relative of a dead patient receiving questionnaires, please ensure this is completed in parallel with the follow-up for Patient Reported Outcome Measures (PROMs).
10. **PROMs** -12 months from the end of CRT, the recruiting trial site will need to invite the patient to complete a postal questionnaire (**QUALITY OF LIFE Form**) that includes 40 questions about symptoms and quality of life. The patient will have completed the questionnaire at entry into the study and will be expecting to receive the forms. A freepost envelope addressed to the recruiting trial site will also be provided. If the patient does not initially respond please contact them by phone – to confirm the questionnaires arrived and to re-send if necessary. If there is no response send a second questionnaire at 2 weeks later. If there is no response by 12 weeks (15 months from the end of CRT), we will accept that the patient preferred not to take part. If the patient has a disability that prohibits them from completing the forms by hand we recommend reading the questions out over the phone.
11. **Oncological outcomes** – 24 months and 36 months following the end of CRT please complete the **ANNUAL FOLLOW-UP Form**.
12. **PROMs** - 36 months (not at 24 months) following the end of CRT the patients will be asked to complete the same **QUALITY OF LIFE form**.

9. TRIAL TREATMENT

Full details relating to chemotherapy treatment duration, dose schedules, administration and modifications, interactions with other therapies and concomitant medications can be found in Appendix 2. Expected adverse events associated with CAPOX and FOLFOX regimes are included in Appendix 1. The expected protocols for long course radiotherapy can be found in Appendix 3.

9.1 TREATMENT DURING CHEMORADIOTHERAPY

The planned choice of chemotherapy treatment during CRT is at the discretion of the local oncology team. Although no treatments are under evaluation during the registration period, details relating to the CRT should be recorded in the **CHEMORADIOTHERAPY Form** once the patient completes CRT.

9.2 TRIAL INVESTIGATIONAL MEDICINAL PRODUCTS

The choice of systemic chemotherapy (CAPOX, FOLFOX or single agent capecitabine or 5-FU) is a stratification variable for randomisation and therefore the chemotherapy regimen to be used should be made upfront prior to patient registration according to patient choice or local practice.

For the purpose of this trial all drugs associated with the CAPOX and FOLFOX chemotherapy regimes, listed below, are considered IMPS:

- Oxaliplatin
- Capecitabine
- Fluorouracil (5-FU)
- Folinic acid (given in combination with Oxaliplatin and 5-FU in the FOLFOX regime)

Trial sites can use any brand of the IMPs listed above according to local practice.

9.3 DISPENSING AND DRUG SUPPLIES

A TRIGGER Trial pharmacy pack including details relating to the IMPs used in this trial will be sent to participating sites prior to site initiation. All trial drugs used in this trial are commercially available and each site will use their normally available generic hospital stock and their handling and management should be subject to standard local pharmacy procedures. Each trial site pharmacy will be responsible for drug accountability and destruction at their site as detailed in the TRIGGER pharmacy pack. All sites will ensure that all stock used will be of suitable quality as per EU GMP and only use stock that has a UK or EU marketing authorisation.

9.4 SUMMARY OF PRODUCT CHARACTERISTICS

The reference document relating to each of the IMPs listed in section 9.2 is the SmPC. Section 4.8 (Undesirable effects) of the SmPC represents the reference safety information for each of the IMPs, which should be used by investigators as a reference point when assessing causality and expectedness.

**Investigational medicinal product**	**Brand**
Capecitabine 150mg film coated tablet	Accord
Capecitabine 500mg Film coated tablet	Accord
Fluorouracil 25mg/ml Injection	Hospira UK
Fluorouracil 50mg/ml Injection	Hospira UK
Oxaliplatin 5mg/ml Concentrate for Solution for Infusion	Hospira UK
Calcium folinate 10mg/ml Injection	Hospira UK

10. ASSESSMENT SCHEDULES

Please refer to the assessment schedules below for details of assessments to be performed, timelines and the CRFs to complete (further details relating to CRF completion can be found at the front of the CRF pack).

- Radiological assessment will be performed as per Appendix 6 with synoptic reporting according to the allocated **POST-TREATMENT MRI FORM**.
- Chemotherapy toxicity will be assessed at the end of each cycle during treatment and residual toxicity assessed at the 12-month follow-up visit.
- When surgery is performed, surgical morbidity will be recorded intra-operatively and both early (6 weeks) and late (up to 12 months) surgical complications will be recorded post-operatively.
- **The QUALITY OF LIFE Form** will be completed by all patients at registration. For patients that undergo surgery the QoL forms should be completed again at 1 and 3 years following the completion of CRT. For patients who defer surgery the QoL forms should be completed at 1, 3 and 5 years from the time of CRT completion.
- Adverse events should be recorded from the date of the post-CRT MRI scan until 30 days after the patient's last dose of chemotherapy received during the intervention phase of the trial. Residual toxicity will be assessed at 12 months. Concurrent medications should be recorded at registration and updated from randomization until 30 days after the patient's last dose of chemotherapy. .
- **ANNUAL FOLLOW-UP Forms** should be completed at 12 months from the end of CRT and annually thereafter up to 3 years in those that undergo surgery and up to 5 years in those who defer surgery.

10.1 ASSESSMENT SCHEDULE SUMMARY: CONTROL ARM

CONTROL ARM		REGISTRATION PERIOD	INTERVENTION PHASE						ANNUAL FOLLOW-UP			DISEASE STATUS[g]
VISIT TYPE	Prior to patient entry	Registration	Randomisation (BASELINE)	Post CRT	MDT	Surgery	Surgical follow up	Adjuvant Chemotherapy for 24 weeks	12	24	36	60
TIMELINES		≤ 4 weeks prior to CRT	During CRT[h]	4-6 weeks post CRT		6-12 weeks post CRT	6 weeks post surgery	Toxicity assessed at end of each cycle during chemotherapy	Months from end of CRT			
Informed consent[a]		X	X									
Check eligibility criteria		X	X									
Diagnosis, History & Clinical Assessment		X										
Randomisation			X									
Quality of life		X							X		X	
Chemoradiotherapy			X									
Blood sample[b]		X		X			X	X	X	X	X	
Baseline MRI	X											
Restaging MRI[c]				X								
Surgery						X						
Surgical Morbidity[d]							X		X			
Pathology[e]							X					
Chemotherapy								X end of each cycle				
Toxicity assessment								X end of each cycle	X			
Annual Follow-up						X			X	X	X	X
Adverse events[f]				X		X	X	X end of each cycle				
Concurrent medications		X	X	X		X	X	X end of each cycle				

The X also denotes that CRFs need competing - tick or initial the boxes as the CRFs are completed.

[a] Eligible subjects will be asked to provide written informed consent at registration and at randomization.

[b] if patient has consented to additional blood sample collection for research.

[c] The post-CRT MRI should to be performed within 4-6 weeks (maximum of 10 weeks) from completion of CRT.
[d] Both early (4-6 weeks) and late surgical complications (at 12 months) will be recorded.
[e] Resected specimen will be prepared and evaluated using a standardized method described in Appendix 5.
[f] All adverse events will be recorded from the date the Post-CRT MRI scan is performed until 30 days after the last dose of chemotherapy is administered during the Intervention Phase of the trial.
[g] Disease status at 5 years (does not require clinic visit): alive without metastatic or recurrent disease, alive with metastatic and/or recurrent disease (date diagnosed), dead (date of death)
[h] Patient consent to randomisation can take place at any time during CRT or once CRT is complete but at least 1 week prior to the post-CRT MRI scan

10.2 INTERVENTION ARM - 'GOOD RESPONSE' TO CHEMORADIOTHERAPY

INTERVENTION ARM GOOD RESPONSE		REGISTRATION PERIOD	INTERVENTION PHASE					FOLLOW-UP	DISEASE STATUS[g]
VISIT TYPE	Prior to patient entry	Registration	Randomisation (BASELINE)	Post CRT	MDT		Chemotherapy for 24 weeks [f]	**Follow surveillance schedule for patient who defer surgery (Section 11.2.1) for a period of <u>5 years</u> from end of chemotherapy. Complete SURVEILLANCE Form at each follow-up visit.**<br><br>**If surgery is undertaken** (patient declines deferral of surgery or regrowth is detected during follow-up) then the follow-up schedule for the control arm should be used. The Surgery and Surgical Morbidity CRFs should be completed as per control schedule. In the case of regrowth a SURVEILLANCE CRF should also be completed.	10 YEARS
TIMELINES		≤ 4 weeks prior to CRT	During CRT[h]	4-6 weeks post	Review of restaging MRI including mrTRG reporting. mrTRG directed management results in option of deferral of surgery (mrTRG I&II). The option of deferral of surgery is discussed with patient.	No surgery	≤12 weeks post CRT. Toxicity assessed at end of each cycle during chemotherapy		From end of CRT
Informed consent [a]		X	X						
Check eligibility criteria		X	X						
Diagnosis & Clinical Assessment		X							
Randomisation			X						
Quality of life		X							
Chemoradiotherapy			X						
Blood sample [b]		X		X			X		
Baseline MRI	X								
Restaging MRI [c]				X			X		
Pathology [d]									
Chemotherapy							X end of each cycle		
Toxicity Assessment							X end of each cycle		
Annual Follow-up									X
Adverse events [e]				X			X end of each cycle		
Concurrent medications		X	X	X			X end of each cycle		

The X also denotes that CRFs need competing - tick or initial the boxes as the CRFs are completed.

[a] Eligible subjects will be asked to provide written informed consent at registration and at randomization.

[b] if patient has consented to additional blood sample collection for research.

[c] The post-CRT MRI should to be performed within 4-6 weeks (maximum of 10 weeks) from completion of CRT. A further MRI should be performed mid-way through chemotherapy treatment at approximately 12 weeks.

[d] Resected specimen will be prepared and evaluated using a standardized method described in Appendix 5.

[e] All adverse events will be recorded from the date the Post-CRT MRI scan is performed until 30 days after the last dose of chemotherapy is administered during the Intervention Phase of the trial.
[f] Initial staging indicated these tumours were locally advanced, therefore all patients are offered systemic chemotherapy regimen equivalent to post-operative adjuvant chemotherapy. If regrowth occurs during chemotherapy patient should proceed to surgery and discuss the pathology at MDT to decide if remaining cycles should be given post-operatively.
[g] Disease status at 10 years (does not require clinic visit): alive without metastatic or recurrent disease, alive with metastatic and/or recurrent disease (date diagnosed), dead (date of death)
[h] Patient consent to randomisation can take place at any time during CRT or once CRT is complete but at least 1 week prior to the post-CRT MRI scan

10.3 INTERVENTION ARM -'POOR RESPONSE' TO CHEMORADIOTHERAPY

**INTERVENTION ARM POOR RESONDERS**		REGISTRATION PERIOD	INTERVENTION PHASE								ANNUAL FOLLOW-UP			DISEASE STATUS[i]
VISIT TYPE	Prior to patient entry	Registration	Randomisation (BASELINE)	Post CRT	MDT	Chemo-therapy for 12 weeks	MDT	Surgery	Surgical follow up	Adjuvant Chemotherapy for 12 weeks	12	24	36	60
TIMELINES		≤ 4 weeks prior to CRT	During CRT[j]	4-6 weeks post CRT		≤ 12 weeks post CRT. Toxicity assessed at end of each cycle	A further MRI scan should be performed 4-6 weeks from end of chemotherapy. If mrTRG now I or II the option of deferral of surgery should be discussed. If patients defers surgery patients should receive the remaining 12 weeks of chemotherapy and follow surveillance schedule in section 11.2.1 and assess disease status at 10 years (as for good response sub-group). If surgery performed follow-up according to this table.	6-12 weeks after pre-op chemo	6 weeks post surgery	Toxicity assessed at end of each cycle during chemotherapy	Months from end of CRT			
Informed consent [a]		X	X											
Check eligibility criteria		X	X											
Diagnosis & Clinical Assessment		X												
Randomisation			X											
Quality of life		X									X		X	
Chemoradiotherapy			X											
Blood sample [b]		X		X		X			X	X	X	X	X	
Baseline MRI	X													
Restaging MRI [c]				X		X[h]								
Surgery								X						
Surgical Morbidity [d]									X		X			
Pathology [e]									X					
Chemotherapy [f]						X end of each cycle				X end of each cycle				
Toxicity Assessment						X end of each cycle				X end of each cycle	X			
Annual follow-up											X	X	X	X

Adverse events [g]				X		X end of each cycle				X end of each cycle				
Concurrent medications		X	X	X		X end of each cycle		X	X	X end of each cycle	X			

The X also denotes that CRFs need competing - tick or initial the boxes as the CRFs are completed.

[a] Eligible subjects will be asked to provide written informed consent at registration and at randomization.

[b] if patient has consented to additional blood sample collection for research.

[c] The post-CRT MRI should to be performed within 4-6 weeks (maximum of 10 weeks) from completion of CRT.

[d] Both early (4-6 weeks) and late surgical complications (up to 12 months) will be recorded.

[e] Resected specimen will be prepared and evaluated using a standardized method described in Appendix 5.

[f] Chemotherapy toxicity is assessed every 6 weeks during chemotherapy treatment. 12 weeks (6 cycles of FOLFOX or 4 cycles of CAPOX) are given pre-operatively and 12 weeks (6 cycles of FOLFOX or 4 cycles of CAPOX) are given post-operativey.

[g] All adverse events will be recorded from the date the Post-CRT MRI scan is performed until 30 days after the last dose of chemotherapy is administered during the Intervention Phase of the trial.

[h] A further MRI scan should be performed within 4-6 weeks from completion of pre-operative chemotherapy and mrTRG reported.

[i] Disease status at 5 years (does not require clinic visit): alive without metastatic or recurrent disease, alive with metastatic and/or recurrent disease (date diagnosed), dead (date of death)

[j] Patient consent to randomisation can take place at any time during CRT or once CRT is complete but at least 1 week prior to the post-CRT MRI scan

10.4 BIOMARKER DEVELOPMENT

All patients will be asked to consent to the collection of additional blood samples at trial registration and randomisation for a translational sub-study (Appendix 7), which will be collected at the following time points:

- Registration
- 4-6 weeks post CRT
- For patients who undergo surgery:
  - 4-8 weeks post surgery
  - 3 months post surgery (for most patients this will be during adjuvant chemotherapy)
  - Annual follow-up visits at 12, 24 and 36 months.
- For patients who defer surgery
  - At follow-up visits (as per surveillance schedule) from end of CRT at 6, 9, 12, 18, 24, 30 and 36 months, and at 4 and 5 years.
- For all patients who relapse during trial:
  - Within 2 weeks of tumour relapse

Participants are also asked to consent to donate their diagnostic paraffin embedded tumour tissue (FFPE blocks) and H&E slides at registration and at the time of surgery to be used in the translational sub-study (Appendix 7) to evaluate the role of prognostic and predictive biomarkers. Details relating to the histopathology evaluation of the resected specimen can be found in Appendix 5.

10.4.1 BLOOD SAMPLES FOR BIOMARKER ANALYSIS

Site sample collection

The specimens should only be collected if the patient consents to the collection of a sample at registration and for follow-up samples at randomisation.

Label 2 x 10 ml cell free DNA^{TmSteck} tubes (Streck tubes) with the trial-specific label provided and add site number, patient ID number and initials, visit date and visit time-point. Blood samples should be collected at the time points outlined in section 10.4 above. Ideally this should occur at the same time as other blood tests in order to avoid the need for an additional venepuncture. Samples should be sent to the Royal Marsden within 48 hours of collection.

Please see the **Laboratory Manual** for full details on the collection, processing, storage and shipment of the blood samples. The manual also includes details of blood sample collection for patients who have co-enrolled into TRACC Trial.

Sample processing at RM

At the RM, once the samples have been received they will be catalogued, processed and stored.

Further information on the collection, processing and storage of the blood samples at the lead centre can be found in the Laboratory Manual.

10.4.2 H&E STAINED GLASS SLIDES

All of the H&E stained glass slides from both the original diagnostic biopsy and the surgical resection specimens should be sent to the Royal Marsden for scanning. Copies of the slides can be sent if the local site does not want to release the originals. Alternatively high resolution digital slides can be sent if slide scanning facilities are available locally.

10.4.3 PARAFFIN-EMBEDDED DIAGNOSTIC TUMOUR TISSUE

Appropriately labelled tissue blocks of the original diagnostic biopsy should be sent to the Royal Marsden **within 28 days** of patient registration for central archiving, or at the time of randomisation if not sent previously. Blocks should also be sent at the time of surgery as detailed in Appendix 5.

Please see the **Laboratory Manual** for guidance on collection, processing, shipment and storage of the tissue samples.

This tissue will be used to assay for the presence of biomarkers which will be correlated to outcome and response to treatment to evaluate their potential prognostic and predictive role. Other biomarkers which will be examined are to be determined at a later date and may include components of signalling pathways which have a role in cancer development or progression.

10.4.4 SAMPLE PROCESSING AT ROYAL MARSDEN

All blood and tissue samples will be stored at the Royal Marsden until analysis, which will take place at the end of the feasibility trial. Measurements of ctDNA will be carried out at the Institute of Cancer Research/Royal Marsden BRC. The H&E Slides will sent to the Dept of Pathology and Tumour Biology at St James' Hospital in Leeds and will be digitised and archived. The FFPE blocks will be sent to the ICR/Royal Marsden BRC for DNA and RNA extraction and then sent to the University of Southampton for the preparation and analysis of tissue microarrays (TMAs). TMAs will be sent to the ICR/Royal Marsden BRC, St James' Hospital Leeds and the Institute of Molecular Sciences at Radboud University, Nijegan for further analysis.

Following analysis, surplus blood and tumour tissue will be returned to the Royal Marsden for storage for future research, subject to funding and approval by members of the TMG.

10.4.5 SAMPLE LABELLING, STORAGE AND DESTRUCTION

In order to protect patient identity, blood, paraffin embedded tumour blocks and H&E slides should not be labelled with any information which may lead to the direct identification of the patient concerned,

including patient name, date of birth, or National Health Service (NHS) or hospital number. Instead samples should be labelled with the study name, site number, the patient's study number (assigned at registration), and patient initials. The date of collection should also be recorded on blood samples. Centres should also keep a record of blood and tissue samples collected on the Blood & Tissue Sample Log (template included in the Laboratory Manual) which includes the same information. In addition, the date of transfer of samples to the lead centre should be recorded.

Prior to transfer to the lead centre, centres should ensure that blood and tumour samples are appropriately stored as detailed in the Laboratory Manual. Blood and tissue samples received by the lead site will be stored indefinitely. However, the patient retains the right to have the sample material returned to their hospital pathology department or destroyed at any time by contacting the principal investigator at the site at which they were registered for the study. The site principal investigator will then be responsible for contacting the sponsor via the chief investigator to arrange for the return or destruction of the samples.

The sponsor will be the exclusive owner of any data, discoveries, or derivative materials from the sample materials and is responsible, via the chief investigator, for the destruction of the sample(s) at the request of the research patient through the site principal investigator or at the end of the storage period. The site principal investigator will provide the chief investigator with the required patient study numbers so that any unused blood and tissue samples can be located and destroyed. If a commercial product is developed from this research project, the sponsor will own the commercial product. The patient will have no commercial rights to such product and will have no commercial rights to the data, information, discoveries, or derivative materials gained or produced from the sample.

11. CLINICAL FOLLOW-UP

Once randomised, patients remain evaluable for the intent-to-treat analysis regardless of their subsequent course and treatment. Follow-up data on all patients, including details of other treatments given, is therefore important.

All patients that undergo surgery will be followed up for 5 years. Patients that defer surgery will be followed up for 10 years. In addition, where available, national mortality records and hospitals episodes statistics will be used to ensure long-term follow-up. If a patient fails to attend a clinic or cannot be followed up, the site should contact the patient's GP to assess their condition.

Summary of Clinical Investigations for all patients

Clinical investigations performed prior to CRT, during CRT, before and after surgery and during chemotherapy should be performed as per standard clinical practice and as clinically indicated, including

but not limited to clinical examinations, performance status, vital signs , ECG, routine blood tests, pregnancy tests for women of childbearing potential, measurements of CEA (see section below), imaging and colonoscopy.

A baseline MRI and post-CRT MRI are mandatory for all subjects as included in the Assessments schedules in Section 10. Patients in the 'poor response' subgroup of the intervention arm should also receive a second post treatment MRI scan following pre-operative systemic chemotherapy and mrTRG reported for review at the MDT reassessment meeting (Section 10.3).

Patients managed with deferral of surgery require additional monitoring as detailed in section 11.2.1 below.

Investigation of a rising CEA during follow-up

If a patient has a rising CEA during follow up, the following algorithm of investigation is recommended:

1. A CT chest/abdomen/pelvis should be the first-line investigation. The scan should be reviewed by an experienced GI radiologist (e.g. the study radiologist).

2. If no pelvic relapse is seen and no distal metastasis, particularly in the liver, then patients should proceed to an MRI pelvis and an MRI of the liver with liver specific contrast.

3. If no cause for the elevated CEA is found after MRIs, then a FDG-PET scan is recommended.

All imaging performed to investigate a rising CEA should be completed as per standard radiology guidelines,

Investigations for a suspected pelvic/distant relapse

The pelvic/distant relapse should be discussed at an MDT and investigations, including imaging, and surgery if indicated, should be performed according to local practice. Patients with evidence of curative disease will continue to be followed up and information about further treatment captured on the **ANNUAL FOLLOW-UP Form**. If the disease is only considered amenable to palliative care then the patient should discontinue trial treatment and be followed up annually (by completion of the **ANNUAL FOLLOW-UP Form**) unless full consent for data collection is withdrawn. **Patients with evidence of suspected pelvic relapse must be treated at least as urgently as a primary rectal cancer by the surgical team.**

11.1 POST-SURGERY FOLLOW-UP

Follow-up after surgery will include regular clinical follow-up as per usual practice. A CT scan should be carried out after surgery and following the completion of chemotherapy as per usual practice. It is recommended that CEA should be assessed every 6 months and thorax/abdominal/pelvis CT scans performed annually for the first 3 years, or as clinically indicated. The primary outcome of the main Phase III TRIGGER Trial compares the disease-free survival at 3 years from the start of chemoradiotherapy. A full investigation for recurrent disease (including CEA and a pelvis/thorax/abdomen CT scan, colonoscopy for metachronous disease) should be undertaken at this time point. The use of investigations after 3 years is left to clinical discretion but disease status for assessment of overall survival and disease free survival will

be collected at 5 years from the surgical clinics/GP patient records. The information routinely recorded in normal clinical notes should be sufficient for completion of the **ANNUAL FOLLOW-UP Form**.

11.2 DEFERRAL OF SURGERY FOLLOW-UP

Patients are reviewed in clinic at regular intervals for 5 years. Clinical assessment includes a digital rectal examination (DRE) with a blood test for CEA at each clinic visit. Patients will have an MRI scan approximately mid-way through chemotherapy treatment once they have received their first 12 weeks of therapy.

After completion of chemotherapy, patients will be reviewed every 3 months until 2 years from the completion of CRT and then every 6 months for a further 3 years. The full details of the timings of out-patient clinics, MRI, CT and flexible Sigmoidoscopy/Colonoscopy are included in the table in Section 11.2.1 below. Colonoscopies are recommended as per NICE guidance for colorectal follow-up.[25] Disease status for the assessment of overall survival and disease free survival will be collected at 10 years using the surgical clinics/GP patient records

The timing, indication and **interpretation of biopsies** are critically important in the context of this trial. The details for preparing the biopsy specimen and histopathological analysis are included in the Histopathology protocol in Appendix 5. Blind biopsies are discouraged, i.e. if there is no abnormality on sigmoidoscopy/ colonoscopy, then we do not recommend biopsies directed at the mucosa once occupied by the tumour. Biopsies are discouraged unless: i) an area of residual disease is visualised at (and no earlier than) 6-month Sigmoidoscopy; ii) a clinically suspicious area develops/re-appears at Sigmoidoscopy/colonoscopy; iii) MR changes raise the suspicion of re-growth or relapse.

Patients with evidence of clinical or radiological local re-growth or pelvic relapse will be referred immediately to the surgical team for urgent biopsy. Radiological, Endoscopic or Clinical changes that suggest a clinically suspicion of re-growth or pelvic relapse require urgent discussion at a colorectal MDT. If not yet performed an urgent up-to-date MRI (< 1 month) is required. If urgent salvage surgery is recommended, a PET/CT scan will be performed routinely pre-operatively to out rule occult metastatic disease as recommended by National guidelines.

Subjects with metastatic disease detected during follow-up will be treated according to the local MDT recommendation. If the MDT decides that palliative care is required, the patient will discontinue trial treatment and be classed as 'end of study' but still followed up (by completion of the **ANNUAL FOLLOW-UP Form)**, with analysis for the primary endpoint assessed on an intention to treat basis.

Patients with evidence of clinical or radiological local re-growth or pelvic relapse must be treated at least as urgently as a primary rectal cancer by the surgical team. Refer to deferral of surgery and regrowth pathways in Section 8.

After surgery patients will receive the post-surgery follow-up as described in Section 11.1 above.

11.2.1 SURVEILLANCE SCHEDULE FOR PATIENTS WHO DEFER SURGERY

Time line from end of CRT	Visit window	Clinic out-patient appoint-ment [b]	Patient reported outcome measures [c]	Scans	Endoscopy	Completion of Case Report Forms[d]
**6 months** [ae]	± 1 month	X		MRI	Flex Sig	5B.INTERVENTION ARM POST-TREATMENT MRI<br>12 SURVEILLANCE
**9 months** [e]	± 1 month	X		MRI	Flex Sig	5B.INTERVENTION ARM POST-TREATMENT MRI<br>12. SURVEILLANCE
**1 year** [e]	± 1 month	X	EORTC QLQ-C30, LARS, EQ-5D	MRI<br>CT	Colonoscopy	5B.INTERVENTION ARM POST-TREATMENT MRI<br>10. TOXICITY<br>11. ANNUAL FOLLOW-UP<br>12. SURVEILLANCE<br>17. QUALITY OF LIFE
**1 year 3 months**	± 1 month	X				12. SURVEILLANCE
**1 year 6 months** [e]	± 1 month	X		MRI	Flex Sig	5B.INTERVENTION ARM POST-TREATMENT MRI<br>12. SURVEILLANCE
**1 year 9 months**	± 1 month	X				12. SURVEILLANCE
**2 years** [e]	± 1 month	X		MRI<br>CT	Flex Sig	5B.INTERVENTION ARM POST-TREATMENT MRI<br>11. ANNUAL FOLLOW-UP<br>12. SURVEILLANCE
**2 years 6 months** [e]	± 1 month	X				12. SURVEILLANCE
**3 years** [e]	± 2 months	X	EORTC QLQ-C30, LARS, EQ-5D	MRI<br>CT	Flex Sig	5B.INTERVENTION ARM POST-TREATMENT MRI<br>11. ANNUAL FOLLOW-UP<br>12.SURVEILLANCE<br>17. QUALITY OF LIFE
**3 years 6 months**	± 2 months	X				12. SURVEILLANCE
**4 years** [e]	± 2 months	X		MRI	Flex Sig	5B.INTERVENTION ARM POST-TREATMENT MRI<br>11. ANNUAL FOLLOW-UP<br>12. SURVEILLANCE
**4 years 6 months**	± 2 months	X				12. SURVEILLANCE
**5 years** [e]	± 3 months	X	EORTC QLQ-C30, LARS, EQ-5D	MRI	Colonoscopy	5B.INTERVENTION ARM POST-TREATMENT MRI<br>11. ANNUAL FOLLOW-UP<br>12. SURVEILLANCE<br>17. QUALITY OF LIFE

[a] **This visit should take place once the patient has completed chemotherapy.** It is recommended that a CT scan is also performed following the completion of chemotherapy as is usual practice.

[b] Each clinic out-patient appointment should include a digital rectal exam and CEA (tumour marker) blood test.

[c] EORTC QLQ-C30, LARS & EQ-5D are included in the QUALITY OF LIFE CRF.

EORTC QLQ-C30, European Organisation for Research and Treatment of Cancer Quality of Life questionnaire v3.

LARS, Low Anterior Resection Syndrome Score. EQ-5D, EuroQol Group EQ-5D Health Questionnaire.

[d] A SURVEILLANCE Form should be completed at the time of a confirmed or equivocal diagnosis of regrowth. If a patient has an unsalveagable regrowth (see definition in Section 11.3) then this should be recorded on the ADVERSE EVENT Form in addition to the SURVEILLANCE Form.

[e] If patient has consented to the additional blood sample collection for research (Section 10.4, circulating tumour DNA and markers of cell proliferation and apoptosis) then these samples should be collected during the clinic out-patient appointment, ideally at the same time as the routine blood tests are performed.

Flex sig: flexible sigmoidoscopy.

11.3 DEFINITIONS REQUIRED FOR FOLLOW-UP

Complete response is defined as undetectable disease at the site of the primary tumour following pre-operative chemotherapy and/or radiotherapy. A pre-fix to denote the mode of assessment must also be included - clinical (c), MRI (mr) and pathology (p).

Disease recurrence is defined as either evidence of loco-regional recurrence or established metastatic disease. The time to the event is measured from the start of chemoradiotherapy to the date of the diagnostic investigation or MDT.

Regrowth is defined as the first recorded evidence of disease at the site of the primary tumour, in a patient with previous investigations suggesting an apparent complete response or undetectable disease (mrTRG1&2). The terms 'early regrowth' and 'late regrowth' will not be used in the trial.

Unsalvageable regrowth is defined as a tumour with radiological evidence of a good response that is initially managed by deferral of surgery but progresses. The extent of progression means that pCRM involvement becomes inevitable and so surgery is not performed, or pCRM involvement occurs when surgery is attempted.

Loco-regional recurrence (one of):

- Clinical recurrence with biopsy proof of recurrence
- CEA elevation and enlarging or new mass (includes pelvic, nodal and peritoneal disease). Biopsy confirmation is contraindicated or not feasible
- CEA non-secretors: enlarging or new mass (includes pelvic, nodal and peritoneal disease). Biopsy confirmation is contraindicated or not feasible (with or without PET/CT confirmation)

Distant Metastases (one of):

- This is defined as any confirmed evidence of disease above the L5/S1 junction
- Clinical recurrence with biopsy proof of recurrence
- CEA elevation and evidence of distant metastases. Biopsy confirmation is contraindicated or not feasible

- CEA non-secretors: evidence of distant metastases. Biopsy confirmation is contraindicated or not feasible (with or without PET/CT confirmation). Initial report to Trials Unit should define this as SUSPECTED. A further report 6 months later should define this as CONFIRMED if supported by MDT review.

Inoperable Disease (one of):

- MDT decision that the local extent of disease on the post CRT pelvic MRI is not resectable
- At laparotomy the local extent of disease is not resectable
- Incomplete resection of tumour at laparotomy after pre-operative CRT with biopsy proof of residual disease. (Lack of biopsy proof in this situation would constitute "suspected" persisting disease

Suspected Regrowth: When clinical assessment, CEA values, Radiological investigations and Endoscopic assessment lead to an equivocal diagnosis of regrowth, please indicate this in the allocated box on the **SURVEILLANCE Form indicating an equivocal diagnosis** and submit this form to the trial office.

11.4 COMPLIANCE WITH THE PROTOCOL

The investigators and sponsor will agree to implement the study protocol as written. The study will be performed in accordance with the clinical trials regulations, the Declaration of Helsinki and the principles of GCP.

11.5 DEFINITION OF END OF TRIAL

The end of the trial is defined as the last patient's last visit.

12 DATA MANAGEMENT

12.1 COMPLETING CASE REPORT FORMS

Trial data will be recorded on version controlled Case Report Forms (CRF pack) by staff included on the site Delegation Log who are authorised to make entries and/or corrections on CRFs. Data entered onto CRFs must reflect source data at site. Source documents are original documents, data, and records from which the patient's CRF data are obtained. These include but are not limited to hospital records, clinical and hospital charts, laboratory and pharmacy records, images and correspondence. Case report entries may be considered source data if the CRF is the site of the original recording i.e. there is no other written or electronic record of data. In this situation the CRFs may be used as source documents for the purpose of this trial. The PI is responsible for the accuracy of all data reported in the CRF.

The list of CRFs required during the trial and the completion timelines are included at the front of the CRF pack and included in the Assessment schedules above. Once completed these should be sent to **the TRIGGER Trial Office** at the Royal Marsden and the original retained at site.

12.2 CORRECTIONS TO CASE REPORT FORMS

Any corrections made to a CRF must be made by drawing a single line through the incorrect item ensuring that the previous entry is not obscured. Each correction must be dated and initialed. The amended CRF must be sent to the TRIGGER Trial Office and a copy retained at site.

12.3 MISSING DATA

To avoid the need for unnecessary data queries CRFs must be checked at site to ensure there are no blank data fields before sending them to the TRIGGER Trial Office. Please use the following acronyms as appropriate:

- **ND** - for not done - when data are unavailable because a measure has not been taken or a test not performed.
- **NA** – for not applicable – if a measure or test was not required at a particular time the form relates to.
- **NK** – for not known – when data are unknown (only use if every effort has been made to obtain data).

12.4 DATA QUERIES

Data arriving at the TRIGGER Trial Office will be checked for completeness, accuracy and consistency. Queries on incomplete, inaccurate or inconsistent data will be sent to the appropriate contact at the study site. When responding to a query, site staff should attach an amended copy of the CRF as appropriate and a copy retained at site. All amendments must be initialled and dated.

12.5 SUBMISSION TIMELINES

All CRFs must be completed at site and returned to the TRIGGER Trial Office as soon as possible after the patient visit and within one month of the patient being seen.

12.6 DATA COLLECTION AND RETENTION

The investigator and trial staff are responsible for maintaining a comprehensive, centralised and secure filing system of all trial-related documentation, suitable for trial-related monitoring, audits or inspection at any time by representatives from the sponsor and/or regulatory authorities. Trial documentation includes original source documents, patient files containing completed CRFs and informed consent forms, patient identification list and site investigator site file.

No trial documentation should be destroyed without prior written agreement between the sponsor and the investigator. Should the investigator wish to assign the study records to another party or move them to another location, he/she must notify the sponsor in writing of the new responsible person and/or location.

The Sponsor should ensure that all trial related documentation be retained for the maximum period of time as specified in the UK Clinical Trial Regulations.

12.7 TRIAL DATABASE

Patients' data will be documented on a trial specific CRF. Only the Investigator, and those personnel who have completed the Study Team Delegation Log as authorised by the PI, should enter or change data on the CRF. The data will be entered in a clinical trials database (Macro V4). Data entry will be carried out by appropriately trained personnel. Data clarification queries will be raised centrally by the trial manager and sent to each participating centre for resolution. Access to the study database is restricted to the appropriate data managers and statisticians.

13. TRIAL MONITORING AND OVERSIGHT

Participating sites and Principal Investigators must agree to allow trial-related on-site monitoring, including Sponsor audits and regulatory inspections by providing direct access to source data/documents as required. Patients are informed of this in the patient information sheet and are asked to consent to their medical notes being reviewed by appropriate member(s) from the TRIGGER Trial Office, or from regulatory authorities or from the NHS Trust on the consent form.

13.1 SELF-ASSESSMENT MONITORING

Participating sites will be requested to complete a Trial Self-Assessment Monitoring form periodically, at a frequency detailed in the Trial Monitoring Plan. This report may include, but is not limited to:

- Investigator Site File document review
- Pharmacy Site File document review
- Recruitment status
- Review of Informed Consent Forms
- Pharmacovigilance review

Responses will be reviewed at the TRIGGER Trial Office to identify any areas of non-compliance/fraud and to indicate training needs. Findings may trigger an on-site monitoring visit.

13.2 CENTRAL MONITORING

Site requirements for the purpose of central monitoring are summarised in the table below.

Site responsibility	Frequency	Central Monitoring
Send copy of delegation log	Every 3 months and every time the log is updated (addition or removal of trial staff, change in responsibilities)	To confirm adequate number of staff and delegated responsibilities appropriate. Confirm only staff with delegated responsibility are taking informed consent.
Send copy of patient eligibility checklist (included in REGISTRATION CRF)	At the time of patient registration	To confirm eligibility
Send copy of patient consent form at registration and randomisation	At registration and randomisation	Verify existence of patient
Send copies of screening, registration and randomisation logs.	Every 3 months.	To check for consistency and completeness and evaluate screen failure rate and reasons for non-inclusion at each stage.
Send Case Report Forms*	Each time a form is completed.	To allow checks for missing and unusual values and consistency over time, and generate data queries as appropriate. To ensure data is entered onto trial database in timely fashion.
*Data stored at the TRIGGER Trial Office will be checked for missing or unusual values (range checks) and checked for consistency over time. If any problems are identified data queries will be issued to the site. Sites are required to resolve any queries and update the relevant CRF as required. All changes must be initialled and dated. The amended version must be sent to the TRIGGER Trial Office and a copy retained at site.		

13.3 NON-COMPLIANCE/'FOR CAUSE' ON-SITE MONITORING

On-site monitoring visits may be scheduled where there is evidence or suspicion of non-compliance at a site with important aspect(s) of the trial protocol/GCP requirements. Sites will be sent a letter in advance outlining the reason(s) for the visit, a list of the documents that are to be reviewed, interviews that will be conducted, planned inspections of the facilities, who will be performing the visit and when the visit will likely occur.

Following a monitoring visit, the Trial Manager will provide a report to the site, which will summarise the documents reviewed and a statement of findings, deviations, deficiencies, conclusions, actions taken and actions required. The Principal Investigator at each site will be responsible for ensuring that monitoring findings are addressed (this may be delegated to an appropriate member of staff).

The TRIGGER Trial Working Group will assess whether it is appropriate for the site to continue participation in the trial and whether the incident(s) constitute a serious breach. See Appendix 1 (Incident Reporting and Serious Breaches) for details.

13.4 OVERSIGHT COMMITTEES

13.4.1 TRIAL MANAGEMENT GROUP (TMG)

The TMG will include the Chief Investigator, the Royal Marsden Hospital PI and CTIMP lead, the Principal Clinical Coordinator/Investigator, clinicians and experts from relevant specialities and trial staff from the TRIGGER Trial office (list of members included on page iv). The TMG will be responsible for overseeing the trial. The group will meet regularly (at least 4 times per year – with greater frequency at the start of the study) and will send updates to Principal Investigators (via newsletters and/or at Investigator Meetings).

The TMG will agree protocol amendments on behalf of the PIs prior to submission to the REC and MHRA. All PIs will be kept informed of substantial amendments through their nominated responsible individuals.

13.4.2 INDEPENDENT DATA MONITORING AND ETHICS COMMITTEE (DMEC)

The role of the DMEC is to provide independent advice on data and safety aspects of the trial. During the study, interim analyses of safety and outcome data will be supplied, in strict confidence, to an independent Data Monitoring and Ethics Committee (DMEC) along with any other analyses that the committee may request.

The DMEC will meet annually, or more frequently if considered appropriate, and will advise the chair of the TMG if, in their view, the randomised comparison in TRIGGER has provided both (a) “proof beyond reasonable doubt” that for all, or for some types of patient, treatment mrTRG directed management is clearly indicated or clearly contraindicated in terms of a net difference in the main outcome measures, and (b) evidence that might reasonably be expected to influence the patient management by many clinicians.

The TMG can then decide whether to modify the study protocol. Unless this happens, however, the TMG, the collaborators and all of the central administrative staff (except the statisticians who supply the confidential analyses) will not be informed of the DMEC analysis.

If the clinical coordinators are unable to resolve any concern satisfactorily, collaborators, and all others associated with the study, may write via the TRIGGER trial office to the chair of the DMEC, drawing attention to concerns about the possibility of particular side-effects, or of particular categories of patient requiring special study, or about any other matters thought relevant.

13.4.3 ROLE OF THE ROYAL MARSDEN NHS FOUNDATION TRUST

The TRIGGER Trial Office at the Royal Marsden will be responsible for the day to day coordination and management of the trial and will act as custodian of the data generated in the trial. The Royal Marsden NHS FOUNDATION TRUST, as sponsor, is responsible for all duties relating to pharmacovigilance which are conducted in accordance with Appendix 1 (Safety Monitoring Procedures).

14. STATISTICAL ANALYSIS AND SAMPLE SIZE

14.1 SAMPLE SIZE

14.1.1 PHASE III STUDY SAMPLE SIZE

A detailed sample size calculation will be included in the phase III protocol. However the expected sample size is estimated here in order to calculate the required recruitment rate, which is the primary endpoint of this protocol.

It is anticipated that the phase III trial will be designed to detect an improvement in 3 year DFS in the intention to treat population from 74% to 82% (i.e. a hazard ratio of 0.66) with 80% power and a 5% 2-sided level of statistical significance. This would require 633 patients (422 in the intervention arm and 211 in the control arm based on 2:1 allocation ratio) to be recruited over 3 years with at least 3 years follow up to observe the 184 events required. The final sample size for the phase III trial may differ depending on a number of factors including results of the feasibility trial.

14.1.2 FEASIBILITY STUDY SAMPLE SIZE

In this feasibility study a type II error would result in the cancellation of the planned phase III study when the recruitment rate was in fact sufficient to support such a study. We wish to minimise the probability of this occurring and so have set the chance of a type II error (B) at 5% and the resulting power (1-B) at 95%. A type I error would result in the planned phase III study going ahead when the true recruitment rate is poor. We would accept a 10% chance of this occurring and so have set the significance threshold (alpha) at 0.1.

The sample size for the primary endpoint of the feasibility study can be worked out as follows:

Recruitment of at least 633 patients to the phase III trial is planned to take place over a total of 3 years, and should take at most no more than 5 years. Hence the recruitment rate is expected to be 11 patients per month and will need to be no less than 5-6 patients per month (5/month results in total time of 6.5 years,

6/month results in 5.5 years total). In a similar multicentre trial sponsored by RM (6 vs. 12), recruitment rates over time were as follows:

Table: Recruitment rate in previous multi-centre trial (6 vs. 12)

Date of TMG	N centres open	Rate per month from last TMG to current	Rate per month per centre
Sept 2011	8	2	0.3
Dec 2011	9	6	0.7
March 2012	10	7.7	0.8
July 2012	12	7.3	0.6
December 2012	15	4.6	0.3

In order to calculate the required sample size, we ran a simulation in which a set of observations from a Poisson distribution with mean 11 were generated. A Chi-squared goodness of fit test of the null hypothesis that these observations were from a Poisson distribution with mean 6 was performed, and rejected if the resulting p-value was equal or less than 0.1. Using a total of 4 observations resulted in power between 95 and 96% with 10,000 replicates. In order to allow enough time for sites to open and collect sufficient data to allow a preliminary assessment of the safety of the deferral of surgery approach and the safety and acceptability of additional pre-operative chemotherapy, the maximum feasibility recruitment period will be 24 months. The primary hypothesis will be tested based on the average rate of recruitment in the four months following the activation of the 12th participating site, or the average rate of recruitment in the final four months of the feasibility recruitment period, whichever is earlier.

14. 2 ANALYSIS METHODS

14.2.1 PRIMARY ENDPOINT

The primary endpoint of this study is the total numbers of patients randomised per month, during the last 4 months of recruitment. A chi– squared goodness of fit test with one –sided alpha of 0.1 will be used to test the null hypothesis that the expected number of patient recruitment per month is equal to 6. Rejection of the null hypothesis would suggest that recruitment levels are adequate to support the proposed phase III study with sample size and length of recruitment as described in this protocol. However if the null hypothesis is not rejected then exact recruitment rates by time for each centre will be stated, and any known reasons for low recruitment investigated. After taking these results into consideration it may still be deemed possible to proceed with a phase III study if the design is amended accordingly. The primary endpoint for the feasibility study will be stated in full in the phase III protocol before seeking final approval.

14.2.2 SECONDARY ENDPOINTS

All secondary endpoints will be summarised using descriptive statistics, and will be used to inform the design of the proposed phase III study. The following limited number of endpoints will be summarised by arm, and within arm by patients grouped by mrTRG 1-2 vs. 3-5. Exploratory comparison between arms will be carried out using Fisher's exact test for categorical variables (pCRM rate, proportion with unsalveagable regrowth, proportion of patients with any one or more grade 3-5 toxicity). Quality of surgery and surgical morbidity (maximum Clavien-Dindo grade per patient) will be compared between arms using a Mann-Whitney test. No other formal statistical comparison will be made between arms or mrTRG subgroups for endpoints not listed below.

Control arm versus Intervention arm: all patients

- The pCRM rate will be compared between the control and investigation arms.
- The strength of agreement of the reported mrTRG will tested for the site radiologist versus the central radiologist. An MRI training dataset will be used prior to site initiation and a kappa agreement of 0.7 or greater will be required for the site radiologist to be eligible to recruit to the study. The agreement between the recruiting radiologists and central radiologist scored mrTRG grades for scans acquired during the study for patients randomised to the intervention arm will also be assessed retrospectively at the end of the feasibility trial. The agreement between the recruiting radiologists and central radiologist scored mrTRG grades for scans acquired during the study for patients randomised to the intervention arm will also be assessed retrospectively at the end of the feasibility trial.
- Early (30 day) and late (up to 12 months) surgical morbidity will be reported according to the Clavien-Dindo classification
- Quality of surgery will be determined by using the mesorectal grading system and a similar system will be used for grading the quality of APE specimens (Pathology appendix).[30,31] Outcomes will be compared between the control and intervention arms on an intention to treat basis.

Control arm versus Intervention arm: good responders (mrTRG1&2)

- The pCRM involvement rate in the good responders of the control versus intervention arm
- The proportion of patients with unsalvageable regrowth (defined in section 11.3). Greater than 5% unsalvageable regrowth may lead to premature closure of the trial or decision not to proceed to the phase III study.

Control arm versus Intervention arm: poor responders (mrTRG3-5)

- Acute toxicity will be recorded to CTCAE v4.0 protocol. The grade 3- 5 outcomes will be compared between the control and intervention arms on an intention to treat basis.

The preliminary biomarker analysis using proliferative and apoptotic molecular and genomic markers will be hypothesis generating work with descriptive analysis only.

A detailed statistical analysis plan including methods and timing for all endpoints will be written and agreed before commencing any analysis.

Additional endpoints are planned for the phase III trial, including but not limited to quality of life, overall and progression free survival, and data for these endpoints will be collected from patients in the feasibility trial so that they can be included in a pooled analysis of data from the feasibility and phase III trials. Plans for this pooled analysis will be described in full in the proposed phase III protocol. If the planned phase III trial does not commence due to poor recruitment in the feasibility phase, an amendment to this protocol shall be submitted detailing the proposed analysis of this additional data as a part of the feasibility trial.

15. ORGANISATION

To ensure the smooth running of TRIGGER and to minimise the overall procedural workload, it is proposed that each centre should designate individuals who would be chiefly responsible for local coordination of the surgical and administrative aspects of TRIGGER. The TRIGGER Trial Office will provide as much assistance as they can to local co-ordinators and investigators in obtaining Trust approval in each centre and helping resolve any local problems that may be encountered.

15.1 FUNDING

The TRIGGER feasibility study will be funded by a pump-primed grant from the Pelican Cancer Foundation with agreed adoption to NIHR Clinical Research Network portfolio. This will have benefits in coordination of research efforts, dissemination of trial information and local support for investigators. Inclusion of patients in the TRIGGER trial should incur only minimal additional costs for participating hospitals. Follow-up appointments can be co-ordinated to fit in with the patient’s existing follow-up schedule. Funding for the phase III trial will be applied for from the NIHR.

IMAGING

Hospital trusts will not be reimbursed for the staging and restaging MRI(s) or for follow up imaging associated with routine clinical care.

Detailed information about pathways for sending anonymised MRIs scans back to the central office for interpretation will be provided on a site by site basis. For hospitals with PACS links, there will be provision for anonymised electronic transfer. For centres without these links, there will be provision for secure postage of a CD back to the TRIGGER Trial Office at the Royal Marsden.

15.2 PRINCIPAL INVESTIGATOR AT EACH CENTRE

Each TRIGGER centre should nominate a Consultant Colorectal Surgeon or Oncologist to act as the local Principal Investigator and bear responsibility for the conduct of research at their centre. The responsibilities of the local Principal Investigator (PI) will be to ensure that all medical and nursing staff involved in the care of potential patients are well informed about the trial and trained in trial procedures, including obtaining informed consent. Close collaboration between all clinical teams is particularly important in order that patients for whom the TRIGGER trial is an option can be identified sufficiently early for entry.

The local PI will also be responsible for ensuring adherence to the protocol. The local PI should liaise with the Trial Manager on logistic and administrative matters connected with the trial.

15.3 CENTRAL COORDINATION

The Trial Office at the Royal Marsden is responsible for providing the following trial materials:

- The Investigator Site File, containing all documentation required to define the involvement of the centre in the trial.
- An Investigators' folder containing printed materials, such as the CRFs, participant information sheets, consent forms and GP letters.
- An Imaging Manual including details relating to scan acquisition and scan transfer.
- A Laboratory Manual

Additional supplies of any printed material can be obtained on request. The Trial Office also provides the central randomisation service and is responsible for collection and checking of data and for reporting of serious adverse events to the sponsor and regulatory authorities on behalf of the Chief Investigator and for any interim and final data analyses. The Trial Office will help resolve any local problems that may be encountered in trial participation.

15.4 CLINICAL QUERIES

During office hours, the relevant member of the trial team (see page iii) may be contacted about clinical queries.

15.5 PUBLICATIONS

A meeting will be held at the end of the trial to allow discussion of the main results among the collaborators prior to publication. The success of the trial depends entirely on the wholehearted collaboration of a large number of doctors, nurses and researchers. Centres will not be permitted to publish data obtained from participants in the TRIGGER trial that uses trial outcome measures without discussion with the Chief Investigator and the Trial Steering Committee.

16. RESEARCH GOVERNANCE

The conduct of the trial will be in accordance with the clinical trials regulations, the principles of Good Clinical Practice and the Declaration of Helsinki.

SPONSOR

National sponsorship will be provided by the Royal Marsden NHS Foundation Trust upon signing of the Clinical Study Site Agreement with each trial site.

CLINICAL TRIALS UNIT

Data from this trial will be handled by the Royal Marsden. This is a full-time research facility dedicated to, and with substantial experience in, the design and conduct of randomised clinical trials. The Royal Marsden recognises the responsibilities of a data management centre with respect to the ethical practice of research and the adequate protection of human subjects.

CONFIDENTIALITY OF PERSONAL DATA

The trial will collect personal data about participants, and medical records will be reviewed for all patients and routine physical examinations will be performed. Participants will be informed that their trial data and information will be securely stored at the trial office at the Royal Marsden, and will be asked to consent to this. The Royal Marsden abides by the UK law Data Protection Act 1998. The data will be stored on a secure computer database, and all personal information obtained for the study will be held securely and treated as strictly confidential. Any data transferred outside of the Royal Marsden will be fully anonymised.

LONG-TERM STORAGE OF DATA

In line with Good Clinical Practice guidelines, all essential documentation and data will be retained for at least 25 years.

INDEMNITY

TRIGGER was developed by the MERCURY III study group and the Royal Marsden, and the feasibility study is being supported by The Pelican Cancer Foundation. The Royal Marsden NHS Foundation Trust is the trial Sponsor. The normal NHS indemnity liability arrangements for clinician initiated research will, therefore, operate. It should be noted, however, that negligent liability remains the responsibility of the hospital, whether or not a patient is part of a clinical trial, because of the duty of care that the hospital has for their patients.

17. ABBREVIATIONS AND DEFINITIONS:

APE	Abdominoperineal excision
CEA	Carcinoembryonic antigen – a tumour marker for colorectal cancer
COREC	Central Office for Research Ethics Committees
CRM	Circumferential Resection Margin
CRT	Chemoradiotherapy
CTCAE	Common Terminology Criteria for Adverse Events - describe the severity of organ toxicity for patients receiving cancer therapy. Toxicity is graded as mild (Grade 1), moderate (Grade 2), severe (Grade 3), or life-threatening (Grade 4), with specific parameters according to the organ system involved. Death (Grade 5) is used for some of the criteria to denote a fatality.
EORTC	The European Organisation for Research and Treatment of Cancer
FDG-PET	F^{18}Fluorodeoxyglucose-positron emission tomography – a measure of tissue glucose uptake, this correlates with tissue metabolism in certain types of tissue.
GCP	Good Clinical Practice - is a quality standard for research that is defined in the clinical trials regulations.
LAR	Low Anterior Resection
Low Rectal Cancer	Lower edge of the tumour < 6cm from the anal verge
MedDRA	Medical Dictionary for Regulatory Activities
MRI	Magnetic Resonance Imaging
mrTRG	Magnetic Resonance Tumour Regression Grade
NBOCAP	National Bowel Cancer Audit Programme
pCRM involvement	pCRM – pathological circumferential resection margin. pCRM involvement is defined as tumour ≤ 1mm from the circumferential resection margin at histopathological analysis. For the purpose of the trial, if a patient in the deferral of surgery arm does not undergo an operation and remains 'regrowth' free at 3-year follow-up this will also be defined as pCRM negative.
QoL	Quality of Life
RCT	Randomised Control Trial
REC	Research Ethics Committee
RTOG	Radiation Therapy Oncology Group
TRIGGER	Magnetic Resonance Tumour Regression Grade (mrTRG) As A Novel Biomarker To Stratify The Management Of Good And Poor Responders To Chemoradiotherapy: A Rectal Cancer Multicentre Randomised Control Trial
yMRI	The post chemoradiotherapy MRI scan. This scan is compared with the baseline MRI in order to perform the mrTRG.
QALY	Quality Adjusted Life Years
WHO performance status	**(Grade 0 -5)**<br>0 = Able to carry out normal activity without restriction; 1 = Restricted in physical strenuous activity but ambulatory and able to carry out light work; 2 = Ambulatory and capable of self-care but unable to carry out any work; up and about more than 50% of working hours; 3=Capable only of limited self-care; confined to bed or chair ore than 50% of waking hours; 4 = Completely disabled; cannot carry out any self-care; totally confined to bed or chair; 5 = Dead

18. REFERENCES

1. Fokas E, Liersch T, Fietkau R, et al. Tumor Regression Grading After Preoperative Chemoradiotherapy for Locally Advanced Rectal Carcinoma Revisited: Updated Results of the CAO/ARO/AIO-94 Trial. *Journal of clinical oncology : official journal of the American Society of Clinical Oncology* 2014.
2. Capirci C, Valentini V, Cionini L, et al. Prognostic value of pathologic complete response after neoadjuvant therapy in locally advanced rectal cancer: long-term analysis of 566 ypCR patients. *International journal of radiation oncology, biology, physics* 2008; **72**(1): 99-107.
3. Valentini V, Minsky BD. Tumor Regression Grading in Rectal Cancer: Is It Time to Move Forward? *Journal of clinical oncology : official journal of the American Society of Clinical Oncology* 2014.
4. The Beyond T. M. E. Collaborative. Consensus statement on the multidisciplinary management of patients with recurrent and primary rectal cancer beyond total mesorectal excision planes. *The British journal of surgery* 2013; **100**(8): E1-E33.
5. Patel UB, Blomqvist LK, Taylor F, et al. MRI after treatment of locally advanced rectal cancer: how to report tumor response--the MERCURY experience. *AJR American journal of roentgenology* 2012; **199**(4): W486-95.
6. Patel UB, Taylor F, Blomqvist L, et al. Magnetic resonance imaging-detected tumor response for locally advanced rectal cancer predicts survival outcomes: MERCURY experience. *Journal of clinical oncology : official journal of the American Society of Clinical Oncology* 2011; **29**(28): 3753-60.
7. Patel UB, Brown G, Rutten H, et al. Comparison of magnetic resonance imaging and histopathological response to chemoradiotherapy in locally advanced rectal cancer. *Annals of surgical oncology* 2012; **19**(9): 2842-52.
8. Scott N HJ, ACPGBI. National Bowel Cancer Audit Progress Report Tripartite Colorectal Meeting Birmingham, 2014.
9. Emmertsen KJ, Laurberg S, Rectal Cancer Function Study G. Impact of bowel dysfunction on quality of life after sphincter-preserving resection for rectal cancer. *Br J Surg* 2013; **100**(10): 1377-87.
10. Peeters KC, van de Velde CJ, Leer JW, et al. Late side effects of short-course preoperative radiotherapy combined with total mesorectal excision for rectal cancer: increased bowel dysfunction in irradiated patients--a Dutch colorectal cancer group study. *J Clin Oncol* 2005; **23**(25): 6199-206.
11. Patient Forum. Low rectal cancer - deferral of surgery? 2014. http://coloncancersupport.colonclub.com/viewtopic.php?f=1&t=45894&sid=ded6e0a46cae3b43f34943d08f97f29c - p333961.
12. Habr-Gama A, Perez RO. Non-operative management of rectal cancer after neoadjuvant chemoradiation. *Br J Surg* 2009; **96**(2): 125-7.
13. Habr-Gama A, Perez RO, Nadalin W, et al. Operative versus nonoperative treatment for stage 0 distal rectal cancer following chemoradiation therapy: long-term results. *Ann Surg* 2004; **240**(4): 711-7; discussion 7-8.
14. Habr-Gama A, Gama-Rodrigues J, Sao Juliao GP, et al. Local Recurrence After Complete Clinical Response and Watch and Wait in Rectal Cancer After Neoadjuvant Chemoradiation: Impact of Salvage Therapy on Local Disease Control. *International journal of radiation oncology, biology, physics* 2014; **88**(4): 822-8.
15. Smith JD, Ruby JA, Goodman KA, et al. Nonoperative management of rectal cancer with complete clinical response after neoadjuvant therapy. *Ann Surg* 2012; **256**(6): 965-72.
16. Habr-Gama A, Sabbaga J, Gama-Rodrigues J, et al. Watch and wait approach following extended neoadjuvant chemoradiation for distal rectal cancer: are we getting closer to anal cancer management? *Dis Colon Rectum* 2013; **56**(10): 1109-17.
17. Maas M, Beets-Tan RG, Lambregts DM, et al. Wait-and-see policy for clinical complete responders after chemoradiation for rectal cancer. *Journal of clinical oncology : official journal of the American Society of Clinical Oncology* 2011; **29**(35): 4633-40.
18. Smith FM, Wiland H, Mace A, Pai RK, Kalady MF. Clinical criteria underestimate complete pathological response in rectal cancer treated with neoadjuvant chemoradiotherapy. *Diseases of the colon and rectum* 2014; **57**(3): 311-5.

19. Habr-Gama A, Perez RO. The surgical significance of residual mucosal abnormalities in rectal cancer following neoadjuvant chemoradiotherapy (Br J Surg 2012; 99: 993-1001). *The British journal of surgery* 2012; **99**(11): 1601; author reply -2.
20. Smith FM, Chang KH, Sheahan K, Hyland J, O'Connell PR, Winter DC. The surgical significance of residual mucosal abnormalities in rectal cancer following neoadjuvant chemoradiotherapy. *The British journal of surgery* 2012; **99**(7): 993-1001.
21. West N. The surgical significance of residual mucosal abnormalities in rectal cancer following neoadjuvant chemoradiotherapy (Br J Surg 2012; 99: 993-1001). *The British journal of surgery* 2012; **99**(7): 1001.
22. Heald RJ, Beets G, Carvalho C. Report from a consensus meeting: response to chemoradiotherapy in rectal cancer - predictor of cure and a crucial new choice for the patient: On behalf of the Champalimaud 2014 Faculty for 'Rectal cancer: when NOT to operate'. *Colorectal disease : the official journal of the Association of Coloproctology of Great Britain and Ireland* 2014; **16**(5): 334-7.
23. São Julião GP, Smith FM, Macklin CP, George ML, Wynn GR. Opinions have changed on the management of rectal cancer with a complete clinical response to neoadjuvant chemoradiotherapy. *Colorectal Disease* 2014; **16**(5): 392-4.
24. Nielsen LB, Wille-Jorgensen P. National and international guidelines for rectal cancer. *Colorectal Dis* 2014.
25. The diagnosis and management of colorectal cancer: NICE clinical guideline 131. 2011. http://www.nice.org.uk/guidance/CG131 (accessed 28th October 2014.
26. Garcia-Aguilar J, Smith DD, Avila K, Bergsland EK, Chu P, Krieg RM. Optimal timing of surgery after chemoradiation for advanced rectal cancer: preliminary results of a multicenter, nonrandomized phase II prospective trial. *Annals of surgery* 2011; **254**(1): 97-102.
27. Glynne-Jones R, Sebag-Montefiore D, Maughan TS, Falk SJ, McDonald AC. A phase I dose escalation study of continuous oral capecitabine in combination with oxaliplatin and pelvic radiation (XELOX-RT) in patients with locally advanced rectal cancer. *Ann Oncol* 2006; **17**(1): 50-6.
28. Rodel C, Liersch T, Hermann RM, et al. Multicenter phase II trial of chemoradiation with oxaliplatin for rectal cancer. *J Clin Oncol* 2007; **25**(1): 110-7.
29. Rodel C, Liersch T, Becker H, et al. Preoperative chemoradiotherapy and postoperative chemotherapy with fluorouracil and oxaliplatin versus fluorouracil alone in locally advanced rectal cancer: initial results of the German CAO/ARO/AIO-04 randomised phase 3 trial. *Lancet Oncol* 2012; **13**(7): 679-87.
30. Quirke P. The role of the pathologist. *European Journal of Cancer Supplements* 2005; **3**(3): 351-9.
31. Quirke P, Steele R, Monson J, et al. Effect of the plane of surgery achieved on local recurrence in patients with operable rectal cancer: a prospective study using data from the MRC CR07 and NCIC-CTG CO16 randomised clinical trial. *Lancet* 2009; **373**(9666): 821-8.
32. Moran BJ. Chapter 7: Total mesorectal excision for rectal cancer. In: Moran BJ, Heald RJ, eds. Manual of Total Mesorectal Excision CRC PressI Llc; 2013: 103-23.
33. West NP, Anderin C, Smith KJ, Holm T, Quirke P. Multicentre experience with extralevator abdominoperineal excision for low rectal cancer. *Br J Surg* 2010; **97**(4): 588-99.
34. Holm T, Ljung A, Haggmark T, Jurell G, Lagergren J. Extended abdominoperineal resection with gluteus maximus flap reconstruction of the pelvic floor for rectal cancer. *The British journal of surgery* 2007; **94**(2): 232-8.
35. Holm T. Chapter 8: Abdominoperineal excision of the rectum. In: Moran BJ, Heald RJ, eds. Manual of Total Mesorectal Excision CRC PressI Llc; 2013: 124-39.
36. Shihab OC, Heald RJ, Holm T, et al. A pictorial description of extralevator abdominoperineal excision for low rectal cancer. *Colorectal Dis* 2012; **14**(10): e655-60.
37. Rahbari NN, Weitz J, Hohenberger W, et al. Definition and grading of anastomotic leakage following anterior resection of the rectum: a proposal by the International Study Group of Rectal Cancer. *Surgery* 2010; **147**(3): 339-51.
38. Clavien PA, Barkun J, de Oliveira ML, et al. The Clavien-Dindo classification of surgical complications: five-year experience. *Ann Surg* 2009; **250**(2): 187-96.

19. APPENDICES

APPENDIX 1: SAFETY MONITORING PROCEDURES

APPENDIX 2: CHEMOTHERAPY

APPENDIX 3: RADIOTHERAPY

APPENDIX 4: SURGERY & SURGICAL MORBIDITY

APPENDIX 5: HISTOPATHOLOGY

APPENDIX 6: MRI PROTOCOL

APPENDIX 7: TRANSLATIONAL BIOMARKER SUBSTUDY

APPENDIX 8: AMENDMENT HISTORY

APPENDIX 1: SAFETY MONITORING PROCEDURES

1. DEFINITIONS

1.1. ADVERSE EVENTS

An adverse event is any untoward medical occurrence in a patient or clinical investigation subject administered a pharmaceutical product and that does not necessarily have a causal relationship with this treatment.

Worsening of a pre-existing medical condition (e.g. diabetes, migraine headaches, gout) should be considered an adverse event if there is either an increase in severity, frequency, or duration of the condition or an association with significantly worse outcomes. Interventions for pre-existing conditions (e.g., elective cosmetic surgery) or medical procedures that were planned before study enrolment are not considered adverse events.

The investigator is responsible for reviewing laboratory test results and determining whether an abnormal value in an individual study patient represents a change from values before the study. In general, abnormal laboratory findings without clinical significance (based on the investigator's judgment) should not be recorded as adverse events; however, laboratory value changes requiring therapy or adjustment in prior therapy are considered adverse events.

1.2 ADVERSE REACTION (AR)

All untoward and unintended responses to chemotherapy or radiotherapy related to any dose administered to that subject. A causal relationship between a trial treatment and an adverse event is at least a reasonable possibility, i.e. the relationship cannot be ruled out.

1.3 UNEXPECTED ADVERSE REACTION (UAR)

An adverse reaction the nature and severity of which is not consistent with the information about the medicinal product in question set out in the Summary of Product Characteristics (SmPC) for that product.

1.4 SERIOUS ADVERSE EVENT (SAE)

Any adverse event, adverse reaction or unexpected adverse reaction which:

- Results in death
- Is life-threatening (the patient was at risk of death at the time of the event). This does not refer to an event that hypothetically might have caused death if it were more severe.
- Requires in-patient hospitalisation or prolongs existing hospitalisation.
- Results in persistent or significant disability/incapacity

- Consists of a congenital abnormality or birth defect

1.4.1 DISEASE-RELATED EVENTS

Elective hospital admissions for procedures related to the study treatment, for example administration of chemotherapy, or central line insertion, or elective admissions to simplify treatment are exempt from being reported as SAEs.

1.4.2 IMPORTANT MEDICAL EVENTS

Events that may not immediately life-threatening or result in death or hospitalisation but may jeopardise the patient or may require intervention to prevent one of the other outcomes listed in the definition of an SAE above should also be considered serious.

1.5 SERIOUS ADVERSE REACTION (SAR)

An SAE where a causal relationship between the investigational medicinal product (the systemic chemotherapy) and the SAE is at least a reasonable possibility, i.e., the relationship cannot be ruled out.

1.6 SUSPECTED UNEXPECTED SERIOUS ADVERSE REACTION (SUSAR)

A SUSAR is defined as an adverse reaction suspected to be related to the investigational medical product (the systemic chemotherapy) that is both unexpected and serious.

2 REPORTING PROCEDURES FOR ALL ADVERSE EVENTS

The investigator is responsible for ensuring that all adverse events (including SAEs) observed by the investigator or reported by patients from the date of the post-CRT MRI scan is performed until 30 days after the last dose of chemotherapy is administered are properly captured in the patients' medical records (source documents). Adverse events occurring in all arms of the trial should be reported with the same diligence so that bias is not introduced to the apparent incidence of adverse events observed in the intervention arm. In addition, the investigator is responsible for ensuring that, for those patients randomised, all adverse events captured on the patients' medical records (as specified above) are reported on the CRF.

NOTE: The rationale for commencing AE recording from the date of the post-CRT MRI rather than the date of randomization is to avoid capturing AEs during and shortly after the last two weeks of CRT i.e. those that could be possibly related to CRT rather than the IMP(s) (systemic chemotherapy) received as part of the trial, which patients do not receive until after the post-CRT MRI scan is performed 4-6 weeks after the completion of CRT.

As per assessment schedules residual toxicity will be assessed at the 12 month clinic visit.

In the TRIGGER trial, the seriousness, causality and expectedness of all adverse events will be assessed by the principal investigator (or delegate) at the participating site. Upon receipt at the trial management centre, all SAEs will be also reviewed by the Chief Investigator and CTIMP Lead at the Royal Marsden (or delegate).

ADVERSE EVENTS AND SERIOUS ADVERSE EVENTS RELATED TO SURGERY

Surgical adverse events and serious adverse events have been translated into complications. As surgery takes place during the Intervention Phase of the trial adverse events related to surgery should also be recorded on the **ADVERSE EVENTS Form**. Investigators should record on the **ADVERSE EVENT Form** whether the AE is related to systemic chemotherapy or surgery. A list of expected surgical complications is included in Section 2.3 below. Further information relating to early and late surgical complications will be captured on the **SURGICAL MORBIDITY Form**.

Please see section 2.1.4 regarding reporting of involved surgical resection margins (pCRM involvement).

For deferral of surgery the only adverse event of interest is **unsalvageable regrowth** (definition in section 11.3). This will be detected by the MDT during follow up. If it occurs this should be recorded on the **ADVERSE EVENT Form** and it is *definitely related* to deferral of surgery. A **SURVEILLANCE Form** should also be completed.

2.1 DEFINING THE ADVERSE EVENT

An adverse event term must be provided for each adverse event, preferably using the term listed in the Common Terminology Criteria for Adverse Events (CTCAE) v4.0, available online at:

http://evs.nci.nih.gov/ftp1/CTCAE/CTCAE_4.03_2010-06-14_QuickReference_5x7.pdf

2.1.1 DETERMINING ADVERSE EVENT SEVERITY

The NCI CTCAE version 4.0 must be used to grade each AE, and the worst grade recorded.

In those cases where the CTCAE criteria do not apply, severity should be coded according to the following criteria:

1 = Mild (awareness of sign or symptom, but easily tolerated)

2 = Moderate (discomfort enough to cause interference with normal daily activities)

3 = Severe (inability to perform normal daily activities)

4 = Life threatening (immediate risk of death from the reaction as it occurred)

5 = Fatal (the event resulted in death)

2.1.2 DETERMINING ADVERSE EVENT SERIOUSNESS

An adverse event is deemed serious if it satisfies any of the criteria in section 1.4 of this appendix above.

2.1.3 DETERMINING ADVERSE EVENT CAUSALITY

We are including adverse events for surgery and deferral of surgery (see section 2 above) as well as for the administration of systemic chemotherapy. For the IMP (systemic chemotherapy) causality should be assessed as follows:

Unrelated	The AE is definitely not associated with the systemic chemotherapy administered.
Unlikely to be related	The time association or the patient's clinical state is such that the systemic chemotherapy is not likely to have had an association with the observed effect.
Possibly related	Starts within a time related to the systemic chemotherapy administration and<br>A causal relationship between systemic chemotherapy and the AE is at least a reasonable possibility.
Probably related	Starts within a time related to the systemic chemotherapy administration and<br>Cannot be reasonably explained by known characteristics of the patient's clinical state.
Definitely related	Starts within a time related to the systemic chemotherapy administration and<br>No obvious alternative medical explanation.

Note: Drug-related refers to events assessed as possible, probable or definitely.

2.1.4 ADVERSE EVENTS OF SPECIAL INTEREST

These chemotherapy agents are established treatments for colorectal cancer, therefore there are no IMP-related events of special interest.

All involved surgical resection margins (pCRM involvement) should be treated as adverse events of special interest requiring expedited reporting to the trial office. Information relating to pCRM involvement is captured on the PATHOLOGY CRF, which should be completed at the time the surgical specimen is evaluated and reported. The PATHOLOGY CRF should then be sent to the TRIGGER Trial office as soon as it has been completed and signed and dated by the local Pathologist.

2.2 SERIOUS ADVERSE EVENT REPORTING PROCEDURES

With one exception, serious adverse events will be collected and recorded from the date of the post-CRT MRI scan is performed until 30 days after the last dose of chemotherapy is administered.

The only exception to this rule is the recording and reporting of involved surgical resection margins described in section 2.1.4 above.

2.2.1 EXEMPTIONS FROM SAE REPORT SUBMISSION

For this trial, the following events are exempt from requiring expedited notification on an SAE Report Form, but must be recorded in the relevant section(s) of the trial CRFs:

- any event that occurs after 30 days post last dose of chemotherapy that is not an ***AE of special interest*** listed in the section above or considered to be causally related to trial treatment
- Elective hospitalisation and surgery for treatment of locally advanced rectal carcinoma or its complications e.g. bowel obstruction
- Elective hospitalisation to simplify treatment or procedures e.g. central line insertion
- disease progression leading to hospitalization, or prolongation of hospitalization, or death as a result of disease progression

Please note that hospitalisation for elective treatment or palliative care does not qualify as an SAE.

2.3 DETERMINING ADVERSE EVENT EXPECTEDNESS

The following AEs are commonly associated with FOLFOX and CAPOX regimens or with rectal cancer surgery and will be considered expected:

Expected Adverse Events from Chemotherapy	
• Abdominal pain<br>• Allergic reaction<br>• Alopecia<br>• Anaemia<br>• Anorexia (Grade ≤ 3)<br>• Cardiac disorders (e.g. arrythmias, heart failure, ischemia)<br>• Constipation (Grade ≤3)<br>• Decreased platelet count<br>• Dehydration<br>• Diarrhoea<br>• Dysaesthesia<br>• Fatigue<br>• Febrile neutropenia	• Fever (Grade ≤ 3)<br>• Mucositis oral (Grade ≤ 3)<br>• Nausea<br>• Neuropathy – peripheral and sensory<br>• Neutrophil count decreased<br>• Neutrophil count decreased with infection<br>• Pain<br>• Paraesthesia<br>• Skin NOS (inc. Palmar-plantarerythrodysesthesia syndrome)<br>• Thromboembolic events (thrombosis and embolism)<br>• Vomiting
Expected Adverse Events from Surgery (termed complications)	
**Operative**<br>• Damage to organ/structure e.g. Bowel/Bladder/Ureter/Major Nerves<br>• Faecal contamination<br>• Haemorrhage<br>**Post-operative**	• Scrotal swelling<br>• Sepsis<br>• Sexual dysfunction<br>• Stoma prolapse/necrosis<br>• Stoke or Transient Ischaemic Event<br>• Subcutaneous emphysema<br>• Urinary dysfunction

• Acute renal failure<br>• Anastomotic leak<br>• Back pain<br>• Bowel Dysfunction<br>• Cholecystitis<br>• Delirium<br>• Disseminated intravascular coagulation<br>• Distal limb ischaemia/compartment syndrome<br>• Gastrointestinal fistula<br>• Gastrointestinal ischaemia/necrosis<br>• Gastrointestinal obstruction<br>• Gastrointestinal perforation<br>• Gastrointestinal stricture/stenosis<br>• Gastrointestinal ulceration<br>• Haemorrhage<br>• Hernia<br>• Ileus<br>• Intra-abdominal/pelvic abscess<br>• Metabolic acidosis<br>• Pancreatitis<br>• Post-operative peritonitis<br>• Pressure sore<br>• Pseudomembranous colitis	• Urinary retention<br>• Urinary tract infection<br>• Wound infection<br>• Wound dehiscence<br><br>**Cardiorespiratory** (May be operative or post-operative)<br>**Respiratory**, including<br>• Acute respiratory distress syndrome/respiratory failure<br>• Aspiration<br>• Atelectasis<br>• Bronchospasm<br>• Pleural effusion<br>• Pneumonia/chest infection<br>• Pulmonary embolus (or DVT)<br>**Cardiac**, including<br>• Arrhythmia<br>• Cardiac failure<br>• Ischaemic heart disease / myocardial infarction<br>• Cardio-respiratory arrest

For those events not described in the list above or in ***Events of Special Interest*** or ***Exemptions from SAE reporting*** please determine the expectedness and relatedness of an SAE by using the reference safety information included in Section 4.8 (Undesirable effects) of the summary of product characteristics (SmPC) for Capecitabine, 5-FU, Oxaliplatin and Folinic acid. The version of each of the SmPCs approved for this trial is shown in Section 9.4. An electronic and hard copy of each SmPC will be provided at the time of site initiation and updates sent as necessary.

2.4 NOTIFICATIONS

All SAEs other than those that are exempt from expedited notification (section 2.2.1 above) must be reported to the Sponsor on a completed SAE Report Form within 24 hours of discovery or notification of the event. These must be faxed to Clinical Trials Unit at the Royal Marsden on 0208 915 6762.

Please ensure that all completed SAE reports are faxed within 24 hours of becoming aware of the event.

SAE FAX NUMBER - 0208 915 6762

Follow-up of adverse events will continue until all the necessary safety data for the event has been gathered and until the drug-related AE or SAE has either resolved, returned to baseline or stabilised. For fatal or life-threatening cases, follow-up information should be reported as soon as possible. The investigator may be asked to provide follow-up information, discharge summaries, and extracts from medical records as necessary.

Should the investigator become aware of any drug-related SAEs after a patient has been withdrawn from study treatment these must also be reported within the timelines specified above.

The CTIMP Lead and CI (or in nominated individual in their absence) will be responsible for expedited reporting of all SUSARs to the MHRA, REC and Clinical R&D Office no later than 7 calendar days for fatal or life threatening SUSARs, or no later than 15 days for SUSARs which are nonfatal or non-life threatening. Follow up information for death/ life-threatening SUSARs should be provided within 8 days of initial report. For all other SUSARs, follow up report should be provided when the information becomes available.

The Sponsor is also responsible for all submissions of annual reports to the MHRA and REC. Development Safety Update Reports (DSURs) will be prepared in conjunction with the Clinical R&D office.

All SAEs will be reported to the Trial Management Group (TMG) approximately 6-monthly and to the Data Monitoring and Ethics Committee (DMEC) annually.

2.5 SAE REPORTING FLOWCHART

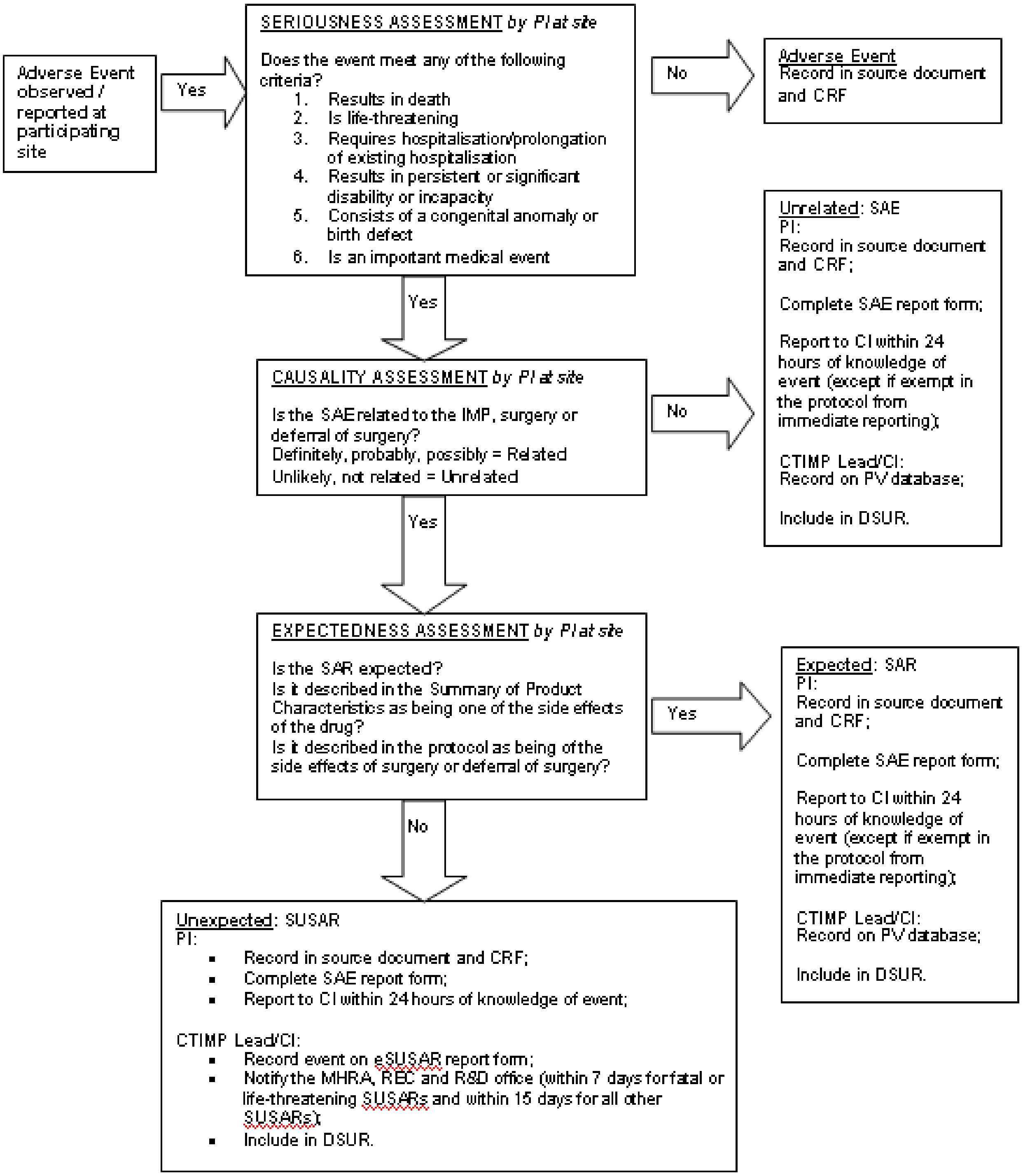

2.6 SERIOUS BREACHES AND URGENT SAFETY MEASURES

If a possible protocol violation and/or GCP breach has been identified during the trial, the CI will assess if the event affects the safety, physical or mental integrity of the trial subject or the scientific value of the study. Any possible serious breaches should be investigated further and the event should be reported to the Sponsor immediately. Where a serious breach has occurred, the Sponsor will ensure that the MHRA have been notified within 7 days of becoming aware of that breach.

The Sponsor or investigator may take appropriate urgent safety measures in order to protect the patient of a clinical trial against any immediate hazard to their health or safety. This includes procedures to protect patients from pandemics or infections that pose serious risk to human health. Urgent safety measures may be taken without prior authorisation from the MHRA and REC. The Sponsor must notify the MHRA and REC immediately and in writing within three days of such measures being taken.

2.7 CONTRACEPTION AND PREGNANCY REPORTING

The Investigator must make every effort to try and ensure that a clinical trial patient or a partner of a clinical trial patient does not become pregnant during the intervention phase of the trial. Contraceptive measures should be compliant with those described in the Summary of Product Characteristics for the IMPs used in this trial. This should be done as part of the consent process by explaining clearly to the patient the potential dangers of becoming pregnant and also providing each patient with information about highly effective birth control methods which can achieve a failure rate of less than 1% per year when used consistently and correctly. Such methods include:

- combined (estrogen and progestogen containing) hormonal contraception associated with inhibition of ovulation[1]
    - oral
    - intravaginal
    - transdermal
- progestogen-only hormonal contraception associated with inhibition of ovulation
    - oral
    - injectable
    - implantable
- intrauterine device (IUD)
- intrauterine hormone-releasing system (IUS)
- bilateral tubal occlusion
- vasectomised partner[2]
- sexual abstinence[3]

[1] Hormonal contraception may be susceptible to interaction with the IMP(s), which may reduce the efficacy of the contraception method. Please refer to the SmPC for each relevant IMP(s).
[2] Vasectomised partner is a highly effective birth control method provided that partner is the sole sexual partner of the women of child bearing potential trial participant and that the vasectomised partner has received medical assessment of the surgical success.
[3] sexual abstinence is considered a highly effective method only if defined as refraining from heterosexual intercourse during the entire period of risk associated with the study treatments. The reliability of sexual abstinence needs to be evaluated in relation to the duration of the intervention phase of this clinical trial and the preferred and usual lifestyle of the subject.

Contraceptives should be used from the time the patient joins the trial until completion of the intervention phase of the trial. Please refer to the SmPCs for each IMP for guidance of any continued requirement of contraceptive use when treatment with the IMP(s) is complete. It should be explained to each male patient that if his partner is pregnant or breast-feeding when he enters the trial, the patient should use barrier method contraception (condom plus spermicidal gel) to prevent the unborn baby or the baby being exposed to the IMP(s) (systemic chemotherapy).

The Investigator must ensure that all patients are aware at the start of a clinical trial of the importance of reporting all pregnancies (in themselves and their partners) that occur whilst being treated with the IMP(s). If a trial participant or a trial participant's partner does become pregnant during treatment with the IMP(s) it must be reported to the Chief Investigator (and the Sponsor) within 24 hours of the site staff becoming aware of it using the **PREGNANCY NOTIFICATION FORM**. Participants who become pregnant must be discontinued from trial treatment immediately. It is the Investigator's responsibility to obtain consent for follow-up from the patient or patient's partner. The Sponsor will follow-up all pregnancies for the pregnancy outcome via the Investigator and document on the **PREGNANCY NOTIFICATION FORM**.

The Investigator should offer counselling to the participant and/or the partner, and discuss the risks of continuing with the pregnancy and the possible effects on the foetus. With appropriate consent, monitoring of the participant and/or the partner and the baby should continue until the conclusion of the pregnancy. Pregnancy is not considered an AE unless a negative or consequential outcome is recorded for the mother or child/foetus. If the outcome meets the serious criteria, this would be considered an SAE and reported as such.

APPENDIX 2: CHEMOTHERAPY

1 OVERVIEW AND TIMING OF SYSTEMIC CHEMOTHERAPY

The patient and the medical oncologist may choose one of the chemotherapy regimens described below. Single agent systemic chemotherapy will be permitted if patient comorbidity precludes the use of concomitant oxaliplatin and is typically reserved for frail patients. The planned choice of systemic chemotherapy is a stratification variable for randomization, therefore the treatment decision should be made upfront, when the patient consents to trial registration. The same chemotherapy is offered to the control arm and both of the intervention arm subgroups but the timing will vary, as summarised in the Table 1 below:

The timing and number of cycles of chemotherapy according to regimen and randomization arm:

		**Intervention arm**	
**Systemic Chemotherapy Regimen [a]**	**Control Arm**	**Good Response sub-group [b]**	**Poor Response sub-group**
CAPOX (3 weekly cycle)	8 cycles post-op	8 cycles during deferral of surgery	4 cycles pre-op<br>4 cycles post-op
FOLFOX (2 weekly cycle)	12 cycles post-op	12 cycles during deferral of surgery	6 cycles pre-op<br>6 cycles post-op
Capecitabine	8 cycles post-op	8 cycles during deferral of surgery	4 cycles pre-op<br>4 cycles post-op
5-FU (Fluorouracil)	12 cycles post-op	12 cycles during deferral of surgery	6 cycles pre-op<br>6 cycles post-op
[a] For details relating to the preparation and administration of these drugs: see manufacturer's product information and refer to local guidelines.<br>[b] if the patient declines deferral of surgery post-operative systemic chemotherapy should be given i.e. as for control arm.			

- In this trial the above medications will be provided, prepared and administered by the Investigator site as per local guidelines. This appendix includes recommended chemotherapy regimens. Local sites are permitted to follow their standard practice for the dosage, administration and dose adjustment of systemic chemotherapy.
- Dose banding and capping as per local guidelines is permitted for CAPOX, FOLFOX and single agent therapy as long as the delivered dose falls within ± 7% of the calculated dose
- Local sites are permitted to follow their local guidance for the provision of supportive medication (e,g anti-emetics)
- Assessments including but not limited to routine blood tests, pregnancy tests, physical examinations including weight, ECGs, vital signs, CEA, and renal function should be performed as per local practice prior to and during systemic treatment.

1.1 CHEMOTHERAPY TREATMENT POST-SURGERY

Adjuvant chemotherapy should commence 4-12 weeks (ideally 6-8 weeks) after surgery. If this is delayed beyond 12 weeks, for example due to post-operative complications, the patient must be discussed with the lead centre (RMH) in order to determine if proceeding with adjuvant treatment is still appropriate.

1.2 CHEMOTHERAPY TREATMENT IN INTERVENTION ARM – GOOD RESPONDERS

If regrowth occurs while patient receives chemotherapy, patient should proceed to surgery and the pathology discussed at a MDT meeting to decide whether the remaining cycles of chemotherapy should be given post-operatively. A **SURVEILLANCE Form** should be completed.

1.3 CHEMOTHERAPY TREATMENT IN INTERVENTION ARM – POOR RESPONDERS

If patients have had a dose reduction during neoadjuvant (pre-operative) chemotherapy, the same dose as the finishing dose of the neoadjuvant chemotherapy should be started post- operatively.

1.4 CHANGES TO REGIMEN CHOICE DURING TREATMENT

It is not recommended to cross over regimens unless there is a compelling clinical reason to do so. However a mid-treatment switch is allowed in this protocol and this should be documented on the CHEMOTHERAPY CRF. NOTE: Be aware if crossing from FOLFOX to CAPOX, capecitabine may cause increased toxicity when given after recent folinic acid and a capecitabine-dose reduction is required.

If a patient switches to a therapy not included in this protocol then please contact the TRIGGER Trial Office to discuss with the Chief Investigator. The change in therapy should be recorded on the CHEMOTHERAPY CRF and a PROTOCOL DEVIATION Form should be completed.

2 RECOMMENDED CAPOX REGIMEN (3 WEEKLY CYCLE)

Drug	Dose	Day(s) in cycle	Administration
Oxaliplatin	130 mg/m^2	1	2-6 hr i.v. infusion*
Capecitabine	1700 mg/m^2/day	1-14	PO in two divided doses

* standard infusion to be given over 2hours. For patients who have a hypersensitivity reaction to oxaliplatin, it is recommended that infusion be given over 4hrs. For patients experiencing laryngopharyngeal spasm, it is recommended that infusion be given over 6hrs

Patients will be treated every 3 weeks (1 cycle) for a total of 8 cycles. As described in Table 1 patients randomized to the intervention arm in the poor response subgroup will receive 4 cycles pre-operatively and 4 cycles post-operatively.

Oxaliplatin 130mg/m^2 diluted in 250-500ml 5% dextrose will be given as an IV infusion over 2-6 hours. The maximum BSA for determining the dose of oxaliplatin and capecitabine is 2m^2.

Capecitabine will be administered as oral tablets: Capecitabine 1700mg/m2/day (850mg/m^2 twice daily) will be administered orally for 14 days followed by 7 days' rest.

Patients aged 75 or above will have a reduced starting dose: Capecitabine (1300mg/m^2/day) (650mg/m^2 twice daily) given orally divided into 2 split doses in the morning and evening for 14 days followed by 7 days' rest. Oxaliplatin 100mg/m^2 will be given every 3 weeks (the maximum BSA for determining the dose of oxaliplatin and capecitabine is 2m^2).

3 RECOMMENDED CAPECITABINE MONOTHERAPY REGIMEN (3 WEEKLY CYCLE)

Single agent capecitabine can be used if concomitant oxaliplatin is contraindicated.

Drug	Dose	Day(s) in cycle	Administration
Capecitabine	2000 mg/m^2/day<br>(1000mg/m2 twice daily)	1-14	PO in two divided doses

Patients aged over 75 will have a reduced starting dose: Capecitabine 1500mg/m^2/day (750mg/m^2 twice daily) given orally divided into 2 split doses in the morning and evening for 14 days followed by 7 days' rest.

Patients aged 80 or above will have a reduced starting dose: Capecitabine 1000mg/m^2/day (500mg/m^2 twice daily) given orally divided into 2 split doses in the morning and evening for 14 days followed by 7 days' rest.

4 RECOMMENDED FOLFOX REGIMEN (2 WEEKLY CYCLE)

Drug	Dose	Day(s) in cycle	Administration
Oxaliplatin	85 mg/m^2	1	2-6 hr i.v. infusion
Folinic acid [a]	400 mg/m^2	1	2 hr i.v. infusion concurrently with oxaliplatin
5-FU	400 mg/m^2<br>2400 mg/m^2	1<br>1-2	i.v. bolus<br>48 hr i.v. infusion [b]

[a] where local practice is to use levofolinic acid, then this is permitted at the locally approved dose

[b] or 46 hours if this is local practice.

Patients will be treated every 2 weeks (1 cycle) for a total of 12 cycles. As described in Table 1 patients randomized to the intervention arm in the poor response subgroup will receive 6 cycles pre-operatively and 6 cycles post-operatively.

5 RECOMMENDED 5-FU MONOTHERAPY REGIMEN (2 WEEKLY CYCLE)

5-FU/Folinic acid can be used if concomitant oxaliplatin is contraindicated

Drug	Dose	Day(s) in cycle	Administration
Folinic acid [a]	400 mg/m^2	1	2 hr i.v. infusion concurrently with oxaliplatin
5-FU	400 mg/m^2<br><br>2400 mg/m^2	1<br><br>1-2	i.v. bolus<br><br>48 hr i.v. infusion [b]

[a] where local practice is to use levofolinic acid, then this is permitted at the locally approved dose

[b] or 46 hours if this is local practice.

6 TOXICITY AND DOSE MODIFICATIONS

6.1 TOXICITY AND DOSE MODIFICATION NOTES

- Toxicity will be graded using CTCAE version 4.0.
- In general, the **maximum allowable toxicity related treatment delay is 3 weeks**. Any patient whose treatment is delayed for longer than 3 weeks due to treatment related toxicity should discontinue that phase of therapy. If it is clinically appropriate to do so, patients should proceed with the next phase of treatment within the study. However, due to the complex nature of the study treatment, it is not possible to pre-specify management for every situation. Therefore, if a scenario not addressed in this section arises, then the chief investigator should be contacted to discuss further management within the study.
- **For patients in the intervention arm poor response subgroup due to undergo surgery**: Where possible, and as long as it is still clinically appropriate to do so, patients should proceed to surgery, even if they are unable to complete pre-operative treatment due to toxicity when they have recovered adequately.
- If an unacceptable treatment delay due to toxicity from combination chemotherapy or oxaliplatin occurs, it would be acceptable for treatment to be completed with fluoropyrimidine alone, provided the treatment delaying toxicity is not considered to be due to the fluoropyrimidine itself. It is not permissible for patients to receive oxaliplatin without a fluoropyrimidine analog.
- If treatment is stopped due to patient choice or toxicity, subsequent treatment will be at the discretion of the treating clinician. This should be documented on the **CHEMOTHERAPY Form**. A **DISCONTINUATION OF TRIAL TREATMENT Form** should also be completed. All patients who withdraw from treatment should remain on follow-up within the trial**.**

Only toxicities deemed treatment-related by the investigator will require dose modifications. The recommended dose modifications of the study medications in the event of commonly known toxicities are outlined below. Clinical discretion should be used in the event of any serious toxicity not listed below and any other dose modifications may be performed at the discretion of the investigator. All concerns or uncertainties should be discussed with the Chief Investigator.

6.2 DOSE MODIFICATIONS

- Dose modification guidance is given for reference. Local sites are permitted to use their local standard dose modification protocols.

6.3 CAPOX/CAPECITABINE MONOTHERAPY

6.3.1 HAEMATOLOGICAL TOXICITY:

Grade 3 infection/fever associated with neutropenia (ANC<1) at any time on treatment requires a subsequent 25% dose reduction in oxaliplatin

Grade 4 infection/fever associated with neutropenia (ANC<1) at any time on treatment requires a subsequent 50% dose reduction in oxaliplatin

Asymptomatic neutropenia and thrombocytopenia on the day of treatment would require the following dose reductions for the CAPOX regimen:

Neutrophil count ($\times 10^9$/l)	CTCAE grade	Action
**≥1.0**	0-2	Full dose of all drugs
**0.5-0.9**	3	Stop capecitabine and delay oxaliplatin until recovery (e.g. 1 week later). Restart capecitabine at full dose. Reduce oxaliplatin by 25%
**<0.5**	4	Stop capecitabine and delay oxaliplatin until recovery (e.g. 1 week later). Restart capecitabine at 75% of the dose. Reduce oxaliplatin by 50%.
**Platelet count ($\times 10^9$/l)**	**CTCAE grade**	**Action**
**≥75**	0-1	Full dose of all drugs
**50-74**	2	Stop capecitabine and delay oxaliplatin until recovery (e.g. 1 week later). Restart both capecitabine and oxaliplatin at full dose.
**25-49**	3	Stop capecitabine and delay oxaliplatin until recovery (e.g. 1 week later). Restart capecitabine at 75% of the dose. Reduce oxaliplatin by 25%
**<25**	4	Stop capecitabine and delay oxaliplatin until recovery (e.g. 1 week later). Restart capecitabine at 75% of the dose. Reduce oxaliplatin by 50%

6.3.2 NON-HAEMATOLOGICAL TOXICITIES:

6.3.2.1 CAPECITABINE NON-HAEMATOLOGICAL TOXICITY:

Refer to table below for dose modifications for diarrhoea, nausea, vomiting, stomatitis

CTC Grade	1st Appearance Dose	2nd Appearance Dose	3rd Appearance Dose	4th Appearance Dose
**0-1**	100%	100%	100%	100%
**2**	delay* then 100%	delay* then 75%	delay* then 50%	discontinue
**3**	delay* then 75%	delay* then 50%	discontinue	discontinue
**4**	discontinue or delay* then 50%	discontinue	discontinue	discontinue

*interrupt treatment until resolved to Grade 0-1

All patients should be prescribed symptomatic treatment such as loperamide, sucralfate and emollients for diarrhoea, stomatitis and hand-foot syndrome respectively according to local practice.

Renal function: for creatinine clearance 30-50mls/min the dose of capecitabine should be reduced by 25%. If the dose has already been reduced by 25% for age, then an additional dose reduction for renal function is not required.

Liver function: capecitabine can cause an increase in bilirubin and liver transaminases. This is usually mild and of no clinical significance. However, if the bilirubin increases to >3x normal or AST/ALT increases to >2.5x normal the capecitabine should be withheld until the bilirubin <2.5 x ULN and ALT < 2.5 x ULN, at which point treatment can recommence without dose reduction.

6.3.2.2 OXALIPLATIN NON-HAEMATOLOGICAL TOXICITY

In Grade 3 or 4 stomatitis or diarrhoea reduce **Oxaliplatin** dose to 100mg/m$^{2.}$

Neurosensory toxicity:

The following dose reductions should be made:

Toxicity	≤ 7 days	> 7days	Persisting until next cycle
**Cold-related dysaesthesia**	No dose reduction	No dose reduction	Oxaliplatin until recovery then restart with 25% reduction of scheduled dose<br><br>Omit oxaliplatin if recurs
**Paraesthesia without pain**	No dose reduction	No dose reduction	Oxaliplatin until recovery then restart with 25% reduction of scheduled dose<br><br>Omit oxaliplatin if recurs
**Paraesthesia with pain**	No dose reduction	Delay oxaliplatin until recovery then restart with 25% reduction of scheduled dose	Omit Oxaliplatin
**Paraesthesia with functional impairment**	No dose reduction	Delay oxaliplatin until recovery then restart with 25% reduction of scheduled dose	Omit Oxaliplatin

6.4 DOSE MODIFICATION FOR FOLFOX TOXICITY/5-FU MONOTHERAPY

Toxicity<br>NCI CTC Grade	Therapeutic measures for toxicity	Initial Doses (mg/m^2/course)		
		5-FU bolus<br>400	5-FU Infusion<br>1200 x 2	Oxaliplatin<br>85
		Dose modifications (mg/m^2/course)		
**Neutrophils grade 3 or lower (<1.0x10^9/l)**	Delay until ≥1x10^9/L Consider G-CSF for G4 neutropenia / febrile neutropenia	400	1200	85
**Febrile neutropenia/ recurrent neutrophils grade 3 or lower (<1. 0x10^9/l), despite secondary prophylaxis**	Delay until ≥1x10^9/L	Omit bolus	1200 (consider reduction to 900)	85 (consider reduction to 70)
**Platelets grade 2 (50-75 x10^9/l)**	Delay until G1 (≥75x10^9/l)	400	1200	85
**Platelets grade 3/4 (<50x10^9/l)**	Delay until G1 (≥75x10^9/l). Consider emitting bolus 5-FU	300 (consider omission)	900	65
**Stomatitis or Diarrhoea grade 3**	Sucralfate, Codeine Loperamide, as indicated	300	900	85
**Stomatitis or Diarrhoea recurrent grade 3 or single grade 4**	Sucralfate, Codeine Loperamide, as indicated	300	900	70
**PPE grade 3**	Emolients	300	900	None
**Neuropathy**		None	None	*See section 6.3.2.2*

- In all cases treatment should be delayed until recovery of toxicity to grade 2 (except thrombocytopenia, where recovery should be to grade 1 (≥75x10^9/L)). Omission of the bolus of 5-FU can be considered in cases of neutropenia and thrombocytopenia.
- If a patient develops grade 4 neutropenia, delay chemotherapy until recovery to grade 2 (Neutrophils ≥1.0x10^9/l) and proceed with chemotherapy without any dose reductions for subsequent cycles with GCSF support on days 7-11 post chemotherapy.

7 CONCOMITANT MEDICATIONS

- All treatments being taken by the patient on entry to the study or at any time during the interventional phase of the study in addition to the study treatments are regarded as concomitant treatments and must be documented on the CONCOMITANT MEDICATIONS CRF.

- Throughout the study, investigators may prescribe any concomitant medications or treatments deemed necessary to provide adequate supportive care EXCEPT FOR THOSE LISTED IN THE SECTION BELOW.

Permitted, non-permitted medications and medications to be used with caution

- Investigators should refer to the SmPC for each of the drugs used for details relating to contraindications, special warnings and precautions for use, and interactions with other medical products and other forms of interactions. Up to date versions of these are available at http://emc.medicines.org.uk/
- **Live vaccines** (including inhaled flu vaccines) should not be administered to immunocompromised patients, including those receiving chemotherapy or within 6 months after receiving chemotherapy.

8 DOSE BANDING

Dose banding of all IMPs should be as per local practice. The following dose banding tables are for guidance only.

**Capecitabine (Xeloda®) Capsules (150mg, 500mg) – Daily Dose to be Divided**			
**Regimen**	**BSA (m^2)**	**AM Dose (mg)**	**PM Dose (mg)**
**Capecitabine 2000mg/m^2 Per day in 2 divided doses**	1.3	1000	1500
	1.4 – 1.6	1500	1500
	1.7 – 1.9	1500	2000
	2.0 – 2.3	2150	2150
**Capecitabine 1700mg/m^2 Per day in 2 divided doses**	1.3	1000	1150
	1.4 – 1.6	1000	1500
	1.7 – 1.9	1500	1500
	2.0 – 2.2	1500	2000
	2.3	2150	2150
**Capecitabine 1300mg/m^2 Per day in 2 divided doses**	1.3 – 1.5	800	1000
	1.6 – 1.7	1000	1150
	1.8 – 2.1	1000	1500
	2.2 – 2.3	1500	1500
**Capecitabine 1000mg/m^2 Per day in 2 divided doses**	1.3	650	650
	1.4 – 1.6	500	1000
	1.7 – 1.9	800	1000
	2.0 – 2.3	1000	1150

Fluorouracil (25 and 50mg/mL, 2.5g vials)											
**Dose SA**	**1.3**	**1.4**	**1.5**	**1.6**	**1.7**	**1.8**	**1.9**	**2.0**	**2.1**	**2.2**	**2.3**
**200 mg/m²**	250	275	300	325	350	350	375	400	425	450	450
**300 mg/m²**	400	425	450	475	500	550	550	600	650	650	700
**400 mg/m²**	500	550	600	650	700	700	750	800	850	900	900
**600 mg/m²**	800	850	900	950	1000	1100	1100	1200	1300	1300	1400
**900 mg/m²**	1200	1300	1400	1400	1500	1650	1650	1800	1950	1950	2100
**1,200 mg/m²**	1500	1650	1800	1950	2100	2100	2300	2500	2500	2750	2750

Folinic Acid (10mg/mL, 50mg and 300mg vials)											
**Dose SA**	1.3	1.4	1.5	1.6	1.7	1.8	1.9	2.0	2.1	2.2	2.3
**400 mg/m²**	500	550	600	660	660	720	790	790	870	870	950

Oxaliplatin (5mg/mL, 10mg, 50mg, 100mg vials)								
**Dose SA**	1.3	1.4	1.5	1.6	1.7	1.8	1.9	2.0
**65 mg/m²**	85	90	100	100	110	120	120	130
**85 mg/m²**	110	120	130	140	140	150	160	170
**100 mg/m²**	130	140	150	160	170	180	190	200
**130 mg/m²**	170	180	200	200	220	240	240	260

NB: CAPOX capped at 2.0

APPENDIX 3: RADIOTHERAPY

Although CRT is not under evaluation during the registration period of the trial this appendix describes the expected protocols for long course radiotherapy and relates to inclusion criterion 4. Information relating to CRT received should be recorded on the CHEMORADIOTHERAPY CRF at randomisation.

1 PRE-OPERATIVE CHEMO/RADIOTHERAPY

PATIENT SET UP

Patients are CT simulated prone or supine, with an anal marker and a comfortably full bladder. Preferably scanning will occur with intravenous and oral small bowel contrast.

1.1 TARGET VOLUMES

The disease is treated in two phases 45 Gy in 25 fractions to phase I and either 9Gy in 5 fractions or 5.4y in 3 fractions to phase II. Out-line phase II first to ensure that phase I is fully included in the phase II volume.

1.1.1 DEFINITION OF THE PLANNING TARGET VOLUME FOR PHASE II

The GTV is determined using diagnostic CT and MRI and clinical findings. Two GTVs may be drawn if there are involved lymph nodes (GTVtumour = GTV-T and GTVlymph nodes = GTV-N), which should be combined to give GTVcombined =GTV.

A 1cm isotropic margin is applied to GTV combined (GTV) to create CTVA

PTV phase II = PTV-2 is created by applying a 1cm isotropic margin to CTVA i.e. CTVA + 1cm

1.1.2 DEFINITION OF THE PLANNING TARGET VOLUME: PHASE I

CT planned3-D conformal/VMAT/IMRT volumes

CTVB: Clinical target volume for tumour, peri-rectal, presacral and internal and external iliac nodes (external iliac nodes only included if involved on MRI)

Lower Pelvis (CTVB): The caudal extent of this target volume should be a minimum of 2 cm caudal to gross disease(GTV), including coverage of the entire mesorectum to the pelvic floor. To identify the most caudal slice, the coronal images from the MRI or simulation should be used as a guide.

Mid pelvis (CTVB): The posterior and lateral margins of the target volume should extend to lateral sacrum and pelvic sidewall musculature (obturator internus). CTVB should reach the bone of the pelvic side wall where muscle is absent or were involved lymph nodes are in close proximity. The levator muscles help to define the mesorectum between bony pelvis and sacrum. Anteriorly: at least 1 cm anterior to the mesorectum (based upon information from simulation and MRI findings) Where margins are involved CTVB will need to expand to include CTVA (GTV + 1cm). Also in the mid pelvis, include at least the posterior portion of the internal obturator vessels (which lie between the external and internal iliacs in the mid pelvis). .

Upper pelvis (CTVB): The superior extent of CTV is at whichever is more cephalad: the S1-2 interspace or 2 cm cephalad to the superior extent of macroscopic disease in the rectum or clinically involved nodes (GTV). Internal iliac vessels (vein and artery are outlined plus a 7mm margin adapted to muscle and bone (unless pathological nodes are present). CTVB will include all of CTVA plus the adapted 7mm iteranl iliac vessels margin plus the pre-sacral area (minimum of 1cm anterior to sacrum). On axial slices, where internal iliacs are non-discernable, the anterior portion of the mesorectum is absent/non-definable and CTVA does not define CTVB, the anterior margin of CTVB will be represented by a smooth transition between The cephalad and caudal slices where one or more of these structures is identifiable. The external iliac vessels are only included if nodes are involved on MRI.

A 1cm isotropic margin is applied to CTVB (nominally CTVB+1cm).

PTV phase I (PTV-1) is created by combining the CTVB+1cm volume with the CTVA volume (this ensures that the phase I PTV (PTV-1) includes the entire phase II PTV (PTV-2)).

1.1.3 CTV INGUINAL NODES (FOR VERY LOW TUMOURS INVOLVING ANUS)

Where the treating clinician wises, the caudal extent of the inguinal region (CTV2) should be 2 cm caudal to the saphenous/femoral junction. The transition between inguinal and external iliac regions is somewhat arbitrary, but the level of the bottom of the internal obturator vessels (approximate bony landmark: upper edge of the superior pubic rami) as described above can be used.

All non target large and small bowel in the PTV and for 1cm above the PTV should be contoured and DVH recorded.

1.2 DOSE

Phase I: 45Gy in 25 fractions in 5 weeks.

Phase II: 9Gy in 5 fractions in one week (consider 5.4Gy in 3 fractions if significant amounts of small intestine within the 95% isodose).

For VMAT/IMRT plans; simultaneous integrated boost will be considered in discussion with the trials team.

Gaps in patient's treatment should be avoided and when occur for clinical or nonclinical (breakdowns) conditions, the consultant clinical oncologist should be advised and local protocol followed.

APPENDIX 4: SURGERY AND SURGICAL MORBIDITY

SURGERY

Surgery will be performed using a standardised technique for high-quality mesorectal dissection as described by Heald et al.[32] An extra-levator APE (ELAPE) is recommended for tumours extending beyond the intersphincteric plane; whereby a wider resection margin at the level of puborectalis can be achieved by an extended excision that includes a cuff of levator muscle.[33-36] In selected cases exenterative surgery will be required to achieve a clear circumferential resection margin. For the purpose of the trial, we recommend that this is performed in specialist centres.

Surgery will not be performed when:

- Post chemoradiation imaging (CT and MRI) of the pelvis demonstrates inoperable tumour or emergence of metastatic disease.
- The patient is eligible for deferral of surgery (there is a complete response to treatment).

SURGICAL MORBIDITY

All of the patients in this trial will have received radiotherapy and chemotherapy prior to surgery, which is believed to increase surgical morbidity. This is of greatest importance for those patients initially managed by deferral of surgery who later require surgery due to tumour regrowth.

Intra-operative and post-operative data will be collected. Post-operative complications will be recorded by clavien-dindo classification (table below). An anastomotic leak is defined as a clinically suspected leak, confirmed radiologically or at re-operation. Severity of anastomotic leakage should be graded according to the impact on clinical management:[37] Grade A anastomotic leakage results in no change in patients management, whereas grade B leakage requires active therapeutic intervention but is manageable without re-laparotomy. Grade C anastomotic leakage requires re-laparotomy.[37]

**Surgical Morbidity: Clavien-Dindo Classification**[38]	
**Grade 1**	Any deviation from the normal postoperative course without the need for pharmacological treatment or surgical, endoscopic and radiological interventions. Acceptable therapeutic regimens are: drugs as antiemetics, antipyretics, analgesics, diuretics and electrolytes and physiotherapy.
**Grade 2**	Requiring pharmacological treatment with drugs other than such allowed for grade I complications. Blood transfusions and total parenteral nutrition are also included.
**Grade 3**	Requiring surgical, endoscopic or radiological intervention.<br>3a - not under general anesthesia<br>3b - intervention under general anesthesia
**Grade 4**	Life-threatening complication (including CNS complications) requiring IC/ICU-management<br>4a - single organ dysfunction (including dialysis)<br>4b - multi organ dysfunction
**Grade 5**	Death. A serious adverse event (SAE) form must be completed.

APPENDIX 5: HISTOPATHOLOGY

HISTOPATHOLOGICAL EVALUATION OF THE RESECTION SPECIMEN

All pathology specimens will be prepared for evaluation in a standardized way, previously reported for the MERCURY and CR07 trials. Photographs of the specimens will be collected for central review of the quality of surgery and of the histology – to ensure these photographs are taken it is helpful to pre-warn the pathology department about the date of surgery and clearly mark the request form as being a TRIGGER trial case.

A full set of H and E stained glass slides and specimen photographs will be centrally reviewed and digitised at the Department of Pathology & Tumour Biology, Leeds Institute of Cancer and Pathology, University of Leeds, UK, the digitized pathology sections will then be archived on the BRC imaging and genomic profiling corelab library server. Central review at Leeds will ensure quality control – quality of surgery and analysis of potential response biomarkers such as ki67, tumour cell density. Centres will first send these slides to the Royal Marsden as detailed in the Laboratory Manual and can either send the original H&E stained glass slides or a set of copies.

All pathology data is routinely collected as part of the Royal College of Pathologists Reporting proforma, with the exception of effect of chemotherapy and radiotherapy on the tumour. This assessment of tumour regression is an important secondary outcome for The TRIGGER trial and mrTRG will be compared with Pathology assessment of tumour regression. Therefore the modified Mandard scoring assessment for chemo-radiotherapy response will be used.

Introduction

The guidance below is provided to assist the key role of the histopathologist in the assessment of the excised rectal cancer specimen. This section should be used in conjunction with the TRIGGER PATHOLOGY CRF.

Histopathologists at participating sites will be requested to collaborate in this trial in the following ways:

- By following the guidance in this appendix
- By submitting and/or facilitating submission information and specimens for central review
- By submitting and/or facilitating submission of archival tissue blocks for future tissue-based cancer research

Pathology dissection

High quality histopathology is a key component of this trial. The pathologist has a key role to play in assessing the circumferential resection margin (CRM), identifying and describing perforations and evaluating the planes of surgery of the mesorectum and the levator/anal sphincter. It is important that the pathologist determines the degree of response to therapy (tumour regression), retrieves substantial numbers of lymph nodes (in cases with strong chemotherapy and radiotherapy), and confidently identifies extramural venous invasion (EMVI) and peritoneal involvement to the highest standards. For the TNM staging in this trial, we are using TNM5, not TNM6 or TNM7. This is in line with current RCPath reporting recommendations. This is because of the poor reproducibility associated with the TNM6 definitions of EMVI and lymph nodes, and the introduction of tumour deposits (pN1c) in TNM7, which was based on little evidence with no assessment of interobserver variability. Thus in this trial, any tumour deposits that are 3mm or larger in size are classed as fully replaced lymph nodes (the so called '3mm rule'). Any deposit less than 3mm in size is counted as discontinuous tumour spread. This allows this trial to be consistent with other trials such as the Dutch TME trial and MRC CR07 trials. In order to collect further prospective data on the importance of tumour satellite nodules, we will ask you to separately list within the PATHOLOGY CRF the number of true lymph nodes identified, the number of true lymph nodes involved by tumour, the number of tumour deposits that are 3mm or greater in size (and therefore would be counted in the final lymph node count in TNM5) and whether or not additional tumour deposits less than 3mm in size are present.

Thank you for your efforts and for participating. The key issues are specimen photography, consistent high quality specimen dissection and providing the slides for scanning to create a permanent record of the pathology.

Preparation of the specimen

The surgeon will be asked to provide information regarding the height of the tumour and its location within the bowel wall on the histopathology request form. This will help the pathologist to identify the tumour and is particularly important in cases showing an excellent response to pre-operative therapy.

The intact specimen should be photographed whole prior to opening the bowel and further dissection. It is preferable for the specimen to be submitted fresh from the operating room to the pathology department as soon as possible after resection. Upon receipt in the pathology department, digital colour photographs should be taken of the anterior and posterior surface of the whole specimen, preferably prior to formalin fixation (see example below). It may be advisable to ask the surgeon to take photographs of the fresh specimen following resection if this opportunity is likely to be missed in the pathology laboratory. All photographs **must** include a metric ruler (for calibration) and the site of the tumour and the high vascular

ties should be marked (e.g. with forceps or pre-printed labels). If the tumour location cannot be ascertained by palpation, this should be indicated on the photograph with an additional label. Additional images of the lateral views (left and right side), close ups of the anterior and posterior surfaces of the levator/sphincters (in abdominoperineal excision specimens), close ups of any perforation site or other defects, and any other unusual findings should also be taken.

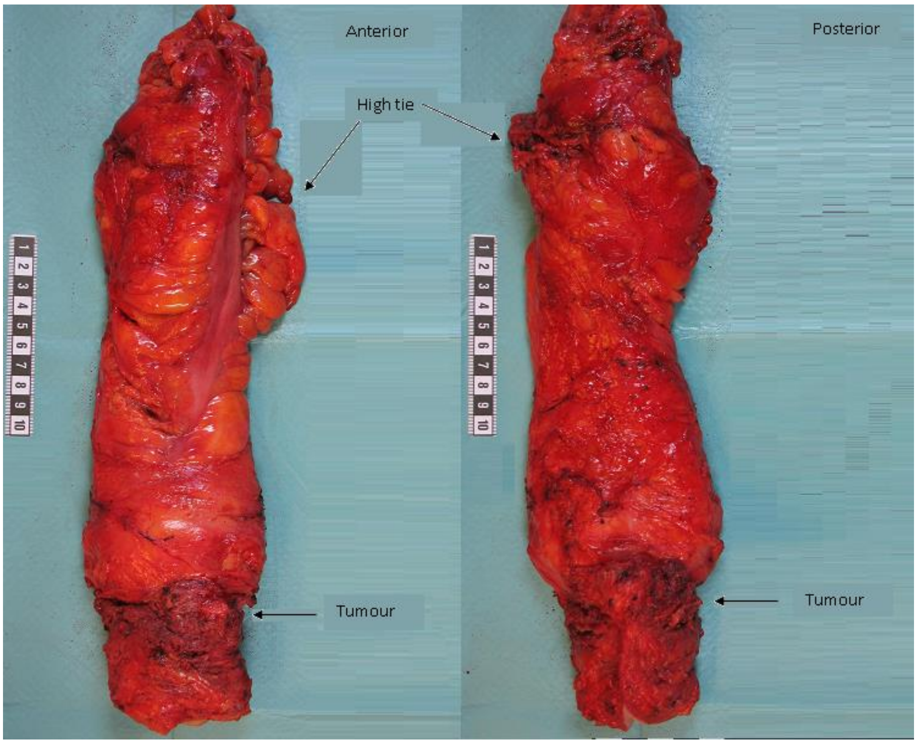

The plane of surgery should be assessed by the local pathologist on the intact specimen (prior to opening) for both the mesorectum and the levator/sphincters (as appropriate). This is preferably done on the fresh specimen prior to fixation. If the specimen is received already fixed in formalin, grading and photography can be done at this stage prior to opening the specimen. Surgeons should be asked not to open specimens prior to receipt in the pathology department as this can affect the assessment of the surgical planes and the status of the CRM. The grading systems for the planes of surgery are given below.

After photography and grading of the surgical planes, the specimen can be opened along the anterior peritonealised surface from the proximal margin down to a point approximately 20 to 50mm above level of the tumour or down to the level of the anterior peritoneal reflection if the tumour is lower down in the rectum. The mesorectum and bowel wall distal to the tumour should ideally be kept intact, although the distal resection margin can be opened if desired to aid fixation or obtain fresh tissue for local tissue banking. THE AREA OF THE TUMOUR MUST NEVER BE OPENED AS THIS

DESTROYS THE ANTERIOR CRM AND/OR PERITONEUM. A piece of foam/paper soaked in formalin can be inserted through the tumour if felt appropriate to aid fixation. The specimen should then be pinned out onto a cork board and placed in formalin fixative for approximately 48 hours prior to further dissection. It is acceptable to inflate the specimen with formalin after receipt, leave it to fix and then take photographs, but this should always be done prior to opening the specimen and undertaking any dissection.

Assessment of the plane of surgery

The planes of surgery in the area of the mesorectum and the levator/sphincters should both be graded separately (as appropriate). Thus for anterior resection specimens there will only be one grade - the grade for the mesorectum. For abdominoperineal excision specimens there will be a grade for the mesorectum and a separate grade for the levator/sphincters. The final specimen grade(s) should always be based on the area of the 'worst' plane of excision.

Quality of resection of the mesorectum (all specimens)

The quality of the mesorectal dissection can be determined from the intact specimen and confirmed from the cross-sectional slices. The three point grading system described below has been used in the MRC CR07 trial , MRC CLASICC trial and the Dutch TME/RT study, where poorer planes have been shown to predict a higher risk of local recurrence and poorer survival.

Plane	Description
**Mesorectal (good plane of surgery)**	The mesorectum should be smooth with no violation of the fascial covering. There should be a good bulk to the mesorectum both anteriorly and posteriorly, and the distal margin should appear adequate with no coning near the tumour. Any defect should not be more than 5mm deep.

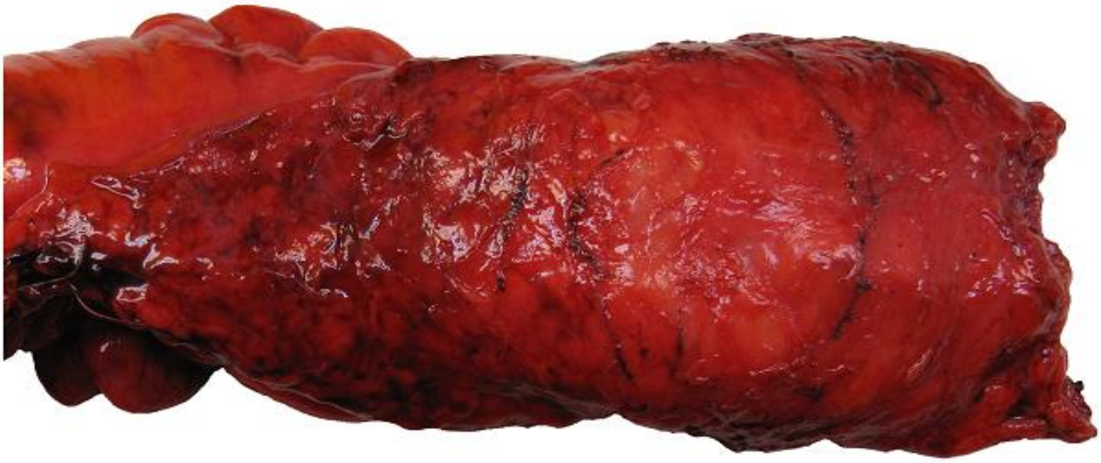

Mesorectal plane showing shiny fascial covering over the CRM and no defects

Plane	Description
**Intramesorectal (moderate plane of surgery)**	There should be a moderate bulk to the mesorectum with minor irregularity of the mesorectal surface. A moderate degree of coning of the specimen may be seen towards the distal margin. Importantly, the muscularis propria should not be visible, except at the area of insertion of levator muscles at the very distal aspect. There will be moderate irregularity of the CRM.

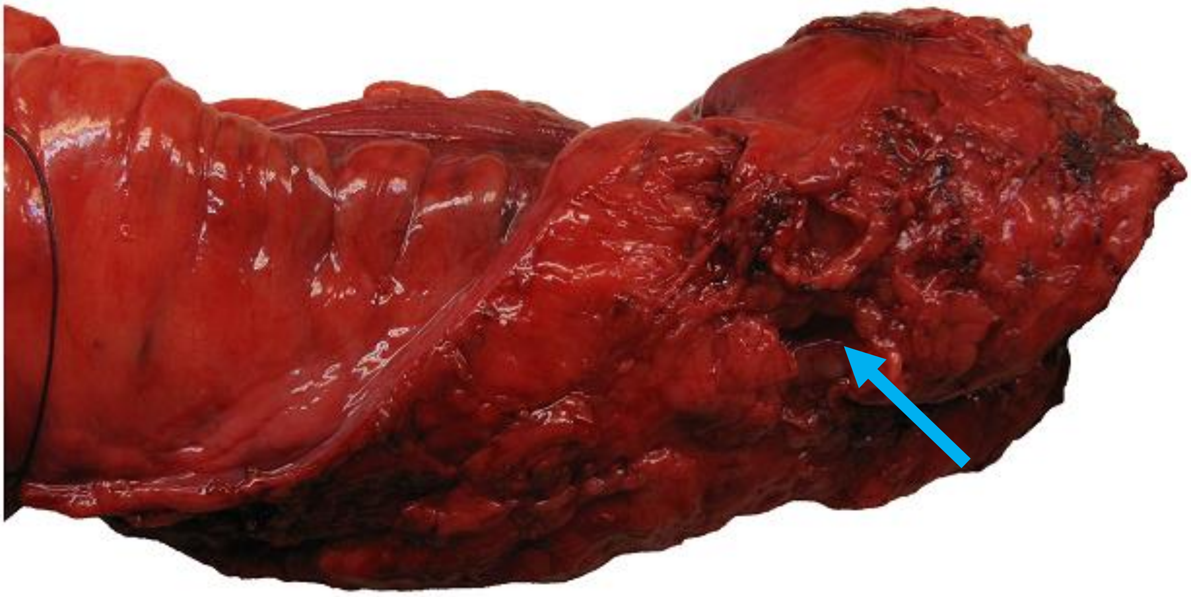

Intramesorectal plane with significant defects into the mesorectum without the muscularis propria being visible (blue arrow)

Plane	Description
**Muscularis propria**	There will be substantial areas where mesorectal tissue is missing with deep cuts and tears down onto the muscularis propria. On cross-sectional slicing, the CRM will be very irregular and formed by the muscularis propria in places.

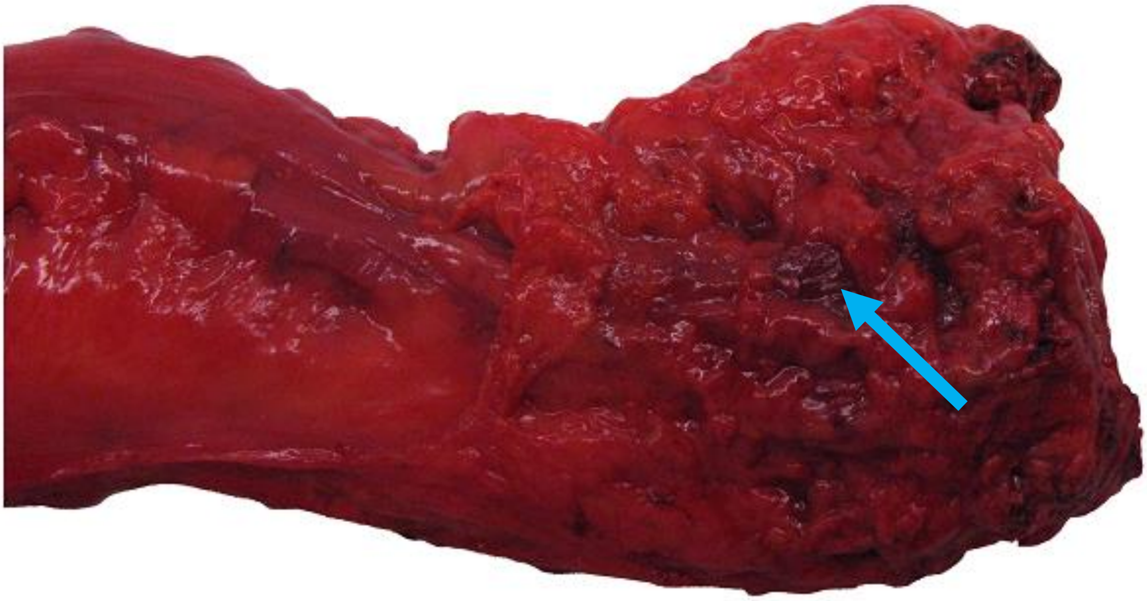

Muscularis propria plane with significant mesorectal defects exposing extensive areas of muscularis propria (blue arrow)

Quality of resection of the levators/sphincters (APE specimens only)

The quality of surgical dissection in the levator/sphincter area around the anal canal and below the mesorectum needs to be assessed separately in APE specimens.

PLANE	Description
**Levator**	The surgical plane lies external to the levator ani muscle, which are removed *en bloc* with the mesorectum and anal canal. This creates a more cylindrical-shaped specimen with the levators forming an extra protective layer above the sphincters. There should be no significant defects into the sphincter muscles or levators.

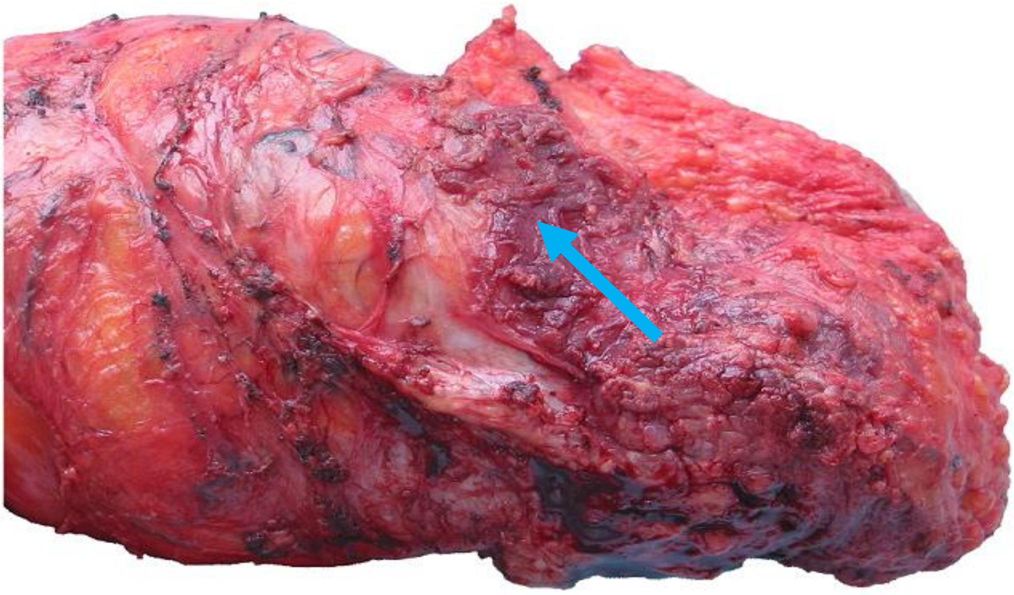

Levator plane showing levator muscles attached to the mesorectum (blue arrow)

**Sphincteric**	Either there are no levator muscles attached to the specimen or only a very small cuff, and the CRM is formed by the surface of the sphincter muscles. There should be no deviations into the sphincter muscle themselves. The specimen shows coning at the level of the puborectalis muscle resulting in the classical surgical waist.

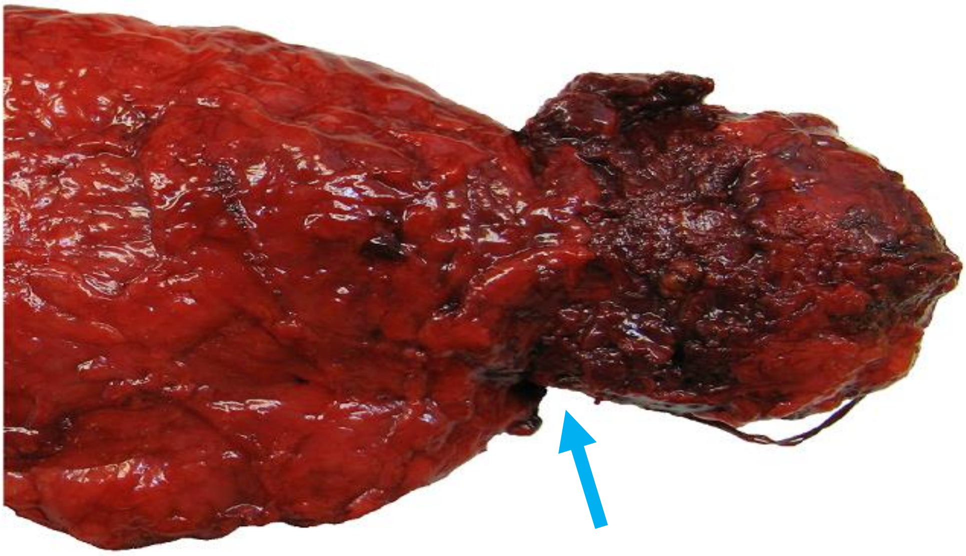

Sphincteric plane showing the classic surgical waist (blue arrow) with no levator wrap. A small amount of levator muscle is seen hanging loose on the opposite side to the arrow but this is not adherent to the mesorectum as would be seen in a levator plane excision.

**Intra-sphincteric / submucosal/Perforation**	The surgeon has inadvertently entered the sphincter muscle or even deeper into the submucosa. Perforations of the specimen at any point below the peritoneal refection should also be classified into this group.

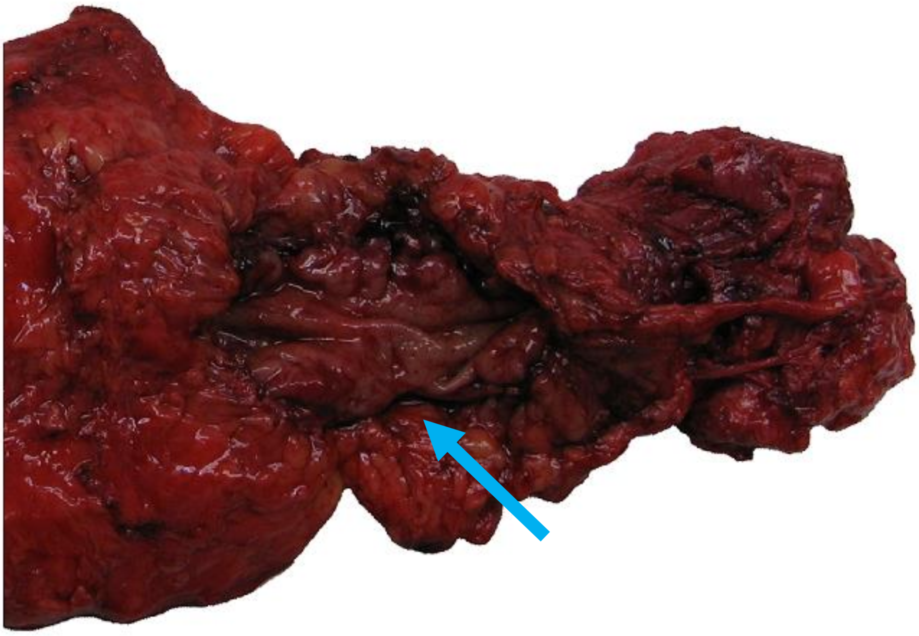

Intrasphincteric/submucosal/perforation plane showing a large anterior perforation (blue arrow) and a very irregular CRM with multiple defects into the sphincter muscles

Macroscopic specimen dissection

Once the whole specimen photographs have been taken and the planes of surgery graded (preferably on the fresh specimen but if not on the unopened formalin-fixed specimen), the specimen is ready to be dissected.

Assessing for the presence of intra-operative perforations

The specimen should firstly be described in detail, in particular, the pathologist should search for the presence of intra-operative perforations irrespective of whether these are located at the tumour site (tumour perforation) or in the rest of the bowel away from the tumour (bowel perforation). For perforations that involve the tumour site, it should be stated whether the perforation is in an area covered by peritoneum (TNM stage pT4) or in an area of a surgically created margin e.g. below the peritoneal reflection (RCPath guidelines also recommend staging these cases as pT4 and you should look very carefully for the presence of CRM involvement in the area of perforation). Additionally it is useful to document whether the perforation is above or at the height of the sphincters in APE specimens.

Relationship of the tumour to the anterior peritoneal reflection

The crucial landmark for recording the height of rectal cancers is the anterior peritoneal reflection. This is identified from the exterior surface of the anterior aspect of the specimen. Rectal cancers are classified according to whether they are (see diagram below):

1. Entirely ABOVE the level of the anterior peritoneal reflection
2. Astride (or AT) the level of the anterior peritoneal reflection
3. Entirely BELOW the level of the anterior peritoneal reflection

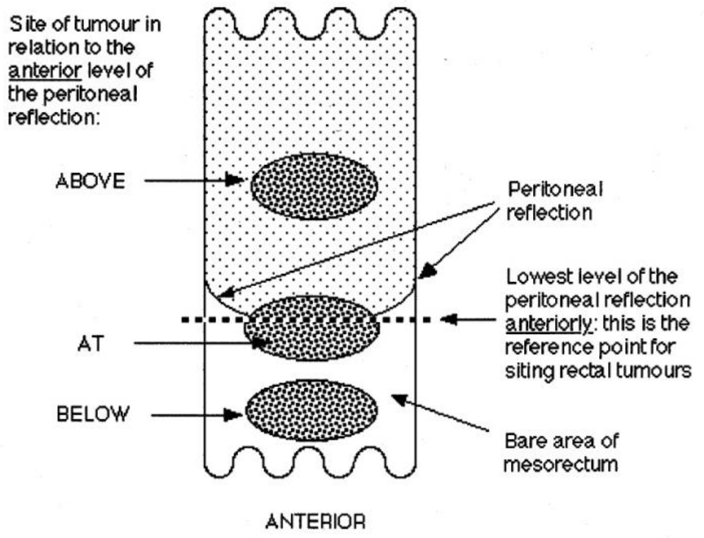

Distance from the tumour to the distal and proximal resection margins

This is measured from the longitudinal cut-ends of the specimen (distal and proximal). It is only necessary to examine the longitudinal margins histologically if tumour extends macroscopically to within 30 mm of one of these. For tumours located further away, it can be assumed that the cut ends are not involved. Exceptions to this recommendation are adenocarcinomas that are found on subsequent histology to have an exceptionally infiltrative growth pattern, show extensive vascular or lymphatic permeation or are undifferentiated carcinomas.

Inking the specimen

It is recommended that the whole of the CRM (i.e. the non-peritonealised mesorectum and levator/sphincters) is painted with ink (e.g. India ink or silver nitrate) before dissecting the specimen to facilitate the assessment of the CRM. Inking can be done before or after fixation according to local practice. It should be remembered that the CRM only applies to the surgically incised tissue planes and not the peritonealised surfaces. Anteriorly, the upper rectum is covered by peritoneum. Only the area below the peritoneal reflection is at risk of CRM involvement. The mesorectal surface of the CRM is larger posteriorly and extends up to a higher level than it does anteriorly (see diagram below).

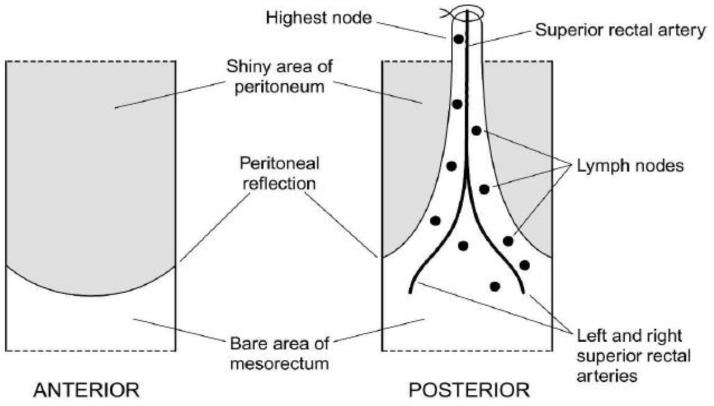

Cross-sectional slicing and photography

The specimen should then be cross-sectioned into slices as thinly as possible (3 to 4 mm thickness is recommended) starting from the distal resection margin to at least the anterior peritoneal reflection or 20 to 50 mm above the tumour if this is higher. These slices should be laid out in order on a board and the cut surface presented should be consistent in all of the slices (preferably the distal aspect to correlate with the MRI scans). The slices should then be photographed, either as a whole (with additional close ups of individual slices containing the tumour), or alternatively individual photographs of each slice can be taken. It is important that it is made clear on the photographs which slice is the most distal and the most proximal

one by using appropriate labels. All photographs **must** always include a metric ruler scale for calibration. An example of cross-sectional slicing and photography is given below.

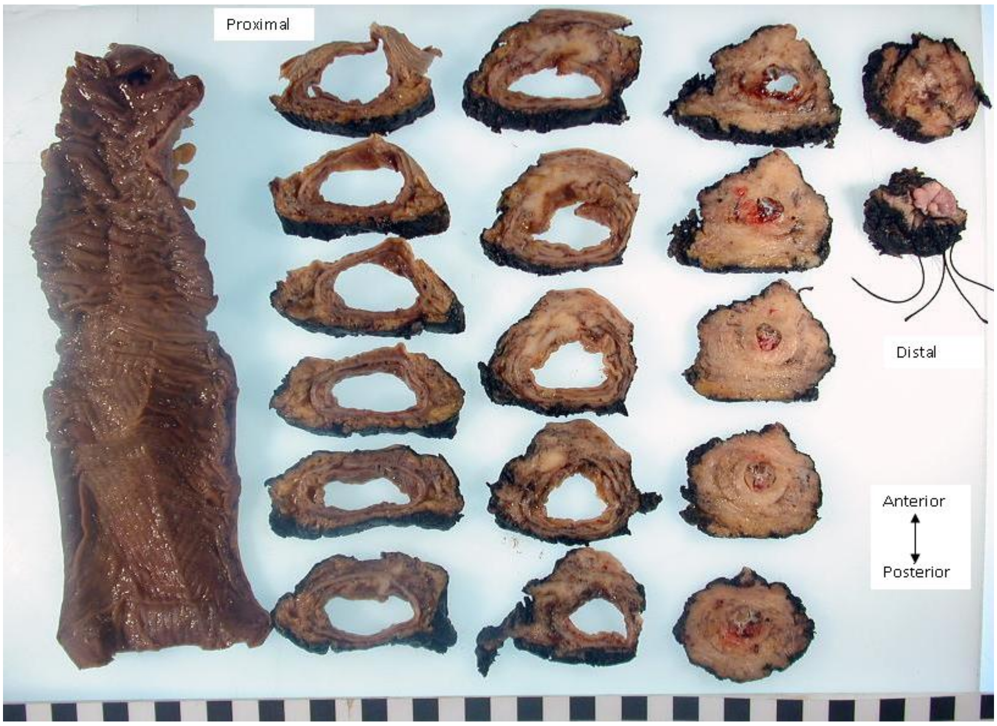

Assessment of the CRM and maximal extent of tumour spread

The cross-sectional slices should be carefully assessed after photography and the minimum distance of the macroscopic tumour to the inked CRM as well as the maximum depth of tumour invasion beyond the outer muscle coat of the muscularis propria should be recorded. In APE specimens, both the maximal extent of tumour spread beyond the muscularis propria at the level of the mesorectum and additionally the maximal extent of tumour spread beyond the internal sphincters at the level of the levator/sphincters should be recorded.

These macroscopic measurements should be confirmed histologically, preferably on whole mount sections e.g. using the Vernier scale. The minimum distance from the tumour to the CRM should be reported to the nearest millimetre apart from CRM positive tumours (1 mm or less from the inked CRM), which should be reported to the nearest 0.1 mm. If the position of the muscularis propria and/or internal sphincter is obscured by tumour or fibrosis, the position of these structures should be estimated by comparison to subsequent slices.

Position of the tumour

The position of the tumour should be noted on the TRIGGER PATHOLOGY CRF. This involves documentation of the quadrant of involvement from the cross-sectional slices – i.e. anterior quadrant, posterior quadrant, lateral quadrant or combinations of these. Also it would be helpful to trace the position of the tumour at the point of maximum extension both above the sphincters in the region of the mesorectum and below the levator/sphincters on the diagrams provided in the CRF (see below for a copy of the CRF diagram).

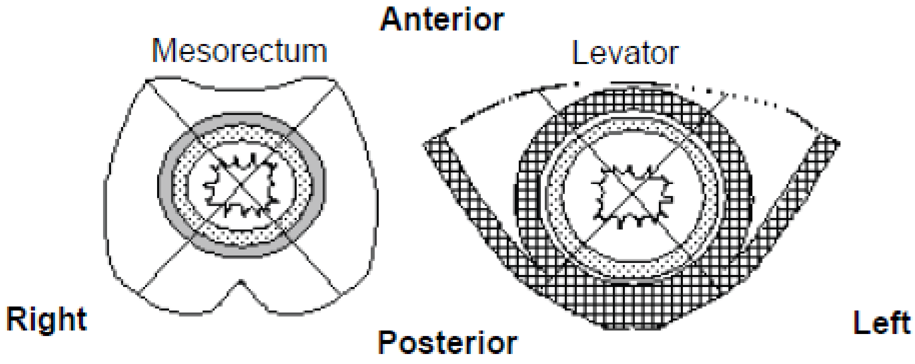

To correlate the position of the tumour with the MRI report, the location of the tumour should be described using a clock-face with the anterior peritoneal reflection being 12 o'clock and looking at the specimen slice from the distal aspect

Sampling the specimen

If possible, each tumour bearing slice should be processed into a 'large' (mega) block to produce whole mount sections. However, it is recognised that this is not possible in all laboratories, and therefore a minimum of 5 tumour blocks should be taken (either in standard or large cassettes or a combination of both). Tumour blocks should be taken to demonstrate the point of deepest tumour invasion, areas suspicious for CRM and/or peritoneal involvement, and areas with possible extramural vascular invasion. Please see below for the sampling protocol in cases where tumour cells are difficult to find after pre-operative therapy.

All of the lymph nodes within the specimen should be identified, retrieved and assessed, regardless of their site and size. A running mean of at least fifteen is to be expected. The number of positive lymph nodes must be equal to or less than the number of lymph nodes sampled. The apical node (the lymph node closest to the high vascular tie) should be identified and embedded separately to allow staging according to Dukes' classification. If lymph nodes lie close to or against the CRM then these should be blocked out in such a way that the minimum distance from any tumour to the CRM can be assessed.

Microscopic reporting

Peritoneal involvement and extramural vascular invasion

Involvement of the peritoneum by tumour should be carefully looked for and is defined as per the definition of Shepherd et al (see figure below). Tumour cells must actually perforate through the serosa and lie on the surface of the specimen. It is expected that on average peritoneal involvement will be present in 10% of rectal cancer specimens.

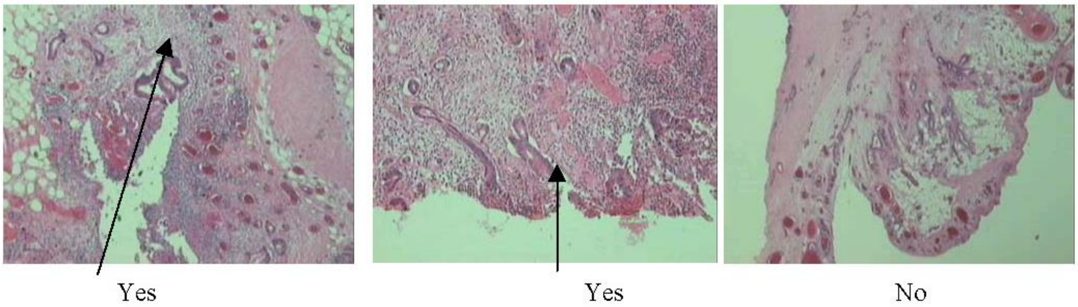

Peritoneal involvement with tumour penetrating the peritoneum

Extramural venous invasion is defined as involvement of a vascular structure which has smooth muscle in the wall and can frequently be seen macroscopically as finger-like protrusions extending beyond the muscularis propria. If tumour is present close to an artery and the accompanying vein is not visible, then there should be a high level of suspicion for vascular invasion. This should be looked for closely as it is often missed. On average, it is expected to see extramural venous invasion in greater than 25% of rectal cancer specimens.

CRM involvement

Involvement of the CRM by tumour is defined as viable tumour cells being present at or within 1 mm of the inked CRM. The CRM is at risk not only from direct tumour spread but also metastatic deposits in lymph nodes that lie close to or against the CRM, and through extension along lymphatics, blood vessels and nerves. If the CRM is involved by tumour then the mode of involvement should be stated (e.g. primary spread, lymph node deposit, isolated tumour deposit, vascular, lymphatic, perineural etc.), as well as the minimum distance between the closest tumour and the CRM. The maximum length of CRM involvement in millimetres should also be estimated in the slice showing the greatest extent of CRM involvement.

If the CRM is free of tumour it should be noted whether there is normal tissue at the margin or whether the margin contains abnormal fibrotic tissue suggestive of tumour regression.

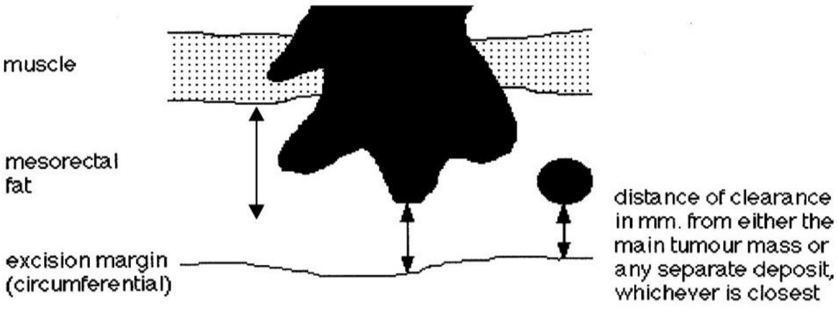

pT staging of low rectal cancers

The pT-staging of cancers above the sphincters is straightforward, however, many low rectal cancers are partly located within the region of the sphincters. The anatomy of the levator/sphincters area is very complex and shows considerable variation between individuals. pT staging of adenocarcinoma in the area of the sphincters is currently controversial. Both TNM6 and TNM7 state that such tumours should be staged as anal cancers according to tumour size. However, in the absence of a robust evidence-based staging system, the only solution is to separately describe the anatomical extent of tumour spread both above the sphincters (in the area of the mesorectum) and at the height of the levator/sphincters to allow subsequent analysis.

Tumour differentiation

The grade of differentiation of the tumour should be defined by the predominant area of tumour and not on the area of the worst grade. Other types of differentiation, i.e. mucinous adenocarcinomas, signet ring adenocarcinomas and undifferentiated tumours should be documented.

Lymph node assessment and tumour deposits

As stated in TNM5, extramural tumour deposits measuring ≥3 mm in maximum size are counted as involved lymph nodes even if no residual lymph node structure can be identified. Smaller deposits are regarded as apparent discontinuous extensions of the main tumour. The number of ≥3mm tumour deposits classified as lymph nodes should be indicated in the report separately to the number of true lymph nodes. Mucin or fibrosis only within a node should be commented upon in the report as a sign of tumour regression but should not be classed as lymph node metastases. In the TNM staging system, pN1 corresponds to involvement of 1-3 nodes and pN2 to involvement of 4 or more nodes.

Preoperative chemoradiotherapy regression scoring

The modified-Mandard method is recommended and is summarised below. **However, it is preferable to use descriptive text rather than a numeric grading system to avoid confusion with other tumour regression grading systems**. The final grading should be based on the overall assessment of all tumour blocks (there should be a minimum of five) but should not include the assessment of lymph nodes.

pTRG1 - **No residual cancer**

pTRG2 - **Rare/Scant residual tumour cells**

pTRG3 - **Fibrosis outgrowing residual cancer**

pTRG4 - **Residual cancer outgrowing fibrosis**

pTRG5 - **Absence of regressive changes**

Assessment of specimens where tumour cells are difficult to find

Where tumour cells cannot be found on the first assessment of at least five blocks of tumour, the whole area of the fibrotic scar should be embedded and examined. If no tumour cells can be seen following assessment of these extra blocks, then three deeper levels should be taken and examined from each fibrotic scar block. If after these assessments still no tumour cells are identified then the tumour should be considered to have undergone a complete pathological response (TNM stage ypT0 ypN0). Additional levels beyond the above should not be taken, as it is important to standardise the degree of effort made to find residual tumour cells.

Collection of photographs, tissue, and Case Report Form

Photographs

- Photographs of the resection specimen should be taken with a digital colour camera and include:
    - The whole intact resection specimen from both the anterior and posterior aspects. Additional lateral views and close ups of the sphincters (if present), any defects or perforations will be helpful. Photographs of the fresh unopened specimen are preferable although fixed specimens are acceptable. The site of the tumour (if palpable) and the closest high vascular tie should be marked (e.g. with pre-printed labels or forceps).
    - All of the serial cross-sectional slides in order (3 to 4 mm slices from the distal margin of excision to a level above the tumour). Please mark the proximal and distal slices with labels and indicate the direction if not clear. These can be either taken as one whole image (containing all slices) or sequential images of individual slices.
- All photographs **must** include a metric scale to allow calibration.
- The whole specimen (or slices) should be visible in the image. A white background is ideal although any plain colour is acceptable. Photographs should be taken directly above the specimen and not at an angle to reduce any distortion artefact.

- Images should **not** contain any direct patient identifiers (e.g. name, NHS number) but should be identifiable by trial ID (TRIGGER CCR4326), site code, patient's initials, and patient ID number.
- All images should be copied to CD-ROM and sent to the TRIGGER Trial Office:

  Prof Gina Brown, Department of Radiology, Royal Marsden Hospital, Downs Road, Sutton, Surrey, SM2 5PT
- If images cannot be copied to a CD-ROM, please contact Dr. Nick West (n.p.west@leeds.ac.uk) to discuss possible alternative arrangements.

Tissue and reports

All of the H&E stained glass slides from both the original diagnostic biopsy and the surgical resection specimens, along with the FFPE blocks should be sent to the Royal Marsden for central archiving (as detailed in the Laboratory Manual) along with a copy of the anonymised histopathology reports. Copies of the slides can be sent if the local site does not want to release the originals. Alternatively high resolution digital slides can be sent if slide scanning facilities are available locally. These will subsequently be sent to the University of Leeds as batches at the end of the feasibility trial for scanning .

Slides (originals or copies) and reports should be identifiable by trial ID (TRIGGER CCR4326), site code,patient's initials, and patient ID number. Any direct identifiers, e.g. patient name and NHS number, should be blanked out on the slides

TISSUE MICROARRAYS

At the end of the feasibility trial FFPE blocks (See Section 10.4) received, processed and archived at the Royal Marsden will be sent to the ICR/Royal Marsden BRC for DNA and RNA Extraction and then forwarded to the University of Southampton for the preparation of Tissue Microarrays (TMA), H and E plus MRI guided, to enable further work with molecular markers and genomic profiling . TMAs will be analysed at the University of Southampton, St James Hospital in Leeds (Prof P Quirke), RMH/ICR BRC (Irene Chong) and Radboud University (Prof I Nagtegaal). Paraffin embedded tissue blocks will returned to the trial sites after TMA samples have been taken

APPENDIX 6: MRI PROTOCOL

Radiologists at participating sites will be requested to collaborate in this trial in the following ways and will be asked to sign the Delegation Log confirming these responsibilities:

- By ensuring that 0.6 x 0.6 x 3mm high-resolution images (1mm 3voxel size) will be undertaken.
- By ensuring that MRI and local rectal cancer staging will be undertaken by standardised proforma based reporting
- By ensuring that assessing response to treatment will be undertaken by standardised proforma based reporting
- By submitting and/or facilitating submission (by the Trial Coordinator/delegated individual) of anonymised scans promptly to the Royal Marsden for central archiving

Prior to taking part in this study the lead GI Radiologist and nominated deputy will be invited to attend a workshop/webinar to standardise reporting and mrTRG assessment for the trial. Further support workshops/webinars will be held to assist with image interpretation and surveillance of patients undergoing deferral of surgery.

MAGNETIC RESONANCE IMAGING (MRI) - RECOMMENDED TECHNIQUE

Buscopan I.M improves image quality. Intravenous contrast enhancement with gadolinium is not recommended for the staging of rectal cancer. A 1.5-T (or where possible 3.0T) system is used with phased-array coils. It is important that the coil is optimally centred to ensure adequate coverage of the rectum, mesorectum, and anal sphincter complex. It is crucial to encompass the lymph node draining territory, which is 5cm above the tumour; the lower edge of the tumour must lie at least 10cm below the symphysis pubis and the upper limit of the sacral promontory.

Initial localization images in the coronal and sagittal planes are needed to plan further high-resolution images. A narrow field of view (160mm x 160mm, 256 x 256 matrix) with a minimum of 4 signal averages to ensure a 0.6 x 0.6 x 3 mm high-resolution image (1mm 3voxel size). The first series are T2-weighted sagittal, turbo spin-echo sequences from one pelvic sidewall to the other enable identification of the tumour. The second series consists of wide-field-of-view axial sections of the whole pelvis. The third series consists of the high-resolution images that are T2-weighted thin-section axial images through the rectal cancer and adjacent tissues. These sequences must be performed perpendicular to the long axis of the rectum and at the level of the tumour (3-mm slices); otherwise, the partial volume effects may cause a distorted image prone to over-staging.

For patients with low rectal cancer, the fourth series consists of high-spatial-resolution coronal imaging that will optimally show the levator muscles, the sphincter complex, the intersphincteric plane, and the relationship to the rectal wall. The post-treatment MRI follows exactly the same protocol.

For all tumours the high resolution scans should cover 5cm above the top of the tumour to capture upward spread to lymph nodes and vessels.

MRI & LOCAL RECTAL CANCER STAGING

T STAGING AT BASELINE

The TNM is a pathological staging system, the great benefit of MRI is that the pathological stage can be predicted, allowing an opportunity for neoadjuvant therapy. The T stage represents the depth of tumour invasion in relation to the bowel wall, this is a key component of the staging classification. There are four T stages: T1 – submucosal invasion; T2 – muscularis propria invasion; T3 – beyond muscularis propria into the subserosa, or into non-peritonealised pericolic or perirectal tissue; T4 – perforation of visceral peritoneum (T4a) or direct invasion of peritoneum (T4b). T3 and T4 tumours are associated with extramural invasion. The depth of extramural invasion is recognized as an independent prognostic factor. This is determined by the extent of tumour penetration into the fatty lymphovascular envelope of the mesorectum that surrounds the bowel wall.

Increased penetration into the mesorectum is associated with a worse prognosis and a higher rate of local recurrence. The majority of tumours are staged as pT3 and in terms of outcome there is a great deal of heterogeneity within the pathological T3 classification (Merkel et al, 2001). The T3 stage is now sub-classified to allow for this: (T3a minimal invasion: <1 mm beyond the border of the muscularis propria; pT3b-slight invasion: 1–5 mm beyond the border of the muscularis propria; T3c-moderate invasion: >5–15 mm beyond the border of the muscularis propria; T3d-extensive invasion: >15 mm beyond the border of the muscularis propria).

MRI can identify the layers of mucosa and muscle through distinct signal characteristics. T2-weighted images are particularly useful for this. The mucosal layer is seen as a very fine line of low signal intensity overlying the much thicker and higher signal of the submucosa. Outside this the muscularis propria can be seen as a dual-layer representing the inner circular and the outer longitudinal muscle layers. The latter has a typically irregular appearance due to vessels traversing the rectal wall. The perirectal fat is identified as a high signal surrounding the relatively low signal intensity of the muscularis. The fine layer of low signal intensity, representing the mesorectal fascia, envelops all of this. Determining the T stage of the tumour depends on careful assessment of the images with respect to the signal characteristics and correct field

alignment. The T stage is defined by the extent of signal intensity within and beyond the muscularis of the rectal wall.

CIRCUMFERENTIAL RESECTION MARGIN (CRM)

mrCRM is assessed by measuring the distance of tumour at its invasive border to the mesorectal fascia. Tumour is considered potentially involving CRM if the distance is less than 1mm.

EXTRAMURAL VASCULAR INVASION (EMVI)

The radiological characteristics of EMVI as seen on MRI have been described in detail and this is present in 30-40% of patients with rectal cancer. Veins around the rectum are recognised on T2-weighted images as serpiginous or tortuous linear structures, there is a signal void in the tubular structures thought to be vessels in addition to changes in contour. Ideal assessment of mrEMVI must include the following: pattern of tumour margin (extension into small veins may produce a nodular border); location of tumour relative to major vessels; vessel calibre (tumour causes vessel expansion and increase in tumour signal in the lumen); and vessel border. Vascular invasion can be contiguous with the tumour or discontinuous in vessels remote from the tumour within the mesorectum.

Sometimes numerous veins are evident at the advancing edge of the tumour but there is no expansion of these veins by tumour signal. Although this may correlate with microscopic vascular invasion on pathology this has not been shown to be of prognostic significance on follow up. We therefore record these as 'minimal vascular spread' but mrEMVI negative.

Distinction between small, medium and large vein invasion:

Large = named anatomical veins e.g. superior rectal vein, middle rectal vein, inferior rectal vein

Medium = branches of named anatomical veins

Small = usually perirectal veins which are not major branches of named anatomical veins (their prognostic significance is less important).

TUMOUR HEIGHT: THE PROBLEM WITH LOW RECTAL CANCERS

The height of rectal cancer is usually measured from the anal verge and can be based on clinical examination, endoscopy or radiological assessment. The rectum is arbitrarily divided into three parts. In the lower rectum the mesorectum tapers sharply, consequently the distance between the outer musclularis propria and the mesorectal fascia decreases this increases the likelihood of the tumour being in close proximity or breaching the distal TME plane. Furthermore the surgery is technically challenging, with the additional constraints of limited access in a narrow pelvis. There is a conflict between the optimal oncological approach and how best to preserve bowel function. Tumours that threaten or breach the intersphincteric plane are associated with a high risk of CRM involvement and in such cases an ELAPE would

be recommended. Table 1 below describes the 4 stages of the Low Rectal Cancer (mrLR) system. This system has recently been prospectively validated in the Mercury II: Low Rectal Cancer study.

TABLE 1.

	Stage	Description
A	Stage 1	Tumour confined to the bowel wall and does not extend through the full thickness (intact outer muscle coat)
A	Stage 2	Tumour replaces the muscle coat but does not extend into the intersphincteric space
B	Stage 3	Tumour invades the intersphincteric space or lies within 1mm of the levator muscle
B	Stage 4	Tumour invades the external anal sphincter and is within 1mm and beyond levators muscle with or without invading the adjacent structures.

A : TME plane is considered safe by MRI

B : TME plane is at risk of CRM involvement. ELAPE surgery is indicated

ASSESSING RESPONSE TO TREATMENT

MAGNETIC RESONANCE TUMOUR REGRESSION GRADE (mrTRG)

Following chemo-radiotherapy a number of radiation-induced tissue changes occur. These include oedema, inflammation, necrosis and fibrosis. The assessment of post-irradiated tissue is challenging with all imaging modalities. However, advances in MRI have enabled high-resolution (3 mm) slices to be oriented through the plane of the tumour, consequently the accuracy of MRI has increased. This improved appreciation of the reactive changes that occur in normal rectal tissue after chemoradiotherapy and led to a better understanding of the appearance of residual disease on the post-treatment MRI.

These improvements have allowed an MRI tumour regression grade (mrTRG) to be developed, which allows the tumour site to be assessed at the end of conventional neoadjuvant treatment (**Table 2**). This scoring system is based on the pathological tumour regression grade systems, and most closely resembles the Mandard pTRG:

mrTRG 1 – Complete radiological response (linear scar only)
mrTRG 2 – Good response (dense fibrosis, no obvious tumour signal)
mrTRG 3 – Moderate response (>50% fibrosis and visible intermediate signal)

mrTRG 4 – Slight response (mostly tumour)

mrTRG 5 – No response/re-growth of tumour

THE POST-TREATMENT NODE STAGE (ymrN stage)

The optimal technique for performing the staging MRI is described above but we emphasise that optimal node assessment requires a narrow field view (160mm x 160mm, 256 x 256 matrix).

The morphologic criteria (i.e., irregular edge, signal heterogeneity, and size) for determining lymph node status on MRI also apply after CRT. However changes in the morphologic appearance of lymph nodes, such as a high-signal from mucinous tumours, can also occur. MRI interpretation of lymph node status after CRT has been established as reliable with reports of accuracy as high as 87%. In the study by Yu et al. described above, as well as assessing ymrT stage, the node morphology criteria were used to assess the impact of post-treatment node (ymrN) status on DFS. The 3 year DFS for the 'responders' (ymrN0) and 'non-responders' (≥ymrN1) was 74% and 63% respectively (p=0.04).

Table 2

Grade	Pre-treatment	Post-treatment
mrTRG1	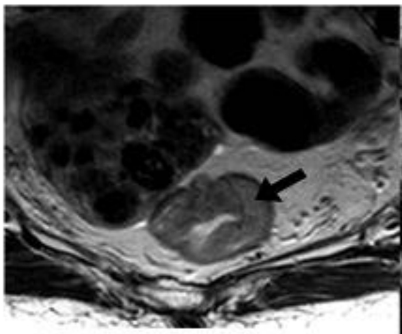	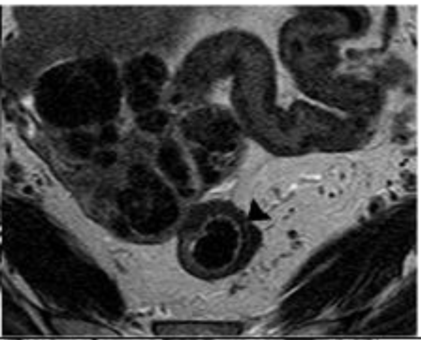
mrTRG2	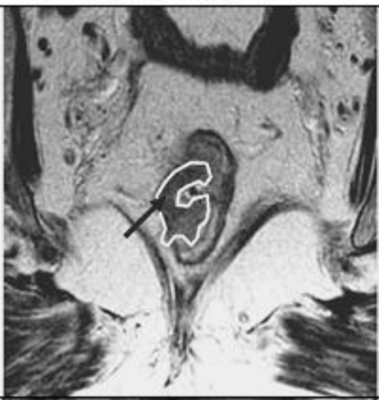	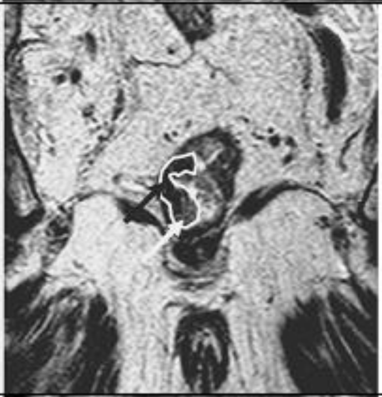
mrTRG3	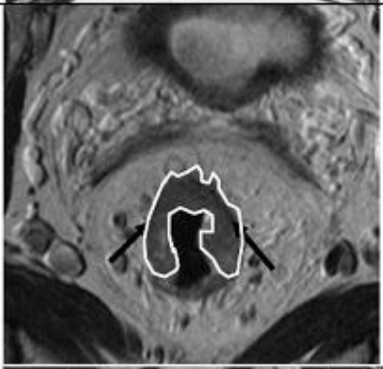	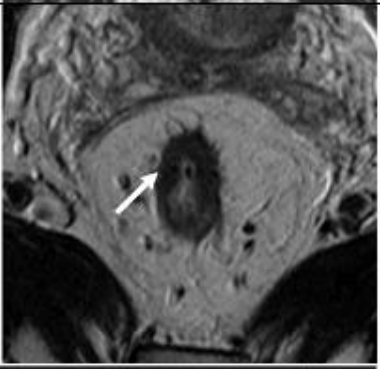
mrTRG4	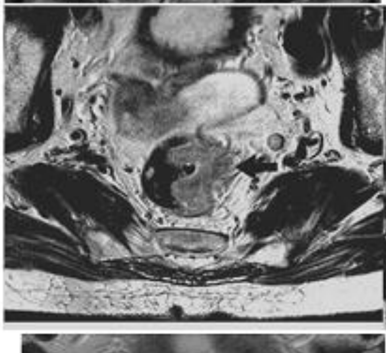	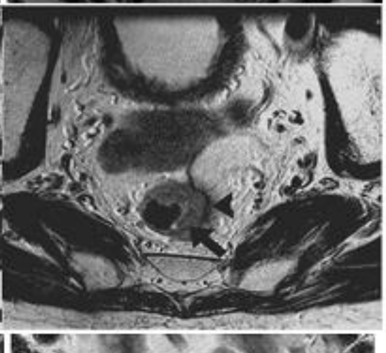
mrTRG5	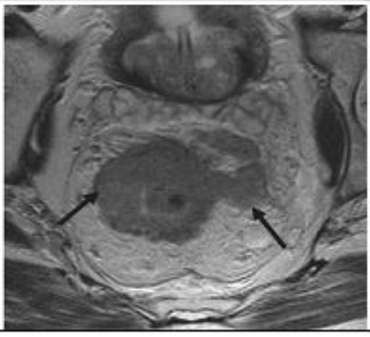	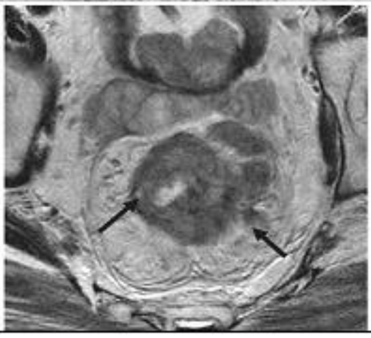

IMAGE

TRANSFER

Please refer to the image transfer section of the Imaging Manual for details relating to scan anonymisation and transfer to the TRIGGER Trial Office. These scans will be stored electronically and the central review team at the Royal Marsden will review the scans retrospectively in batches and copies of the analytic data will be kept in the TRIGGER Trial Office at the Royal Marsden. **It is important to note that the local MRI reports, including mrTRG reported for those in the intervention arm, will be used by the site study team to guide patient management, not the central review.**

APPENDIX 7: TRANSLATIONAL BIOMARKER SUBSTUDY

The TRIGGER protocol includes a translational substudy aiming to identify prognostic and predictive biomarkers in colorectal cancer. At the time of trial registration all patients are asked to consent to the transfer of their tumour tissue to the central laboratory and the collection of an additional research blood sample. At randomisation patients are asked to consent to samples of resected tissue collected at surgery to be sent to the central laboratory and the collection of further blood samples during the trial as detailed in Section 10.4 of the protocol.

Any patient material collected as part of this trial for research will be used with the only purpose to correlate tumour biomarkers with clinical characteristics and treatment outcome. All specimens will be anonymised prior to transfer to the Central Laboratory. Trial data associated with the Translational Sub-study will be stored on access-controlled IT systems at the Institute of Cancer Research.

1. RATIONALE

The molecular determinants of response to definitive rectal CRT are currently poorly understood. Candidate biomarkers from preliminary studies require further validation within large prospective clinical and radiological datasets to be deemed truly robust and fit for purpose for future patients [Sadanandam A, Nat Med 2013]. The TRIGGER trial represents a unique opportunity to undertake comprehensive 'genotype-phenotype' comparisons resulting in meaningful clinical benefit of future patients.

2. STUDY AIM AND OBJECTIVES

To assess whether CRC molecular sub-classification and markers of proliferation and apoptosis can predict for response to rectal CRT. This will ultimately help to identify patients who are most and least likely to benefit from neo-adjuvant CRT.

PRIMARY OBJECTIVE

- To assess whether the colorectal molecular sub-classification of tumour tissue according to consensus classification predicts for tumour response to rectal CRT as defined by pathological and MR TRG

SECONDARY OBJECTIVES

- To determine the impact of CRC molecular sub-classification of tumour tissue on disease free survival, overall survival and local recurrence
- To determine the effect of apoptosis and proliferation rates on response to CRT, disease free survival, overall survival and local recurrence
- To assess whether the detection of ctDNA predicts for relapse in patients with locally advanced rectal cancer

EXPLORATORY OBJECTIVES

- To evaluate whether the presence of a specific mutation or pattern of mutations identified in both FFPE tumour tissue and cfDNA predict for relapse
- To use targeted next generation sequencing (NGS) in plasma to identify mutations in ctDNA
- To assess the utility of massively parallel sequencing of rectal cancer stroma to predict for response to CRT
- To assess the feasibility of *ex-vivo* genomic and functional profiling using fresh tissue from the TRIGGER trial
- To assess whether candidate driver mutations and commonly disrupted signalling pathways, detected by massively parallel DNA sequencing, in predicting response to rectal CRT and overall outcome

3. STUDY DESIGN AND METHODOLOGY

This is a multi-centre, prospective, translational research study involving the collection and analysis of tumour and stromal tissue and serial blood samples in patients with locally advanced rectal cancer recruited to the TRIGGER trial. Blood and tumour samples will be collected at time-points specified in Section 10.4 of this protocol. The molecular analyses will be undertaken centrally at the Institute of Cancer Research/Royal Marsden BRC.

FFPE tumour tissue will be retrieved prior to CRT and at the time of surgery. The tumour will be assessed for tumour cellularity. DNA and RNA will be extracted using standard kits. RNA will be extracted in order to enable the tumour tissue to be molecularly sub-classified according to concensus classification [Sadanandam A, Nat Med 2013] and the assessment of other potential biomarkers such as micro-RNA. DNA will undergo quality control and undergo massively parallel sequencing along side germline DNA to identify somatic mutations.

We will design genotyping assays against the common hotspots for genes such as *KRAS*, *NRAS*, *TP53*, *PIK3CA*, *BRAF* and *APC* to identify tumour mutations in the cfDNA. For tumour specific mutations, digital droplet PCR (QX200, Bio-Rad) will be used and run in triplicate. Multiplexing of assays may be developed to reduce running costs.

FFPE blocks and H&E slides from pre-CRT biopsies and post-CRT resection specimens will be collected at the central laboratory at the Royal Marsden as described in Section 10.4. TMA analyses will be undertaken in collaboration with Southampton University, St James' Hospital, Leeds and the Institute of Molecular Sciences at Radboud University, Nijegan, Netherlands. All samples will be returned to the central Laboratory following analysis. TMAs will be constructed using a fixed pattern. Tissue from the specimen will be punched (4-6 cores per patient) from the invasive edge, the centre, the luminal surface and a normal

mucosa core. IHC for the antigen of each apoptotic and proliferation marker will be performed on all pre- and post-CRT blocks to determine apoptotic/proliferative indices.

Subject to funding, it is anticipated that surplus blood and tissue collected during this study may also be used for future research projects, where patients have provided consent. Access to these samples for future research will only be available following agreement amongst members of the trial management group. Additional research may involve (but is not limited to) epigenomics, proteomics, metabolomics, tumour biology and genomics (up to whole genome sequencing) as well as epidemiological studies.

For patients who co-enrol to the TRACC Trial (a multi-centre, prospective, translational study sponsored by the Royal Marsden NHS Foundation Trust) data generated from the molecular sub-classification of FFPE tumour tissue from pre-CRT biopsies and post-CRT resection specimens, described above, will be securely shared with the TRACC research team. In addition translational data, relating to CTDNA and re-sequencing results from FFPE tumour tissue, acquired in the TRACC trial for co-enrolled patients will be securely shared with the TRIGGER research team. This is to avoid duplication of work by research teams from the same sponsor and to optimise resource utilisation.

4. STUDY ENDPOINTS

PRIMARY ENDPOINT

- The association between pre-treatment CRC molecular subclassification with pathological and MR TRG response to rectal CRT

SECONDARY ENDPOINTS

- The association between rectal cancer molecular sub-classification with time to recurrence, disease free survival and overall survival
- The association between apoptotic and proliferation indices with response to CRT, time to local recurrence, disease free survival and overall survival
- The percentage of patients with locally advanced rectal cancer that have detectable ctDNA pre-operatively
- The association between detectable ctDNA at the first post-operative visit and disease free survival
- The concordance rate between mutations detected by targeted resequencing in tumour tissue and mutations detected by digital PCR in cfDNA

5. DATA ANALYSIS

The RNAseq data in “qseq” format will be aligned to the reference human genome using Maq and Bowtie tools. The software Cufflinks will be used to estimate the abundance of individual transcript in a sample. After this, we will select the 786 genes and their corresponding gene expression profiles for each sample

and perform Pearson correlation with our published prediction analysis of microarray (PAM) centroids, which are set of scores that define the subtype-specific gene associations. This analysis will assign each sample to a particular subtype. The significance of these classifications will be tested using *Chi-Squared* statistical test. Finally, the ability of the CRCassigner-786 genes, CRCassigner-30 genes and CRCassigner-7 genes to identify molecular subtypes of CRC with predictive value for CRT will be assessed. Furthermore, the association of CRT with the subtypes will be assessed using our in-house developed sparse factor analysis with covariates with 786 genes signatures as profile data and CRT and other clinical outcomes as covariates.

For massively parallel DNA sequencing analysis, Burrows-Wheeler Alignment (BWA) will be used to align reads to the human reference genome (GRGh37). PCR-derived duplicates will be removed from further analysis. Base quality re-calibration, realignment around indels, and variant calling will be performed using the Genome Analysis Tool kit (GATK) using the Broad best practice variant detection workflow. The MuTect algorithm will also be used to identify somatic single nucleotide mutations in targeted exons. The set of candidate somatic mutations will be refined using the following list of heuristic rules:

1. Variants detected at a minor allele frequency of greater than 0% in any of the 1000 Genomes project populations will be excluded from the set of somatic mutations
2. Variants called in regions not covered by the exome capture probes will be excluded.
3. Variants marked as low quality (QUAL below 20) will be excluded.
4. Candidate mutations will be cross-referenced with somatic mutations detected from the colorectal TCGA dataset.

For IHC analyses (proliferative and apoptotic markers), Quantitative data will be summarised using number of patients, mean values (or median when appropriate), standard deviation and minimum and maximum values. Qualitative data will be summarised using number of patients and percentages.

6. STATISTICAL CONSIDERATIONS

In order to computationally define CRC subtypes in this study, we will perform RNAseq (massively parallel sequencing) using a minimum sample size of 55, which was previously estimated using a pilot CRCassigner-786 gene profile data from 35 CRC tumour samples. Assuming a Bernoulli distribution of data, the statistical power was set at 0.8 and the false discovery rate at 5%.

APPENDIX 8: PROTOCOL AMENDMENT HISTORY

Summary of Amendments Made to the TRIGGER Protocol

Amendment to protocol (Version 1.0 to Version 2.0) during MHRA Review process following initial application to MHRA:

Document(s) Version and Date		Details of amendment (include page numbers where appropriate)	Brief rationale to support application for amendment
Current	Amended		
V 1.0 23/09/2015	V 2.0 10/11/2015	**Section 6.2, Exclusion Criteria (Page 10)**<br><br>Addition of text clarifying that contraceptive measures have to be compliant with the SmPCs for the trial IMPs.	Requested by MHRA following review of the initial application for a CTA in Notice for Grounds for Non-acceptance and Right to Amend Request letter dated 08/11/2015.
V 1.0 23/09/2015	V 2.0 10/11/2015	**Appendix 1, Section 2.7 Contraception & Pregnancy Reporting (page 66-67)**<br><br>Revision of text to clarify contraceptive requirements during the intervention phase of the trial, including removal of contraceptive methods not considered highly effective and addition of methods considered **highly** effective. Text added confirming contraceptive measures should be compliant with those described in the SmPCs for the trial IMPs.	Requested by MHRA following review of the initial application for a CTA in Notice for Grounds for Non-acceptance and Right to Amend Request letter dated 08/11/2015.
V 1.0 23/09/2015	V 2.0 10/11/2015	**Section 9.3 Dispensing and Drug Supplies (page 24)**<br><br>The following text was deleted from this section to confirm that a standard dispensing label will be used as the IMPs used in this trial each have a MA and are being used according to the terms of the MA:<br><br>*'Dispensed drugs will require 'clinical trial' labeling by the site pharmacy in compliance with regulatory requirements.'*	Requested by MHRA following review of the initial application for a CTA in Notice for Grounds for Non-acceptance and Right to Amend Request letter dated 08/11/2015. The labelling document submitted to the MHRA confirmed no example label was required but section 9.3 of V1.0 of the protocol included a statement that 'clinical trial' labelling was required in error.

Amendment to protocol (Version 2.0 to Version 3.0) during REC approval process following initial application to REC:

Document(s) Version and Date		Details of amendment (include page numbers where appropriate)	Brief rationale to support application for amendment
**Current**	**Amended**		
V 2.0 10/11/2015	V 3.0 26/11/2015	Addition of Trial Physician Dr Shelize Khakoo, Surgeon Mr Rasheed and Trial Research Nurse on page iii and iv.	New investigator and trial staff.
V 2.0 10/11/2015	V 3.0 26/11/2015	Change in frequency of TOXICITY and CHEMOTHERAPY CRF completion from every 6-week to after each cycle. Minor changes to relevant text in Section 8.1-8.3 (pages 18-24) and Assessment Schedules (Section 10) modified to reflect this change.	To reflect the frequency of toxicity assessment performed routinely during chemotherapy treatment.
V 2.0 10/11/2015	V 3.0 26/11/2015	Minor amendments including correction of typographical errors, name of MRI CRF, use of site code not site number (page 8), change in name of database from CRSweb to MACRO (page 43), clarification of labeling requirements for images, tissue and slides in Appendix 5 (page 97), confirmation of where translational data will be stored (appendix 7, page 105).	To correct typos included in Version 2.0, change of CRF name and labelling requirements for clarification, and to confirmation of database used and storage of translational data.

Non-substantial Amendment 1 – non-substantial changes to protocol (version 3.0 to Version 3.1)

**Document(s) Version and Date**		**Details of amendment (include page numbers where appropriate)**	**Brief rationale to support application for amendment**
**Current**	**Amended**		
**V 3.0 26/11/2015**	**V 3.1 18/01/2016**	Multiple pages: version number and date updated to Version 3.1 date 18/01/2016 in the document footer, title page and protocol signature page (page ii).	Non-substantial amendment.
**V 3.0 26/11/2015**	**V 3.1 18/01/2016**	Page 3, Section 3.2:<br>Original wording:<br>• tumour height (on MRI)<br>• the planned choice of systemic chemotherapy (CAPOX, FOLFOX, or single agent 5-FU or capecitabine)<br>New wording:<br>• tumour height (on MRI, height of tumour from anal verge ≤ 6 cm versus > 6 cm)<br>• the planned choice of systemic chemotherapy (CAPOX/FOLFOX versus single-agent 5-FU/capecitabine)	To clarify categorisation of randomisation stratification variables.
**V 3.0 26/11/2015**	**V 3.1 18/01/2016**	Page 12: Section 7.2<br><br>Original wording:<br>The patient's *date of birth* will also be used to cross-reference these samples.<br><br>New wording:<br>The patient's *initials* will also be used to cross-reference these samples.	The use of patient initials rather than date of birth on blood sample labels is consistent with other on-going GI Trials by the sponsor.
**V 3.0 26/11/2015**	**V 3.1 18/01/2016**	Throughout protocol, including Schedule of Events in Section 10.2 (pages 29-30) and Surveillance Schedule in Section 11.2.1 (Page 39): Change of name of case report form name from REGROWTH to SURVEILLANCE Form.<br><br>Surveillance Schedule Table, Section 11.2.1, page 39:<br>Addition of SURVEILLANCE Form to 'Completion of CRF' column.	For clarity so participating sites are aware that data capture/CRF completion during the surveillance period is not limited to recording of regrowths only.<br><br>To confirm that SURVEILANCE Form needs to be completed at each follow-up visit in patients who defer surgery.

		Addition of CRF number for clarity.<br><br>Section 11, page 37: case report form for capturing information relating to pelvic/distant relapse changed from REGROWTH form to ANNUAL FOLLOW-UP Form.<br><br>Section 11.3, page 41:<br>Amendment to text in third paragraph to clarify only data for suspected regrowth captured on SURVEILLANCE Form, not suspected disease recurrence.	Incorrect form name used in previous version. Relapse data captured on ANNUAL FOLLOW-UP Form.<br><br>To confirm only **suspected regrowth** data captured (Note: confirmed disease recurrence captured on annual follow-up form.)
**V 3.0 26/11/2015**	**V 3.1 18/01/2016**	Minor changes to text:<br><br>Page v – planned trial period changed from October 2015-July 2016 to March 2016-December 2016.<br><br>Change of contact details for Trial Staff and TMG members on pages iii –iv.	
**V 3.0 26/11/2015**	**V 3.1 18/01/2016**	**Deletion of text on page i:**<br><br>ISRCTN:<br>Date Registered	Trial registered on clintrials.gov
**V 3.0 26/11/2015**	**V 3.1 18/01/2016**	**Amendment to text on page ii relating to PI agreement:**<br>**Original wording:**<br>I agree to comply with the principles of Good Clinical Practice (GCP), the EU and GCP Directives (2001/20/EC; 2005/28/EC) and The Medicines for Human Use (Clinical Trials) Regulations 2004 and Amendment Regulations 2006 (Statutory Instrument 2006 No. 1928).<br>**New wording:**<br>I agree to comply with the principles of Good Clinical Practice (GCP), the EU and GCP Directives (2001/20/EC; 2005/28/EC) The Medicines for Human Use (Clinical Trials) Regulations 2004 (SI 2004/1031) and any subsequent amendments.	To clarify compliance with all subsequent amendments to EU and GCP directives and Clinical Trial Regulations.

Substantial Amendment 3 (MHRA and REC) – Amendment to protocol (Version 3.1 to 4.0)

Document(s) Version and Date: Current V3.1 18/01/2016 Amended V 4.0 19/07/2016	
**Details of amendment (include page numbers where appropriate)**	**Brief rationale to support application for amendment**
**Multiple pages**: version number and date updated to Version 4.0 date 19/07/2016 in the document footer, title page and protocol signature page (page ii).	Substantial amendment.
**Page iv**: addition of trial investigator	New information
**Page vi**: revision of text relating to planned feasibility trial period	Change in recruitment period date and to clarify recruitment period relates to feasibility phase of trial only
**Section 7.3.1 Screening, registration and randomisation logs, page 11:** addition of text relating to identification of potentially eligible patients	To confirm that potentially eligible patients can be identified on the baseline MRI report or by review of case notes, in addition to MDT meetings.
**Section 9.4 SmPC, page 24 and Section 2.3 Determining AE expectedness, page 60:** addition and revision of text relating to SmPC and reference safety information	To confirm that section 4.8 of the SmPC for each IMP represents the reference safety information and which versions of the SmPC should be used when assessing causality and expectedness.
**Section 10.2, Intervention arm – good responders, page 28:** addition of text relating to timeline for commencing chemotherapy	Timeline of ≤12 weeks not included in this schedule of assessment in previous version of protocol in error.
**Section 10.3, Intervention arm – poor responders, page 30:** change in timeline for commencing chemotherapy from ≤8 to ≤12 weeks.	Correction of typographical error.
**Section 12.7 Trial database, page 41:** revision of text relating to database and data entry on both CRF and database	For clarification and to remove text referring to the Hospital Information System, which related to a different type of database (CRS Web) rather than the database being used for this trial (Macro).
**Section 2.1.4 AESI, page 58:** revision of text relating to reporting of pCRM involvement	To remove the requirement of completion of an SAE form for pCRM involvement as information relating to pCRM involvement is being captured on the PATHOLOGY CRF. In addition this event does not fulfil the regulatory definition of a **serious** adverse event.
**Appendix 6 MRI protocol, EMVI Section, page 97:** addition of text relating to EMVI	To clarify definition of EMVI and distinction between small, medium and large vein invasion

Substantial Amendment 4 (MHRA and REC) – Amendment to protocol (Version 4.0 to 5.0)

Document(s) Version and Date: Current V4.0 19/07/2016 Amended V 5.0 07/03/2017	
**Details of amendment (include page numbers where appropriate)**	**Brief rationale to support application for amendment**
**Multiple pages**: version number and date updated to Version 5.0 date 07/03/2017 in the document footer, title page and protocol signature page (page ii).	Substantial amendment.
**TMG Group, list of investigators (page iv):** addition of contact details for new TMG member.	Addition of Co-Investigator, Mr Nader Francis, to TMG
**Trial Summary (page vi), Section 4.1 Primary objective (page 5), Section 14.1.2 Feasibility Sample Size (page 45):** planned feasibility trial period changed from March 2016-March 2017 to March 2016 – June 2018 (both include a 2-3 month patient registration period which occurs prior to patient randomisation)	To allow sufficient time to obtain important secondary endpoint data required to evaluate trial feasibility including assessing the safety of the deferral of surgery approach and the safety and acceptability of additional pre-operative therapy in good responders. The increase in duration will also allow further NHS and international sites to open to allow a more robust evaluation of potential patient recruitment rate in the planned Phase III trial.
**Trial Flowchart (pages v and 17), Section 7.1.2 PIS 2 Randomisation (page 11), Sections 10.1-10.3 Assessment Schedules (pages 26-31):** increase in time allowed for patient consent to randomisation from the last 2 weeks of CRT to anytime during CRT or once CRT is complete but at least 1 week prior to the post-CRT MRI scan.	Logistical: To facilitate patient recruitment at participating sites by increasing the time the trial teams have to discuss the trial with the patient and take informed consent. The rationale for also allowing consent to take place once CRT is complete (but prior to the post-CRT MRI scan) is to accommodate recruiting sites at which patients receive CRT at a satellite centre and will need to return to the recruiting site for informed consent.
**Trial Flowchart (pages v and 17), Section 8.2 Intervention arm – A 'Good Response' to CRT (page 19), Section 10.2 Intervention arm – 'Good Response' Assessment schedule (pages 28-29).** Addition of MRI scan performed mid-way through chemotherapy for patients who defer surgery**.**	For safety: to identify patients on the deferral of surgery pathway that have evidence of local regrowth during chemotherapy treatment.
**Section 10.4 Biomarker Development (page 32), Appendix 7, Section 3 (Page 105).** Addition of text relating to patients who co-enrol to the TRACC Trial and planned sharing of data between the TRIGGER and TRACC research teams.	To confirm arrangements relating to blood collection for patients who co-enrol to TRACC (multi-centre, translational trial) and confirm that there will be a sharing of specified translational data between the TRACC and TRIGGER research teams.
**Section 8.3 Experimental arm – a 'poor response to CRT (page 22, point 4), Section 10 Assessment schedules (points 2 and 5)**	Correction of typographical error and minor amendments to text for clarification.
