# Supplementary material for: A rectal cancer feasibility study with an embedded phase III trial design assessing magnetic resonance tumour regression grade (mrTRG) as a novel biomarker to stratify management by good and poor response to chemoradiotherapy (TRIGGER): study protocol for a randomised controlled trial
Source: Trials. 2017 Aug 29;18:394. doi: 10.1186/s13063-017-2085-2 (PMC5576102; doi:10.1186/s13063-017-2085-2)
Supplement: Supplementary file 2 — REGISTRATION Patient Information Sheet Version 1.1. (PDF 180 kb) [file 13063_2017_2085_MOESM2_ESM.pdf]

<To be printed on locally headed paper>

PATIENT INFORMATION SHEET (PIS2 - RANDOMISATION)

The TRIGGER trial

A trial looking at a magnetic resonance imaging biomarker to determine treatment after chemotherapy and radiotherapy in patients with cancer of the rectum

Invitation to take part in a research study called The TRIGGER trial

We are inviting you to take part in the main part of a clinical trial called TRIGGER because you are completing a course of chemotherapy with radiotherapy (CRT) for bowel (rectal) cancer and you may be able to enter this trial during your last cycle of CRT. You will remember that you kindly gave your consent to Step 1 of the TRIGGER Trial, which involved sending a specimen of your tumour to a central laboratory. The next step is to invite you to participate in the main part of the TRIGGER Trial.

This information sheet is intended to support your discussions with your oncology doctor and nurses. Please ask them if you are unclear about any of the points raised. When you have read this information sheet please take time to consider whether you wish to take part. You may want to discuss it with your GP, your family or others before deciding.

This patient information sheet is split into parts:

- A trial summary on page 2
- Part 1 tells you the purpose of this study and what will happen to you if you take part
- Part 2 gives you more detailed information about how the trial is being run.

Thank you for taking the time to read this information sheet and considering taking part in the main TRIGGER trial.

TRIAL SUMMARY

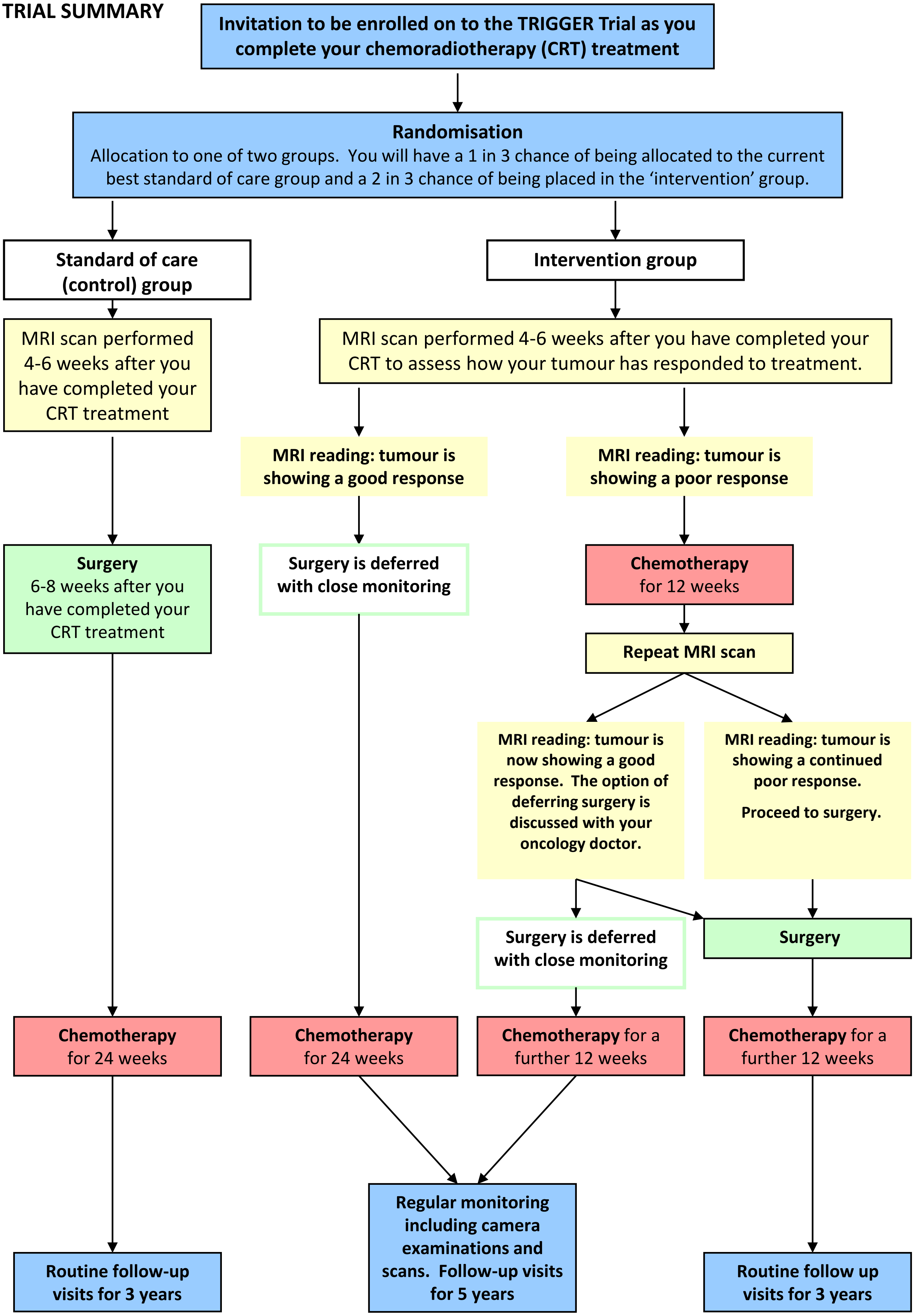

Part 1

Why is this trial being conducted?

> ***We would like to find out:***
> - *if we can use MRI scans to assess how a tumour has changed with chemoradiotherapy*
> - *if we can use the scan information to make decisions about surgery and further treatment*

Almost half of all patients diagnosed with rectal cancer are offered CRT before surgery. There is great variability in how each tumour responds to this treatment. We know this by looking at the cancer specimen after the surgery. Approximately 30% of tumours completely respond to CRT and no cancer cells can be found in the specimen removed at surgery - we hope that this trial will enable us to identify such patients so that they can avoid surgery altogether. Other tumours do not respond at all or occasionally continue to grow during treatment and we hope that maximising treatment for these patients will improve outcomes. Shortly after your CRT treatment your response to treatment is assessed by an examination and by performing CT and MRI scans. However, currently the MRI scan reading is not used to make decisions about further treatment plans. Emerging evidence suggests that by viewing the MRI scans in a smarter way it is possible to assess how the tumour has responded to treatment. This 'smarter' non-invasive technique is called the 'mrTRG' (MRI Tumour Regression Grade).

The aim of the TRIGGER trial is to evaluate if this non-invasive imaging technique can be used to assess response to treatment and be used to make decisions about further treatment. Everyone who agrees to take part will be randomly allocated to either the current best standard of care of surgery at 6-8 weeks after completion of radiotherapy (***standard of care group***) or an 'intervention' group where your further care will be based on the MRI tumour regression grade assessment (split into good response or poor response sub-groups depending on the MRI scan reading).

Do I have to take part?

No, it is up to you to decide if you want to take part. If you decide to take part, we will give you this sheet to keep and we will ask you to sign a consent form to say that you understand what the trial involves. If you choose to enter this trial, you may withdraw from the trial at any time. You will still receive the best treatment your oncology doctor can offer you. If you have private medical insurance, you should consult with your insurer before agreeing to take part. Your participation in this trial is entirely voluntary. If, after considering it, you decide not to participate, this will not affect the standard of your care in any way and your oncology doctor will explain the best treatment available.

How will the research be done?

The best way of weighing up the advantages and disadvantages of different treatments for a particular type of cancer is in a Randomised Controlled Trial, or RCT. The decision about which

treatment you will receive is random – or based on chance rather like the flip of a coin. A computer will decide which group you are allocated to - these options would not be selected by you or your oncology doctors. This is called randomisation. Using the computer to randomise patients into groups ensures that the groups of patients are similar. In this way, a fair comparison can be made between the trial groups at the end of the trial. If you take part, you will have a 1 in 3 chance of receiving the current best standard of care and a 2 in 3 chance of receiving a treatment plan based on the MRI assessment of how well your tumour has responded.

What will happen to me if I take part?

- ***You will be randomised into 1 of 2 groups.***
- ***Your treatment plan will vary according to which group you are placed in.***

As described above if you decide to take part in this trial you will be randomised to one of the following groups at the start of the study:

1. Current best standard of care (control) group
2. An 'intervention' group. Patients allocated to this group will be split into one of two subgroups based on the MRI assessment of how their tumour has responded to CRT:
    a. MRI scan reading shows a 'good' tumour response.
    b. MRI scan reading shows a 'poor' tumour response.

Your treatment plan will vary according to which of the above groups you are randomised to. Each of these is described below and summarised in the flow chart on page 2. As allocation to group 1 or 2 is made by a randomisation process described above it is important that you and your oncology doctor are happy for you to accept the care and treatment plans associated with each of the groups. However, your Doctor will discuss treatment plans at each stage of the trial, and in some circumstances you and your Doctor may decide that your treatment plan should differ to that described below. This ensures that the treatment plan you receive is tailored to you. If you decided to participate in the study your oncology doctor will be able to tell you which of the groups you are in shortly after you enrol in the trial.

1. What will happen if I am randomised to the current best standard of care (control) group?

If you are randomised to the control group your treatment plan is the same as standard practice for your type of bowel cancer:

- *Complete chemoradiotherapy*
- *Surgery*
- *24 weeks of chemotherapy*

The current standard of care for patients with your type of bowel cancer is to offer chemoradiotherapy followed by surgery. Once you have recovered from surgery (usually 4-6 weeks later), the majority of patients are then offered 6 months of chemotherapy. In most hospitals, a second MRI scan is performed before your surgery to see how well the chemoradiotherapy has worked but the information is not used to alter your treatment plan. The type of surgery that you will undergo and any further treatment will already have been decided on the basis of the scan done when you were initially diagnosed with bowel cancer. Therefore if you are allocated to this group you will undergo surgery 6-8 weeks following the completion of your

chemoradiotherapy and then receive 24 weeks of chemotherapy (called adjuvant chemotherapy) – please see the flow chart on page 2.

What are the possible risks or benefits of taking part if I am randomised to this group?

Because you will have been allocated to receive the current best standard of care there are no additional risks associated with being in this trial. All treatments will be undertaken using the best standards available and there will be checks in place to ensure that this is occurring.

2. What will happen if I am randomised to the 'intervention' group?

__If you are randomised to the Intervention arm you will be placed into 1 of 2 groups depending on how well your tumour responded to chemoradiotherapy__

If you are allocated to this group the MRI scan performed at the end of your CRT treatment will be assessed to see how well your tumour has responded to treatment (using the new special way of measuring mrTRG described at the top of page 2). Patients will be split into two subgroups depending on if the MRI reading shows your tumour has had a 'good' or a 'poor' response to CRT treatment. The treatment plan is different for these two subgroups and each of these is described below and summarised in the flow chart on page 2:

a. What will happen if the MRI scan shows my tumour has responded well to chemoradiotherapy and I am placed in the 'good' response subgroup?

__If your scan shows your tumour responded very well then you will be able to defer surgery. We will monitor you closely at outpatient appointments using camera examinations and scans. Your treatment plan would be:__

- *Complete chemoradiotherapy*
- *24 weeks of chemotherapy*
- *Intensive monitoring (see p14)*

If you are in this subgroup the scans may show that you have had an excellent response and the bowel cancer has either completely disappeared or is starting to disappear and will shrink with time. This happens in up to a third of patients after CRT. In this situation, it is still routine to have surgery at 6-8 weeks after CRT followed by chemotherapy. However in the trial you will be able to either delay the surgery until the cancer stops reducing in size or avoid surgery altogether if the cancer cannot be detected with repeat scans and assessment. If you decide to take part in this trial, you will still be offered the chemotherapy that was originally planned (and will receive the same amount of chemotherapy as patients being treated according to routine standards). If you and your oncology doctor decide to defer surgery we will monitor your tumour scar closely using a combination of assessment by your surgeon in the outpatient clinics and MRI scans so that surgery can be performed if the tumour either starts to regrow or stops shrinking (please see flow chart on page 14).

What are the possible benefits of deferring surgery?

If you have no visible cancer detected, in a proportion of patients it may be possible to avoid surgery altogether which means that you may avoid some potential side effects of surgery and may avoid a permanent stoma bag.

What are the possible disadvantages of deferring surgery?

The main risk of deferring surgery is that there may be cancer left behind which cannot be detected by examination or scans. We have addressed this by including intensive regular follow-up (summarised on page 17). The evidence available suggests we are able to detect any re-growth at an early stage, at which point surgery will be offered immediately. Taking part in the trial ensures that we do this safely and with all the rigorous checks and measures to ensure that if the tumour does not completely disappear or starts to show signs of regrowing that you proceed to have the surgery.

b. **What will happen if the MRI scan shows that my tumour has not responded well to chemoradiotherapy and I am placed in the 'poor' response subgroup?**

If you are in this subgroup the scans show that your tumour has not responded well to treatment and the risks of the tumour coming back or spreading further are high. In this situation it would be routine to still have surgery followed by chemotherapy, but if you decide to take part in the trial you will be able to receive chemotherapy sooner in order to try to control the spread of cancer before surgery takes place rather than waiting until after you have recovered from surgery.

If your scan shows your tumour has not responded well to treatment then you will receive chemotherapy before surgery. Your treatment plan would be:

- *Complete chemoradiotherapy*
- *12 weeks of chemotherapy*
- *Surgery*
- *12 weeks of chemotherapy*

What are the possible benefits of receiving chemotherapy sooner?

It is possible that receiving chemotherapy sooner may reduce the size of the tumour further and lower the risk of the cancer spreading. However this has not yet been proven and is something that we are investigating in this trial.

What are the possible disadvantages or risks of receiving chemotherapy sooner?

It is possible that increasing the amount of chemotherapy you receive prior to surgery, by completing your planned chemotherapy and radiotherapy treatment and then receiving 12 weeks of chemotherapy, may increase the number of side effects you experience during treatment. A recent study of patients with rectal cancer who received additional chemotherapy prior to surgery showed that the number of side effects was not increased but this cannot be guaranteed as larger studies are required. We will be assessing this in the TRIGGER Trial by recording and evaluating side effects that patients may experience during the trial. There is a small risk that your tumour may not respond, or may even progress during the time you receive chemotherapy and remain difficult to operate on successfully.

What will happen once I have completed the 12 weeks of chemotherapy?

Once you have completed 12 weeks of chemotherapy you will have further MRI scan. **This MRI scan will be used to make a decision about the most appropriate next step in your treatment plan** (please see flow chart on page 2):

- If the MRI scan reading shows that your tumour hasn't changed then you will have surgery as planned, followed by the remaining 12 weeks of chemotherapy once you have recovered from surgery.
- If the MRI scan reading shows that your tumour has responded well to the additional chemotherapy then your oncology doctor will discuss the option of deferring surgery with you. If the decision is that surgery should be performed then you will undergo surgery and receive the remaining 12 weeks of chemotherapy once you have recovered. If the decision is that surgery can be deferred then you will receive the remaining 12 weeks of chemotherapy and we will monitor your tumour scar closely using a combination of assessments by your surgeon at outpatient clinics and MRI scans so that surgery can be performed if the tumour either starts to regrow or stops shrinking. The possible benefits and risks associated with deferring surgery are described above on page 6.

If I participate in the TRIGGER trial what chemotherapy will I receive?

The current standard of care for patients with rectal cancer is a 6 month course of chemotherapy, which starts shortly after surgery. All patients who participate in the TRIGGER trial will be treated with the most effective chemotherapy available for rectal cancer, which we know will help reduce the chance of the cancer coming back, and both you and oncology doctor will know which treatment you are receiving. The TRIGGER trial is not a 'placebo' trial meaning there are no 'dummy' treatments. The treatments are widely used and have already been shown to be safe and effective.

In the TRIGGER trial, doctors can choose either one of the similar types of chemotherapy drug regimes based on either capecitabine or fluorouracil, used alone or in combination with Oxaliplatin. When used in combination it is given either as:

- ***We are not testing any new drugs in this trial.***
- *The chemotherapy drugs you will receive are the most effective treatments for bowel cancer.*
- *All patients receive a total of 24 weeks of chemotherapy as is standard.*
- *Patients in the intervention arm who a poor response to chemoradiotherapy will have 12 weeks of chemotherapy before surgery and 12 weeks of chemotherapy after surgery*

1) A drug combination called "FolFox". This is a standard chemotherapy that has been tried and tested in many thousands of patients. We know that it is one of the most effective treatments for colorectal cancer and it is one of the types of chemotherapy recommended by the National Institute for Clinical Excellence (NICE). The treatment starts with two chemotherapy drugs, called oxaliplatin and folinic acid, given as a "drip" into a vein over two hours. After that, another drug called fluorouracil is given very slowly into the vein, over the next 46 hours. This 48-hour treatment is given twelve times at 2-week intervals amounting to 24 weeks of treatment in all. There are several different methods of giving FolFox and your doctor or nurse will discuss with you the way that suits you best. Most commonly it is given at home, using a small portable pump. To receive "FolFox" chemotherapy, you will need to have a thin flexible tube fitted in either your arm or your chest. This leads into one of your veins, and chemotherapy is given through it. Once fitted, it avoids many needles, and can stay in for the duration of your treatment.

Or

2) as a drug combination called "CapOx"

Again, this is a standard chemotherapy known to be one of the most effective treatments for colon cancer that has been tried and tested in many thousands of patients. The two drugs used are Oxaliplatin and a drug that acts in a similar way to fluorouracil and folinic acid called Capecitabine. Oxaliplatin is given as a "drip" into a vein over two hours and capecitabine is a pill taken twice daily for two weeks afterwards. Eight courses of CapOx are given at 3-week intervals, again amounting to 24 weeks of treatment in total.

For some patients, the oxaliplatin component will be omitted but your doctor will discuss this with you before starting any treatment, along with which of these two treatments is to be recommended in your case, and what it involves.

When will I receive chemotherapy?

The timing of when you will receive chemotherapy varies according to which group you are placed in (please see flow chart on page 2):

Current best standard of care (control) group

If you are randomised to the current best standard of care group you will receive standard chemotherapy following the surgery. That is to say, there is a 1 in 3 chance that if you decide to participate in TRIGGER you will have an operation 6-8 weeks after completing your chemoradiotherapy, then a 4-8 week period of recuperation, then a 24-week course of chemotherapy, as described above.

Intervention group - 'Good' response

If you are placed in this group you will start chemotherapy treatment 6-8 weeks after completing chemoradiotherapy, and will receive a standard course of 24 weeks of chemotherapy.

Intervention group - Poor response

If you are placed in this group you will receive 12 weeks of chemotherapy prior to the decision about proceeding to surgery. This would be followed by a rest period of 3-4 weeks, to allow the treatment to have its full effect and for any side effects to settle. Then if your treatment plan includes surgery you will receive the remaining 12 weeks of chemotherapy once you have recovered from surgery, normally about 4-8 weeks later. In other words you will receive the same amount of chemotherapy as those patients in the other groups but you will receive half before surgery and the other half following surgery. If your treatment plan is to defer surgery then you will receive the remaining 12 weeks of chemotherapy shortly after your repeat MRI scan.

What are the possible side effects and risk associated with receiving chemotherapy?

Whether or not you take part in the TRIGGER study, the recommended treatment for your condition would involve surgery and a course of chemotherapy. There are small risks from any form of major surgery and chemotherapy

As with all drugs it is possible you will experience some side effects during chemotherapy. The main side effects are included in this section and your doctor will provide further information about these treatments before you start.

can also produce unwanted effects. Your doctor will explain in detail what these treatments involve and provide information leaflets about these as appropriate. Some patients get no side-effects, but it is helpful to be forewarned of some of the more common side effects associated with FolFox or CapOx chemotherapy, which are described below:

• For a few hours or days after starting treatment, you may feel tingling ('pins and needles') in the hands and feet if you touch cold things or go out in the cold. You may also feel tingling in the throat. This is harmless and will settle without treatment for most patients. Occasionally the numbness and problems with cold weather can persist in the long-term.

• Just occasionally, people can become allergic to one of the drugs, though this is rare. If, while the drip is running, you develop a racing heart beat, an itchy rash, wheezing or a swollen tongue, please tell the nurses immediately.

• Chemotherapy can cause diarrhoea. You will be given anti-diarrhoea tablets to use if this is mild, but if you have severe diarrhoea (more than 4 watery stools in a day) please telephone the hospital for advice.

• Some patients find they feel a little sick for a few days after starting treatment, but vomiting is unusual. You will be provided with some anti-sickness tablets to take if you start feeling sick. If you vomit more than once in a 24-hour period, please telephone the hospital for advice.

• Some people notice soreness in the mouth or a change in taste for some foods. You will be provided with a mouthwash which may help. If you develop ulcers or pain in the mouth, please telephone the hospital for advice.

• Some people feel more tired than usual during chemotherapy treatment. There is no easy answer to this, but if you are affected you may find it helps to set aside a rest period in the middle of each day.

• Any chemotherapy treatment may temporarily reduce your resistance to infections, so if you develop a high temperature or other symptoms of infection between treatments, you may need to come to the hospital for an urgent check-up.

• Occasionally, we meet someone who is particularly sensitive to the effects of chemotherapy and has more severe side-effects than expected. If that happens, treatment is stopped until the problems have settled; it is usually then possible to continue treatment at a lower dose.

• Women of childbearing potential must have a negative pregnancy test prior to trial entry and avoid pregnancy during and for 12 months after chemotherapy. Men with a partner of childbearing potential must use adequate, medically approved, contraceptive precautions during and for 12 months after the last dose of chemotherapy. If applicable, your Doctor or Nurse will discuss this in more detail with you prior to commencing chemotherapy.

During the time that you receive chemotherapy your Doctor or Nurse will ask you about any side effects that you experience and any new medications that you start. These discussions will normally take place at a routine clinic appointment but your Nurse may also need to contact you by phone. It can be useful to record details of any side effects or changes to medications and your Nurse can provide you with a study diary to do this if you wish.

What if my chemotherapy treatment doesn't work for me?

Your chemotherapy treatment will be offered for as long as you seem to be doing well on it. The chemotherapy will continue if the scans show that the cancer has shrunk or if it has not grown. If

your cancer grows, then normally you will stop the treatment you are on, as there is no benefit. If this happens you will stop the chemotherapy and your oncology doctor will talk to you about other treatment options.

What are the possible risks associated with scans I may have during the trial?

As part of the routine follow-up of patients diagnosed with rectal cancer, CT and PET-CT scans are frequently performed. These scans involve exposure to ionising radiation (x-rays for CT scans and gamma rays for PET scans), which allows images to be produced of the internal structures of the body. If you decide to participate in this study you will not undergo any more CT or PET-CT scans than you would if you did not take part. Ionising radiation can cause cell damage which may, after many years or decades turn cancerous. The risk of this happening to you is very small, and will be the same whether you participate in this study or not. If you have any questions or concerns about these scans then please discuss these with your oncology doctor.

Will this trial help patients in the future?

The main purpose of this trial is to find out whether it is better to treat patients with your type of cancer by going straight to surgery after their initial treatment with chemoradiotherapy or whether we can personalise treatment based on how well the tumour has responded, with avoidance of surgery in patients that have responded well and additional treatment for those with tumours that have responded poorly. If we can answer this, it may be beneficial to future patients in your situation. We will be finding out whether this approach will work well in practice, namely assessing the response to treatment first, and then allocating treatment depending on the individual's response. At the same time we will be examining the tumour tissue and blood tests to see if there are new ways to treat cancer to help select future treatments better.

What will happen to any samples I give and will any new genetic tests be done?

- ***With your permission we would like to store a piece of your tumour and blood samples for research***
- ***These samples will be anonymised***
- ***We will use these samples to find out more about bowel cancer and investigate how a persons genetic makeup influences response to***

We would like your permission to send a piece of your tumour to a central laboratory if you have surgery. We are also asking you for extra blood samples. These tumour and blood samples will be used for further research that will help us understand more about bowel cancer and the type of treatment that might be more effective for other patients in the future. It will involve extracting DNA or other material from the piece of your tumour and from your blood. This will allow us to look into the way each person's genetic makeup influences the way they respond to any medical treatment – how well the treatment works and what side effects occur. Quite often there are traces of cancer DNA that can be found circulating in blood and if we can reliably detect these traces it may be possible to remove the need for biopsy tests to monitor changes in the cancer during and after treatment in the future.

If you consent to the collection of additional blood samples for the research described above then we will collect a total of 20 ml (equivalent of 4 teaspoons) on up to 12 occasions during the study.

We hope to take additional samples at the same time as those taken for your routine care to avoid extra blood tests or extra visits. Blood tests can be associated with a small area of bruising on your skin where the needle goes in but any bruising should disappear after a few days.

Your tumour and blood samples will be sent to a central laboratory based at the Royal Marsden NHS Foundation Trust where they will be stored securely. These samples will be anonymised – labelled with only your unique trial number, not your name. Once the study is complete the anonymised samples will be sent to four laboratories (3 within the UK and 1 in the Netherlands) for analysis. The results of the analysis may be shared with collaborators at the Royal Marsden NHS Foundation Trust who are carrying out similar research on patients with bowel cancer. These additional studies will not affect your treatment in any way, and you are free to withhold this permission without affecting your participation in TRIGGER or your relationship with your doctor.

Your consent form will ask whether you are happy to provide these additional blood samples and tumour samples for these areas of research. If you do not want to give permission for these samples, you can still participate in the TRIGGER trial.

Thank you for taking the time to read this information and for considering taking part in TRIGGER. Please feel free to keep this information sheet. If you are interested in taking part, please read Part **2** of this information sheet for further general information on participating in this Trial**.**

Part 2

Who is organising and funding this research?

This multi-centre feasibility trial, involving approximately 90 patients, is being sponsored by the Royal Marsden NHS Foundation Trust and is funded by the Pelican Cancer Foundation. This trial is being organised by the TRIGGER Trial Office at the Royal Marsden based in Sutton. The Royal Marsden NHS Foundation Trust is responsible for ensuring that this study is done properly.

What if relevant new information becomes available?

During the course of the trial, which is expected to last several years, the progress of the research will be considered at regular intervals by an expert committee. Sometimes during the course of a research project, new information becomes available about the treatment that is being studied or the tests we are doing. If this happens, your oncology doctor will tell you about it and discuss with you whether you want to continue in the study. If you decide to stop the trial treatment plan, your oncology doctor will make arrangements for your care to continue. If you decide to continue in the study you will be asked to sign an updated consent form. In some cases a decision may be made to stop a trial early and you will be informed of this should this happen and arrange for your continuing care.

What will happen if I don't want to carry on with the study?

Your participation in this study is voluntary and you are able to withdraw from the study at any time and without any reason. If you do so, your participation in the study will end and the study staff will stop collecting information from you. We would retain your blood samples and information collected before you withdrew from the study and continue to use it confidentially and only for the purpose described in this information sheet. If you choose to withdraw or your research doctor withdraws you from the study we may ask if we can continue to collect limited information about your overall health status until the trial ends. You will not need to be contacted or disturbed for collection of this information and we would only do this is you provide consent for us to do so at the time you withdraw from the study.

What if there is a problem?

Every care will be taken in the course of this clinical trial. If you have a concern about any aspect of the study, you should ask to speak with your oncology doctor who will do their best to answer your questions. If you remain unhappy and wish to complain, or have any concerns about any aspect of the way you have been approached or treated by members of staff or about any side effects (adverse events) you may have experienced due to your participation in the clinical trial the normal National Health Service complaints mechanisms are available to you. Please ask your oncology doctor if you would like more information on this. Details can also be obtained from the Department of Health website: http://www.dh.gov.uk.

In the event that something does go wrong and you are harmed during the research and this is due to someone's negligence then you may have grounds for a legal action for compensation against the Royal Marsden NHS Foundation Trust but you may to have to pay your legal costs.

Will my taking part in this study be kept confidential?

If you join this trial, some parts of your medical records and the data collected for the study will be looked at by authorised persons from the TRIGGER Trial Office at the Royal Marsden Hospital who are organising the research and where all information will be collected. They may also be looked at by representatives of regulatory authorities and by authorised people to check that the study is being carried out correctly. All will have a duty of confidentiality to you as a research participant and we will do our best to meet this duty.

All information that is collected about you during the course of the research will be kept strictly confidential. A copy of your consent form, which will include your name and unique trial number only, will be sent to the Royal Marsden Hospital Trials office who will be responsible for running this trial. Your GP will also be informed of your participation in the trial. All other information about you that leaves the hospital will have your name and address removed so that you cannot be recognised. No individual patients will be identified when the results of the trial are published.

What will happen to the results of the research study?

We will publish the results of this trial in a medical journal. We will send a copy of the results to your doctor. You will not be identified in any report or publication. We will also publish a summary of the results of this study, in plain English, on the pelican Cancer Foundation website. The address is http://www.pelicancancer.org/ A copy of the results will be available to you or your family from your clinic on request.

The regulatory authorities have given their permission for this trial to be undertaken and we will report to them in confidence any unexpected side-effects which occur.

Who has reviewed the study?

All research that involves NHS patients or staff, information from NHS medical records or which uses NHS premises or facilities must be approved by an NHS Research Ethics Committee before it goes ahead. Approval means that the Committee is satisfied that your rights will be respected, that any risks have been reduced to a minimum and balanced against possible benefits, and that you have been given sufficient information to make an informed decision to take part or not. This study has been reviewed and given a favourable opinion by the London – Surrey Research Ethics Committee which means the study can go ahead.

Where can I get further information

If you have any further questions about your illness or this trial, please discuss them with your oncology doctor or research nurse. Their contact details are:

Local Contact names and telephone numbers:

Local Investigator: Dr ………………………………………… Telephone no: ………………………………………

Research Nurse: ……………………………………………… Telephone no: ……………………………………….

Thank you for taking the time to read this information and for considering taking part in this trial. Please feel free to keep this information sheet. If you decide to take part in TRIGGER, you will be asked to sign a consent form and you will be given a copy of the signed consent form to take home. Please use this space to write down any questions you have for your doctors.

Monitoring schedule for patients who defer surgery

Year 1

Chemoradiotherapy (CRT) completed

↓

Chemotherapy for 24 weeks
MRI scan mid-therapy

↓

6 month visit
Clinical assessments
MRI Scan
Flexible Sigmoidoscopy

↓

9 month visit
Clinical assessments
MRI Scan
Flexible Sigmoidoscopy

↓

12 month visit
Clinical assessments
MRI Scan & CT Scan
Colonoscopy
Quality of life questionnaire

Year 2

15 month visit
Clinical assessments

↓

18 month visit
Clinical assessments
MRI Scan
Flexible Sigmoidoscopy

↓

21 month visit
Clinical assessments

↓

2 year visit
Clinical assessments
MRI Scan & CT Scan
Flexible Sigmoidoscopy
Quality of life questionnaire

Years 3-5

2 years & 6 months visit
Clinical assessments

↓

3 year visit
Clinical assessments
MRI Scan & CT Scan
Flexible Sigmoidoscopy
Quality of life questionnaire

↓

3 years & 6 months visit
Clinical assessments

↓

4 year visit
Clinical assessments
MRI Scan
Flexible Sigmoidoscopy

↓

4 years & 6 month visit
Clinical assessments

↓

5 year visit
Clinical assessments
MRI Scan
Colonoscopy
Quality of life questionnaire

Clinical Assessments – these include a blood test and digital rectal examination during an outpatient visit. The routine blood test measures a tumour marker called CEA, which is used in combination with other tests to detect any re-growth of the tumour at an early stage. The digital rectal examination is a type of physical examination during which a doctor inserts a finger into your rectum (back passage) to feel for abnormalities. This only takes a few minutes and isn't usually painful.

Flexible sigmoidoscopy/Colonoscopy – these are camera examinations during which a long, thin and flexible tube is inserted into your rectum. A sedative is often given to help you relax.

MRI & CT scans – non-invasive scans that are used to produce detailed images of inside your body.

Quality of life questionnaire – this will take about 10 minutes to complete and includes questions about your health and about your bowel function. The form will be anonymised with only your trial ID number and initials included.
