# Supplementary material for: A rectal cancer feasibility study with an embedded phase III trial design assessing magnetic resonance tumour regression grade (mrTRG) as a novel biomarker to stratify management by good and poor response to chemoradiotherapy (TRIGGER): study protocol for a randomised controlled trial
Source: Trials. 2017 Aug 29;18:394. doi: 10.1186/s13063-017-2085-2 (PMC5576102; doi:10.1186/s13063-017-2085-2)
Supplement: Supplementary file 3 — RANDOMISATION Patient Information Sheet Version 4.0. (PDF 410 kb) [file 13063_2017_2085_MOESM3_ESM.pdf]

<To be printed on locally headed paper>

# Patient Information Sheet and Consent Form (PIS1 - Registration)

## The TRIGGER trial

## A trial looking at a magnetic resonance imaging biomarker to determine treatment after chemotherapy and radiotherapy in patients with cancer of the rectum

## Invitation for patients eligible for registration into The TRIGGER trial

### 1. Why am I being given this patient information sheet?

You are being invited to take part in a research study called TRIGGER because your oncology doctors and surgeons are recommending or have already started you on a course of chemotherapy together with radiotherapy (this is called : CRT) which is given for rectal (bowel) cancer. Participation in the research is entirely voluntary. If after considering it, you decide not to participate, this will not affect your care in any way.

### 2. Why am I being asked to participate in this research?

It is known that patients with bowel cancer can benefit from CRT (chemotherapy with radiotherapy) prior to surgery as it enables shrinkage of the tumour and improves the likelihood of successful surgery. Your oncology doctor is recommending that you have CRT to reduce the size of your tumour. Shortly after your CRT treatment your response to treatment is assessed by an examination and by performing CT and MRI scans. Currently the standard treatment for patients with bowel cancer is to perform surgery following CRT treatment. However there is great variability in how each tumour responds to the CRT treatment. We know this by looking at the bowel tissue removed during surgery. Approximately 30% of tumours completely respond to treatment and no cancer cells can be seen. Other tumours do not respond at all or occasionally continue to grow during treatment. Emerging evidence shows that by viewing the MRI scan performed after CRT treatment in a smarter way it is possible to assess how the tumour has responded to treatment. This information may be used to refine your treatment further. In the TRIGGER trial we plan to be able offer you a treatment based on how well the tumour has initially responded to CRT according to the MRI scan reading.

## 3. What am I being asked to consent to?

To take part in this research study you will be asked to consent to two separate steps. We have provided a flow chart at the end of this information sheet to show the steps more clearly. The first step is explained here and step 2 is described in section 4 of this information sheet.

Step 1 asks for your permission to send a sample of your tumour (already stored in the local hospital pathology department where your cancer was diagnosed) to a central laboratory. This piece of tumour was obtained when you were first diagnosed as part of the camera examination. A copy of your consent form will be sent to your local hospital pathology department (where it will be kept confidential and in a secure location), to permit the release of your pathology blocks to the central laboratory.

In addition to the blood samples that will be taken as part of your standard care, we would also like your permission to take some further blood for other bowel cancer research purposes. This will help us find substances in your blood which might help us understand more about bowel cancer and the type of treatment that might be more effective for other patients in the future. It will not help us with your own treatment. Your consent form will ask whether you are happy to provide these initial blood samples for research. We hope to take additional samples at the same time as those taken for your routine care so you do not have to undergo an extra test or visit. A total of 20 ml (equivalent of 4 teaspoons) of blood will be collected for this purpose. Blood tests can be associated with a small area of bruising on your skin where the needle went in but any bruising should disappear after a few days. We will ask you in the second stage of the trial about the collection of further samples.

We would like to assess your quality of life at the start of your treatment. This will take the form of three short questionnaires about your bowel function and general health that would take approximately 15 minutes to complete. These would be given to you when you attend the clinics.

We are also asking your permission to collect future routine information about your health status after participation in the trial. Data about your overall health status will be collected by adding your name to government national registers such as the Office for National Statistics (ONS) and the NHS Strategic Tracing Service. If you have given your consent you will not need to be contacted or disturbed for this limited data about your overall health status to be submitted to the trial centre.

All data will be kept safe in accordance with requirements of the UK Data Protection Act (DPA).

A copy of your consent form, which will include your name and unique trial number, will also be sent to the Trial Office at the Royal Marsden which is responsible for running this trial.

**What will happen to any samples I give?**

We would like your permission to store your blood samples and the piece of your tumour for further research that will help us understand more about bowel cancer and the type of treatment that might be more effective for other patients in the future. It will involve extracting DNA or other material from the piece of your tumour and from your blood. This research is based in UK Universities which will involve collaboration with researchers within the Royal Marsden NHS Foundation Trust and at other institutions. All such work is anonymous: your specimens will be identified by your unique trial number, not your name. These additional studies will not affect your treatment in any way, and you are free to withhold this permission without affecting your participation in TRIGGER or your relationship with your doctor.

**Step 1 consent:** There is a question about each of the above on the consent form that we will ask you to sign before you agree to be registered into TRIGGER.

## 4. Information about Step 2:

**What will happen to me at the end of my chemoradiotherapy?**

The second step of this research study will start at the end of your 6 weeks of CRT. The aim of the TRIGGER trial is to determine whether the MRI reading of how much your tumour has responded to CRT can be used to individualise treatment and improve the overall success of the treatment of patients with bowel cancer.

**What if I do not want to participate at 6 weeks?**

By consenting to this part of the trial, including permission to send a sample of your tumour to a central laboratory, you are under no obligation to consent to participate in the main TRIGGER trial at the end of your CRT. If you decide not to consent at step 2, we would hope to be able to use the results from your tests performed at the central laboratory for other research. However, you can ask for the results not be given to anyone else or used in any way. Please use this space below to record any questions you might have for your oncology doctor at your next visit.

## Flow Chart for TRIGGER Step 1 - Registration

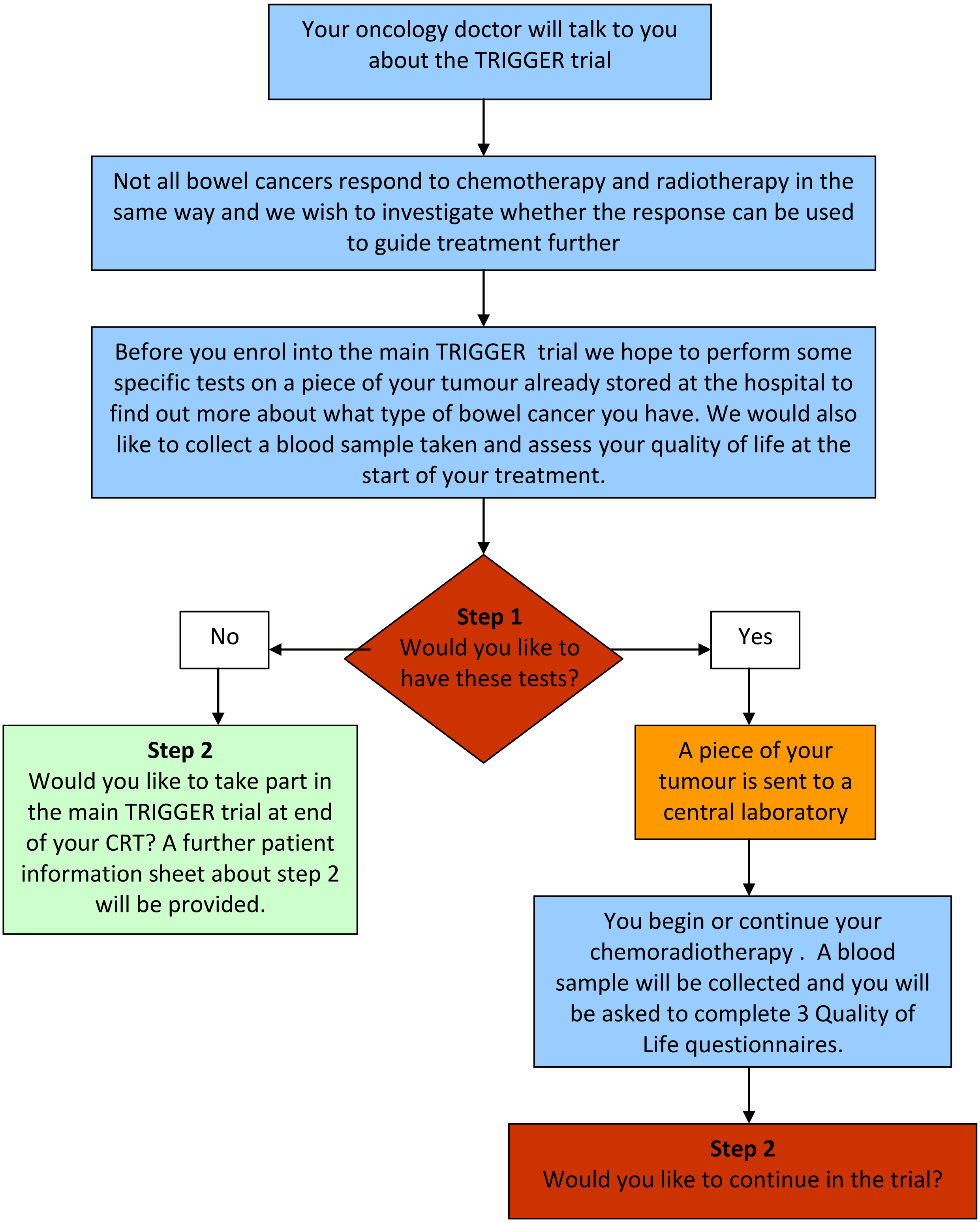


**Contact Details for further information:**

Local Investigator: Dr.............................................. Telephone No: ..............................

Research Nurse: ...................................................... Telephone No: ..............................
